# Magnetic hysteresis in 1D organometallic lanthanide chain compounds containing 4,4′-bipyridine

**DOI:** 10.1039/d5sc05460e

**Published:** 2025-09-29

**Authors:** Ernesto Castellanos, Florian Benner, Saroshan Deshapriya, Selvan Demir

**Affiliations:** a Department of Chemistry, Michigan State University 578 South Shaw Lane East Lansing Michigan 48824 USA sdemir@chemistry.msu.edu

## Abstract

The assembly of multinuclear complexes bearing highly anisotropic building blocks remains an attractive approach to developing advanced functional materials. However, incorporating lanthanide-based metallocenium moieties, [Cp^R^_2_Ln]^+^, into higher-order systems remains a significant synthetic challenge and their targeted isolation is exceedingly rare. Presented herein are organometallic lanthanide chain compounds bearing bridging 4,4′-bipyridine ligands, 
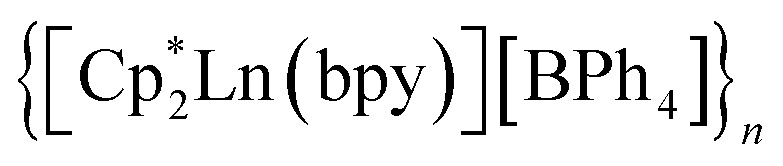
 (where Ln = Gd (1), Tb (2), Dy (3); Cp* = pentamethylcyclopentadienyl; bpy = 4,4′-bipyridine). This constitutes the first report of a crystallographically characterised 1D organometallic network of lanthanide metallocenium units connected to one another through organic bridges. Each metallocenium moiety is ligated by two bipyridyl ligands, giving rise to zigzag-shaped chains, where tetraphenylborate anions reside in between the nitrogen ligands. The formation of the compounds from 
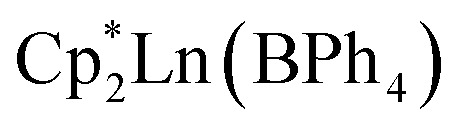
 and 4,4′-bipyridine is very fast, leading to an immediate precipitation in the polar solvent THF. Thus, a judicious synthetic route was developed to ensure crystallisation and pure isolation which involved the use of an H-tube. Dc magnetic susceptibility measurements for 1–3 allude to the presence of uncoupled lanthanide ions, which is consistent with the experimental cw-EPR spectrum as well as the calculated magnetic exchange coupling constant, *J*, for the gadolinium congener, 1, obtained through broken-symmetry DFT. The dysprosium analogue, 3, is a single-molecule magnet (SMM) which was confirmed through both out-of-phase ac magnetic susceptibility signals under a zero applied dc field, indicative of slow magnetic relaxation, and isothermal, variable-field dc measurements, revealing open magnetic hysteresis loops up to 8 K. The lack of intra- and interchain magnetic exchange suggests that the origin of single-molecule magnetism in 3 arises from single-ion anisotropy and crystal field, which is further supported *via ab initio* calculations.

## Abstract 2

Unprecedented organometallic lanthanide chain compounds containing 4,4′-bipyridine bridges were isolated, and in-depth studied *via* single-crystal X-ray diffraction, magnetometry, spectroscopy, DFT and *ab initio* computations.

## Graphical abstract

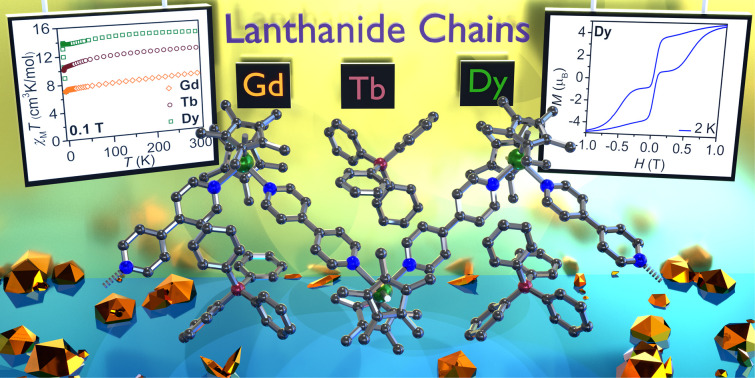

## Introduction

Metal-containing polymers, named metallopolymers, have emerged as a promising class of advanced materials with widespread applications in nanoscience and biomedicine, owing to the unique physical and chemical properties conferred by the incorporation of metal centres.^1–6^ Specifically, metallopolymers with reversible redox behaviour hold significant promise for applications in electrocatalysis,^7–10^ sensing,^11–15^ responsive surfaces,^16^ and as integral components in photonic crystal displays. A diverse range of different metal ions can be employed to tune macromolecular properties, including p-, d-, and f-block metals. Suitable linkages that bind the metal centres in metallopolymers are heteroorganic donor scaffolds that can vary from strong dative bonds to weak and labile, non-covalent coordination interactions that allow for reversible, so-called metallosupramolecular binding. The dimensionality of metallopolymers ranges from 0D (*e.g.*, complexes, macrocycles), 1D structures such as nanowires or polymeric network-like infinite coordination polymers,^17^ 2D nanosheets,^18,19^ or 3D structures, most commonly referred to as porous coordination polymers (PCPs)^20^ and metal–organic frameworks (MOFs).^21^ Lanthanide (Ln)-containing metallopolymers have received considerable attention due to the unique luminescent and paramagnetic properties of Ln ions.^22,23^ However, the construction of Ln chain compounds is challenging due to the less directional 4f metal centres relative to d-block metals. Notably, organometallic lanthanide chains, where the metal ions exhibit true metal–carbon interactions and ligation to one another through organic bridges, remain hitherto elusive. Homoleptic organolanthanide coordination polymers, {(Cp_2_Ln)(μ-Cp)}_*n*_ (where Cp = cyclopentadienyl, Ln = La, Pr, Lu),^24–26^ feature short and long metal–Cp interactions, but suffer from a lack of tunability. In principle, a strategy to devise organometallic Ln chain compounds is to use both bulky Cp ligand scaffolds to accommodate the large Ln^III^ ions, and bridging nitrogen-based ligands. To this end, the highly tuneable lanthanide metallocenium unit, [Cp^R^_2_Ln]^+^ is promising as two Cp^R^ rings engender a high metal coordination number while leaving open metal sites to enable construction of a 1D chain through nitrogen donors of another ligand. In addition, the inclusion of metal ions innate to high magnetic anisotropy may lead to functional magnetic materials.

Lanthanide ions are particularly exciting in this context as their unquenched orbital angular momentum and strong spin–orbit coupling affords large magnetic anisotropies – a prerequisite for the design of single-molecule magnets (SMMs). An SMM is a molecule that can retain a preferred orientation of the magnetic moment in the absence of an external magnetic field. The SMM's property of magnetic memory is appealing for future technologies in high-density information storage,^27^ quantum information science,^28–30^ and spintronics.^31,32^ This bulk magnet-like behaviour observed on a molecular level in SMMs relies on magnetic relaxation proceeding through discrete energy states of an energy barrier to spin relaxation. Notably, metallocenium scaffolds, where two Cp hydrocarbons impart an axial crystal field onto oblate lanthanide ions, have produced the best mononuclear SMMs.^33–37^ In fact, per judicious design, dysprosocenium complexes of this type [Cp^R^_2_Dy]^+^ (where R = polyalkyl or trialkylsilyl) led to unparalleled magnitudes of effective spin-reversal barriers (*U*_eff_) and blocking temperatures (*T*_B_), even exceeding liquid nitrogen temperatures.^38^

Adjusting the steric bulk of the bis-Cp scaffold and appropriate organic bridges that contain donor atoms may pave the way to higher nuclearity systems. Those may be innate to large spin ground states if efficient magnetic exchange coupling between paramagnetic centres is fostered. The deeply buried 4f-orbitals of the Ln^III^ ions require the implementation of strong magnetic exchange coupling that can be chemically achieved through direct metal–metal bonds,^39^ radical bridges,^40–42^ and heavy p-block elements.^43–46^

These approaches ushered in various landmark advances regarding a deeper understanding of magnetic exchange mechanisms,^47^ fundamental f-element and main group chemistry,^48–50^ single-molecule magnetism,^39^ and applied spectroscopy.^41,49,51–53^ The assembly of multiple [Cp^R^_2_Ln]^+^ moieties yielded only zero-dimensional molecular architectures so far.^54–63^ To reach higher order systems, bidentate N-donor ligands such as 4,4′-bipyridine (bpy) offer a unique platform. Bpy may act as a bridge to two metal ions, while only occupying one metal coordination site which has been demonstrated in lanthanide–halide and lanthanide–β-diketonate complexes.^64–69^ Exposure of the bulky trismetallocenes, Cp^R^_3_Ln (where Ln = Ce, Nd, R = C_5_H_4_SiMe_3_)^70,71^ to 4,4′-bipyridine yielded dinuclear bipyridine-bridged complexes, 
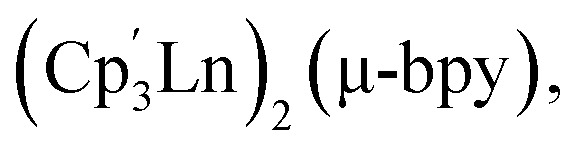
 where the formation of an extended polymeric network is mitigated by the steric bulk of the tris-Cp framework.

Herein, the synthesis and characterisation of organometallic lanthanide chain complexes, 
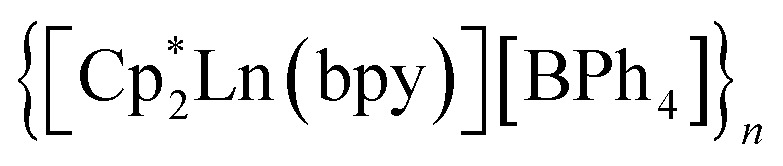
 (where Ln = Gd (1), Tb (2), Dy (3); Cp* = pentamethylcyclopentadienyl; bpy = 4,4′-bipyridine), bearing 4,4′-bipyridine bridges, is presented. 1–3 correspond to the first series of organometallic lanthanide chain compounds comprising bridging bidentate N-donor ligands, which enable the assembly of the 1D network. The identities of 1–3 were determined through single-crystal X-ray diffraction analysis, and revealed 1D layers of lanthanide metallocenium moieties, each ligated to two 4,4′-bipyridyl ligands, functioning as bridges to adjacent Ln^III^ ions. 3 shows slow magnetic relaxation under zero field, indicative of SMM behaviour, which is attributed to the single-ion effect. Remarkably, 3 exhibits open magnetic hysteresis loops up to 8 K. The synthesis of this series of complexes elicits a new methodology for implementing organometallic building blocks of high magnetic anisotropy into well-defined 1D extended networks. In addition, the nature of redox-active organic bridges may provide paths in the future to boost exchange coupling by accessing radical states as a gateway to advanced magnetic and spintronic materials.

## Experimental methods

### General information

All manipulations were performed in a nitrogen-filled MBraun glovebox with an atmosphere of <0.1 ppm O_2_ and <0.1 ppm H_2_O. House nitrogen was purified through an MBraun HP-500-MO-OX gas purifier. Tetrahydrofuran (THF) was refluxed over potassium for several days and subsequently dried further over a Na/K alloy. THF was subsequently distilled and transferred to a nitrogen-filled glovebox. THF was tested for the presence of water and oxygen in the glovebox by the addition of one drop of potassium benzophenone radical solution to 2 mL of THF. Anhydrous gadolinium chloride (GdCl_3_), terbium chloride (TbCl_3_), dysprosium chloride (DyCl_3_), and allylmagnesium chloride (2.0 M in THF) were purchased from Sigma-Aldrich and used as received. 4,4′-Bipyridine (bpy) was purchased from Sigma-Aldrich and sublimed prior to use. Potassium bis(trimethylsilyl)amide (KN[Si(CH_3_)_3_]_2_) was purchased from Sigma-Aldrich, dissolved in toluene, centrifuged, filtered, and crystallised at −35 °C prior to use. 1,2,3,4,5-Pentamethylcyclopentadiene (HCp*) was purchased from Sigma-Aldrich and dried over 4 Å sieves prior to use. KCp*,^72^ [HNEt_3_][BPh_4_],^73^ and 
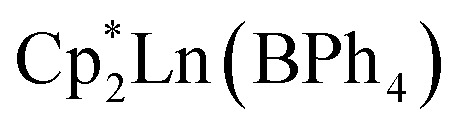
 (where Ln = Gd, Tb, Dy)^74,75^ were prepared according to literature procedures. Elemental analysis was performed at Michigan State University, using a PerkinElmer 2400 Series II CHNS/O analyser.

#### General synthesis of 
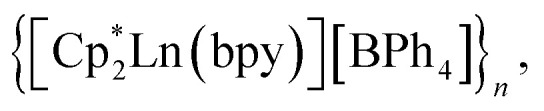
1–3

**Fig. 1:**
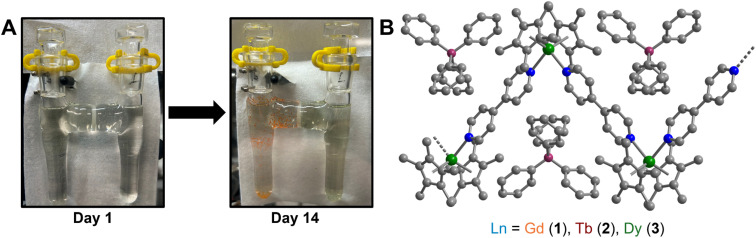
(A) Glassware used for the crystallisation of 
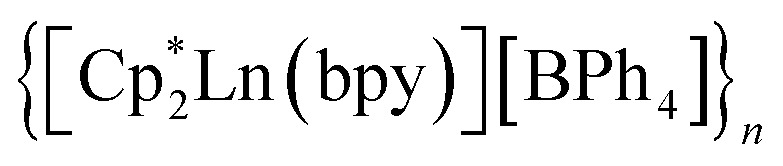
 (where Ln = Gd (1), Tb (2), and Dy (3)). The glass columns are bridged by a fine porosity frit, which enables crystallisation of 1–3*via* slow diffusion. (B) Polymeric structure of 
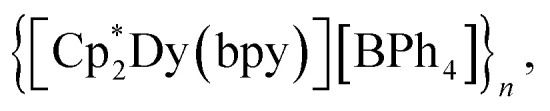
3, in a crystal of 
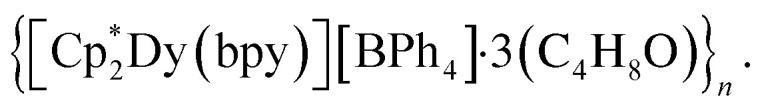
 H atoms and solvent molecules in the crystal lattice have been omitted for clarity. Green, blue, grey, and purple spheres represent Dy, N, C, and B atoms, respectively. Select distances (Å) and angles (°): Ln–C_Cp*_: 2.676(10)–2.743(24) (1); 2.633(12)–2.747(26) (2); 2.649(19)–2.711(21) (3); Ln–Cnt: 2.423 (1); 2.395 (2); 2.392 (3); Ln–N: 2.488(6) (1), 2.447(7) (2), 2.444(5) (3); Cnt–Ln–Cnt: 131.7 (1), 132.3, (2), 133.0 (3); N–Ln–N: 91.1(2) (1); 91.5(2) (2), 90.6(2) (3); Ln–Ln–Ln: 91.7(1) (1), 91.3(1) (2), 91.8(1) (3); Ln–N–N–Ln: 180.0(1) (1–3); Ln–B–Ln–B: 180.0(1) (1–3) (where B is the boron atom of the (BPh_4_)^−^ counteranion); Ln–Ln–Ln–Ln: 180.0(1) (1–3).

In a nitrogen-filled glovebox, the lanthanide tetraphenylborate complex, 
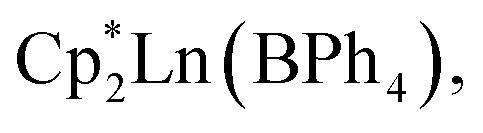
 was added to a 20 mL scintillation vial charged with a stir bar, and dissolved in 5 mL of THF, yielding a clear, pale-coloured solution (Gd = pale yellow-green; Tb = pale yellow-green; Dy = pale yellow). In a separate 20 mL vial, 4,4′-bipyridine (bpy) was dissolved in 5 mL of THF to give a clear, colourless solution. Each solution was then carefully filtered into separate glass tubes which are bridged by a fine porosity glass frit in the shape of an “H” (see Fig. 1A and S1). Each side of the H-tube was filled to the same level and was carefully layered with an additional 5 mL of fresh THF. Each ground glass joint was sealed air-tight, and the H-tube was stored upright at room temperature to allow for the reaction of the two reagents through slow diffusion. Within five days, the formation of small, crystalline orange blocks was observed. The diffusion was left to proceed undisturbed for two weeks yielding single-crystals suitable for single-crystal X-ray diffraction analysis. 1, 2, and 3 crystallised with three THF solvent molecules in the lattice as 
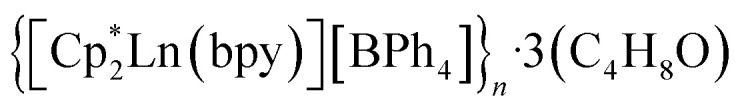
 (where Ln = Gd (1), Tb (2), and Dy (3)). The crystals were separated from the mother liquor, washed three times with 1 mL of fresh THF, and dried prior to spectroscopic, magnetic, and elemental analysis.

#### Synthesis of 
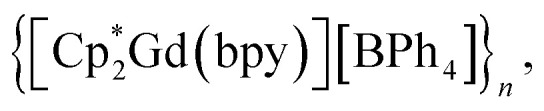
1

Masses used: 
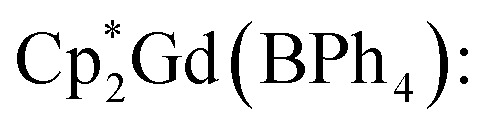
 41.0 mg, 0.055 mmol, 1.0 equiv.; 4,4′-bipyridine: 9.1 mg, 0.058 mmol, 1.0 equiv. Crystalline yield: 24% (15.0 mg, 0.013 mmol) as orange, block-shaped crystals. IR (ATR, cm^−1^): 3053w, 2981w, 2903w, 2860w, 1605m, 1580w, 152w, 1478w, 1426m, 1412m, 1381w, 1264w, 1218w, 1181w, 1114w, 1064w, 1031w, 1006m, 910w, 840w, 811m, 744sh, 732s, 704s. Anal. calc. for C_54_H_58_BN_2_Gd·3(C_4_H_8_O): C: 70.81; H, 7.38; N, 2.50. Found: C, 70.39; H, 7.15; N, 2.20.

#### Synthesis of 
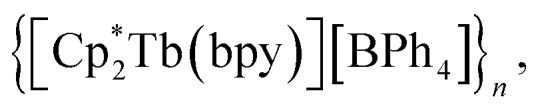
2

Masses used: 
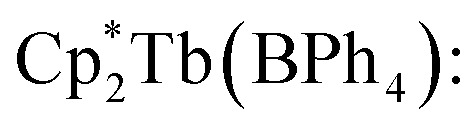
 49.0 mg, 0.065 mmol, 1.0 equiv.; 4,4′-bipyridine: 10.6 mg, 0.068 mmol, 1.0 equiv. Crystalline yield: 53% (39.2 mg, 0.035 mmol) as orange, block-shaped crystals. IR (ATR, cm^−1^): 3053w, 2981w, 2903w, 2860w, 1605m, 1580w, 152w, 1478w, 1426m, 1412m, 1381w, 1264w, 1218w, 1181w, 1114w, 1064w, 1031w, 1006m, 910w, 840w, 811m, 744sh, 732s, 704s. Anal. calc. for C_54_H_58_BN_2_Tb·3(C_4_H_8_O): C: 70.71; H, 7.37; N, 2.50. Found: C, 70.86; H, 7.48; N, 2.18.

#### Synthesis of 
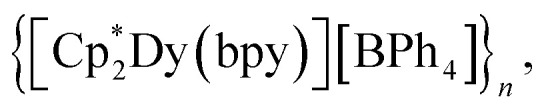
3

Masses used: 
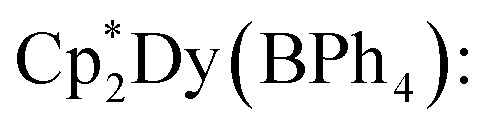
 45.3 mg, 0.060 mmol, 1.0 equiv.; 4,4′-bipyridine: 9.4 mg, 0.060 mmol, 1.0 equiv. Crystalline yield: 36% (24.1 mg, 0.021 mmol) as orange, block-shaped crystals. IR (ATR, cm^−1^): 3051w, 2981w, 2903w, 2859w, 1604m, 1579w, 1477w, 1425m, 1411m, 1380w, 1263w, 1218w, 1181w, 1114w, 1064w, 1030w, 1005m, 837w, 811m, 745sh, 731 s, 702 s. Anal. calc. for C_54_H_58_BN_2_Dy·3(C_4_H_8_O): C, 70.48; H, 7.35; N, 2.49. Found: C, 70.90; H, 7.20; N, 1.96.

### Single-crystal X-ray diffraction

Orange, block-shaped crystals with dimensions of 0.262 × 0.168 × 0.148 mm^3^, 0.206 × 0.154 × 0.103 mm^3^, and 0.228 × 0.153 × 0.13 mm^3^ for 1, 2, and 3, respectively, were mounted on a nylon loop using Paratone oil. Data for 1–3 were collected on a XtaLAB Synergy, Dualflex, HyPix diffractometer equipped with an Oxford Cryosystems low-temperature device, operating at *T* = 99.8(9) K, 100.0(1) K, and 100.0(1) for 1, 2, and 3, respectively. Data for 1 and 2 were measured using *ω* scans with Mo Kα radiation (microfocus sealed X-ray tube, 50 kV, 1 mA). Data for 3 was collected using *ω* scans and Cu Kα radiation (microfocus sealed X-ray tube, 50 kV, 1 mA). The total number of runs and images was based on the strategy calculation from the program CrysAlisPro (Rigaku, V1.171.41.90a, 2020), which was employed to retrieve and refine the cell parameters, as well as for data reduction. A numerical absorption correction based on Gaussian integration over a multifaceted crystal model empirical absorption correction using spherical harmonics was implemented in the SCALE3 ABSPACK scaling algorithm. The structures of 1–3 were solved in the space group *C*2/*c* by using intrinsic phasing with the ShelXL structure solution program.^76^ The structures were refined by least squares using version 2018/2 of XL^76^ incorporated in Olex2.^77^ All nonhydrogen atoms were refined anisotropically. Hydrogen atom positions were calculated geometrically and refined using the riding model.

### Infrared spectroscopy

IR spectra were recorded with an Agilent Cary 630 FTIR spectrometer on crushed crystalline solids under an inert nitrogen atmosphere.

### EPR spectroscopy

Continuous wave electron paramagnetic spectroscopy (cw-EPR) was recorded on a Bruker EMX-plus spectrometer operating at X-band frequencies. The spectrometer is equipped with a Bruker ER4119HS probe and a modified Bruker liquid nitrogen variable temperature accessory. Undiluted, crushed crystalline solids were packed into 4 mm OD quartz EPR tubes. Cw-EPR spectra were collected at 110 K, under 24 dB microwave attenuation and 0.5 G modulation amplitude, with 9.33 GHz microwave frequency. All simulations were executed using the ‘pepper’ module of the EasySpin 5.2.36 software package for Matlab.^78^

### Magnetic susceptibility measurements

Magnetic susceptibility data were collected on a Quantum Design MPMS3 superconducting quantum interference device (SQUID) magnetometer. The magnetic samples of 1–3 were prepared by saturating and covering dried, crushed crystalline solids (9.0 mg, 1; 21.3 mg, 2; 20.9 mg, 3) with ample molten eicosane (at 60 °C) to prevent crystallite torquing and to provide good thermal contact between the sample and the bath. The samples were sealed in an airtight container and transferred to the magnetometer. The core diamagnetism was estimated using Pascal's constants.^79^

### Computational methods

Broken-symmetry calculations of 
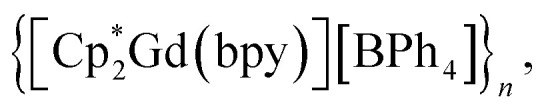
1, were performed with unrestricted density functional theory (DFT) calculations using ORCA 5.0.3 software^80,81^ with the uB3LYP functional^82,83^ employing the zeroth order regular approximation (ZORA)^84^ for relativistic treatment. The ZORA-def2-TZVP basis set was employed for all atoms and the DKH-def2-TZVPP basis set was used for the treatment of N atoms.^85^ Segmented all-electron relativistically contracted (SARC) basis set with quadruple zeta quality SARC2-DKH-QZVP was applied for the treatment of Gd.^86^ Where applicable, SARC-ZORA-TZVPP basis set was used for Y atoms as described in the Computational insights section.^87^ The calculations employed the SARC/J auxiliary basis^88,89^ and the reformulated Grimme's D3 dispersion correction with Becke–Johnson damping (D3BJ).^90^ Unoptimised crystal structure coordinates were used for the broken-symmetry calculations. The spin density distribution was generated using the orca_plot module and the VMD program^91^ was used for visualisations.

### *Ab initio* calculations

The magnetic properties of 
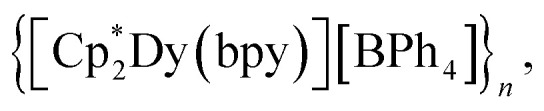
3, were calculated *via* a Complete Active Space Self-Consistent Field (CASSCF) + N-Valence Electron Perturbation Theory (NEVPT2) approach using the ORCA 5.0.4 software.^80,81^ Calculations were carried out for a single repeating unit, namely 
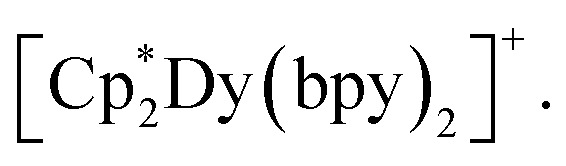
 Prior to CASSCF calculations, the hydrogen positions were optimized *via* DFT methods, using the TPSSh functional and basis set combinations as described for the CASSCF calculations below.

Scalar relativistic effects were taken into account *via* the Douglas–Kroll approach, where the DKH-def2-SVP basis set was used for the H atoms,^92^ DKH-def2-TZVP for the C atoms of the first coordination sphere and the bpy ligands.^93^ The SARC2-DKH-QZVP basis set was employed for the Dy atom.^94^ Auxiliary basis sets were generated *via* the autoaux feature.^95^ Tight convergence criteria were employed throughout with an energy convergence tolerance of 1 × 10^−7^. The computation of Fock matrices and gradient/Hessian integrals was accelerated by using the RIJCOSX approximation. The frozen core approximation was switched off for all calculations. Throughout all calculations a finer integration grid (defgrid 3) was employed. The active space comprised the 9 electrons of Dy^III^ in seven 4f orbitals. 21 quintet, 128 triplet, and 130 doublet roots were considered for the state averaged (SA) CASSCF calculation. Dynamic correlation effects were introduced into the spin-free SA-CASSCF wavefunction *via* strongly contracted NEVPT2 (SC-NEVPT2).^96–98^ The construction of the fourth order reduced density matrix was simplified *via* the efficient implementation (D4step efficient).^99,100^ Spin–Orbit-Coupling (SOC) effects were included within the NEVPT2 step *via* Quasi-Degenerate Perturbation Theory (QDPT) using the mean-field/effective potential Hamiltonian RI-SOMF(1x).^101–103^ The free-particle Foldy–Wouthuysen (fpFW) transformation was carried out in the first step of the DKH protocol by including the vector potential. Picture change corrections were included on the second order, as well as finite nucleus corrections.^104^ Lastly, the magnetic properties such as *g* tensors, crystal field parameters, and the estimated single-ion anisotropy barrier were calculated *via* the SINGLE_ANISO standalone program.^105^

## Results and discussion

### Synthesis and structural characterisation

The equimolar reaction of 
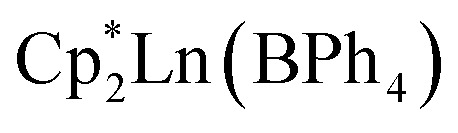
 (Ln = Gd, Tb, Dy) and 4,4′-bipyridine (bpy) in THF results in the instant formation of orange powdery solids, which are insoluble in polar organic solvents, as well as hydrocarbons such as ^*n*^hexane, benzene, and toluene. Thus, a judicious synthetic route was developed to ensure crystallisation and pure isolation of the new products by using a so-called H-tube, which consists of two glass columns that are bridged by a fine porosity glass frit (Fig. 1A and S1, see Experimental methods for details). The H-tube essentially slows down the reaction by facilitating the slow diffusion of THF solutions of 
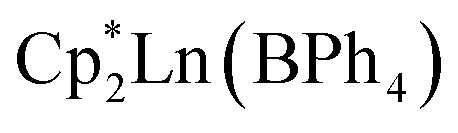
 and bpy, allowing the direct growth of single-crystals. Within the confines of a rigorously air- and moisture-free nitrogen-filled glovebox, the controlled diffusion across a glass frit over the course of two weeks at room temperature yielded orange, block-shaped single-crystals of 
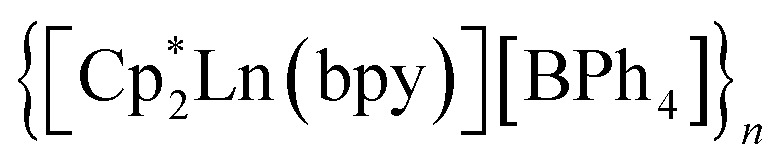
 (where Ln = Gd (1), Tb (2), Dy (3)), in 24%, 53%, and 36% crystalline yield for 1–3, respectively (eqn (1)). Single-crystal X-ray diffraction analysis uncovered isostructural lanthanide complexes that crystallise in the monoclinic *C*2/*c* space group, with asymmetric units composed of a disordered [Cp*Ln] motif, coordinated by one half of two bpy ligands (Table S1). In addition, half of a non-coordinating tetraphenylborate (BPh_4_)^−^ anion, as well as the halves of three non-coordinating THF molecules are present in each asymmetric unit. Examining the full solid-state structure reveals the first examples of 1D organometallic lanthanide (Ln) chain compounds, with the trivalent lanthanide ion ligated by two (η^5^-Cp*) rings and two 4,4′-bipyridyl ligands. Importantly, each bipyridyl ligand forms a bridge between two adjacent 
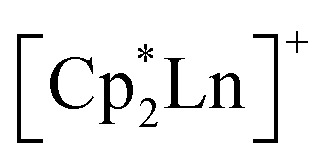
 moieties, giving rise to zigzag-shaped structures with non-coordinating (BPh_4_)^−^ filling the void of each triangular unit (Fig. S2, S10 and S18). These zigzag-shaped 1D architectures pack neatly into layers, where distances between neighbouring chain layers range from 9.099(1)–15.244(1) Å, 8.932(1)–15.354(1) Å, to 8.949(1)–15.364(1) Å for 1–3, respectively (Fig. S4–S9, S12–S17 and S20–S25).


1

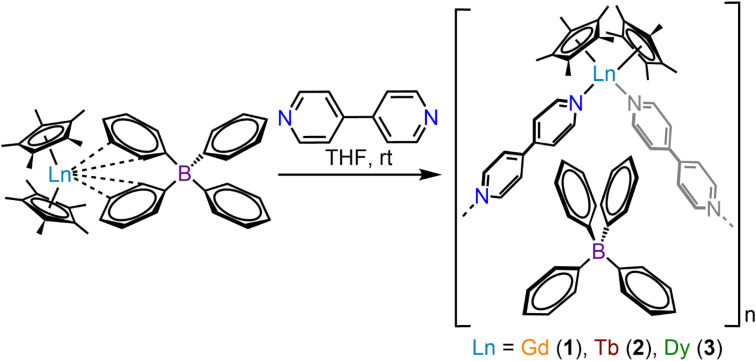



Within the lanthanide-based chains, symmetric Ln–N distances of 2.488(6) Å, 2.447(7) Å, and 2.444(5) Å are observed for 1–3, respectively, where the change in metal–ligand distance is consistent with the decrease in ionic radii for eight-coordinate Ln^III^ ions traversing from Gd^III^ over Tb^III^ to Dy^III^.^106^ The Ln–N distance is also in agreement with the respective distances in rare earth (RE) metallocenium moieties bearing neutral N-donor ligands, such as 
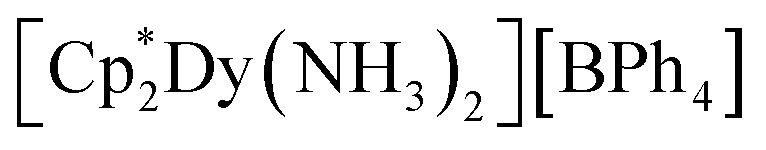
^107^ and 
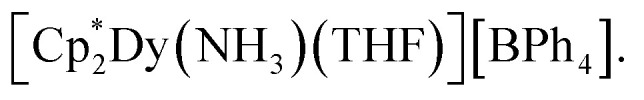
^107^ Notably, 1–3 exhibit long intrachain Ln⋯Ln distances of 12.209(1) Å, 12.111(1) Å, and 12.063(1) Å for 1–3, respectively. The considerable separation between Ln^III^ ions, in conjunction with the presence of a closed-shell bridging bipyridyl ligand, precludes strong magnetic exchange coupling between lanthanide centres.

**Fig. 2:**
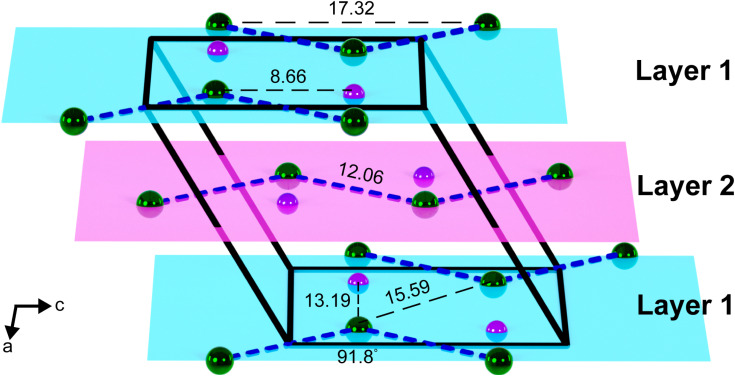
Simplified unit cell depiction of 
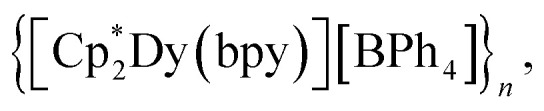
3, in a crystal of 
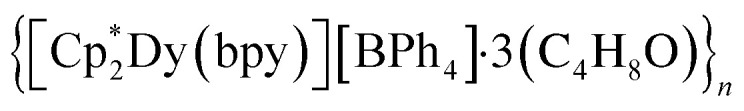
 along the *ac* plane illustrating the organisation of the layers with respect to each other. Green and purple spheres represent Dy and B atoms (where B is the boron atom of the (BPh_4_)^−^ counteranion), respectively. The dashed blue lines represent the bridging bipyridyl ligands. N, C, and H atoms, as well as solvent molecules in the crystal lattice have been omitted for clarity. Distances and angles are given in Å and deg.

Similarly, the Cnt–Ln–Cnt angles (where Cnt = Cp* ring centroid) of 131.7°, 132.3°, and 133.0° for 1–3, respectively, increase moving from Gd^III^ to Tb^III^ to Dy^III^ suggesting that the decrease in ionic radii enforces greater axiality of the metallocene framework to compensate for the change in electron–electron repulsion within the bis(Cp*) scaffold. This is further evidenced by the decrease in Ln–Cnt distances for 1–3 of 2.423 Å, 2.395 Å, and 2.392 Å, respectively. Notably, the bite angle of 3 is smaller than the one in the ammonia complex as well as the haloarene adducts of 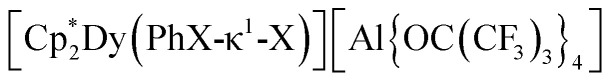 (where X = F, Cl), and 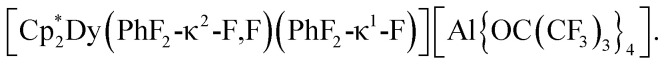
^108^ This decrease in axiality of the bis(Cp*) framework leads to elongated Dy–Cnt distances in 3 by approximately 0.03 Å and 0.1 Å compared to the corresponding distances in the ammonia and haloarene complexes, respectively. The changes in Cnt–Dy distances and the resulting Cnt–Dy–Cnt angle can be ascribed to the increased steric bulk of the bpy ligands. However, despite the differing size of N-donor ligands, the N–Ln–N angles of 91.1(2)°, 91.5(2)°, and 90.6(2)° for 1, 2, and 3, respectively, are marginally larger than the average angle of 88.2° observed for 
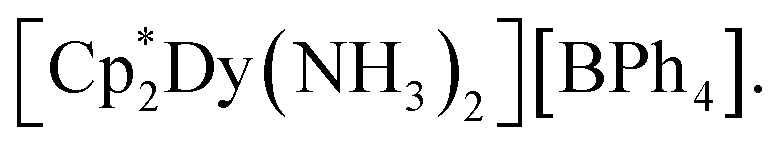
 Looking down one chain, the Ln–Ln–Ln angle is 91.7(1)°, 91.3(1)°, and 91.8(1)° for 1–3, respectively, while the torsion angle between four consecutive Ln^III^ ions is exactly 180.0° (Fig. 1 and 2, and Table S2). The planar arrangement of each chain is further validated by the Ln–B–Ln–B (where B is the boron atom of the (BPh_4_)^−^ counteranion) and Ln–N–N–Ln dihedral angles, which are also found to be 180.0° for 1–3 (Fig. 2). To minimize electronic repulsion arising from close H⋯H contacts between bipyridyl rings, the coordinating bipyridyl ligands remain tilted, leading to an angle between the planar pyridyl rings of 48.7(2)°, 46.2(2)°, and 45.1(2)°, for 1–3, respectively. The differences in crystallographic distances and angles, in conjunction with the isostructural nature of 1–3, result in superimposable vibrational spectra, where the fingerprint regions are dominated by bands associated with the 4,4′-bipyridyl ligand (Fig. S26–S30).

### Magnetic characterisation

**Fig. 3:**
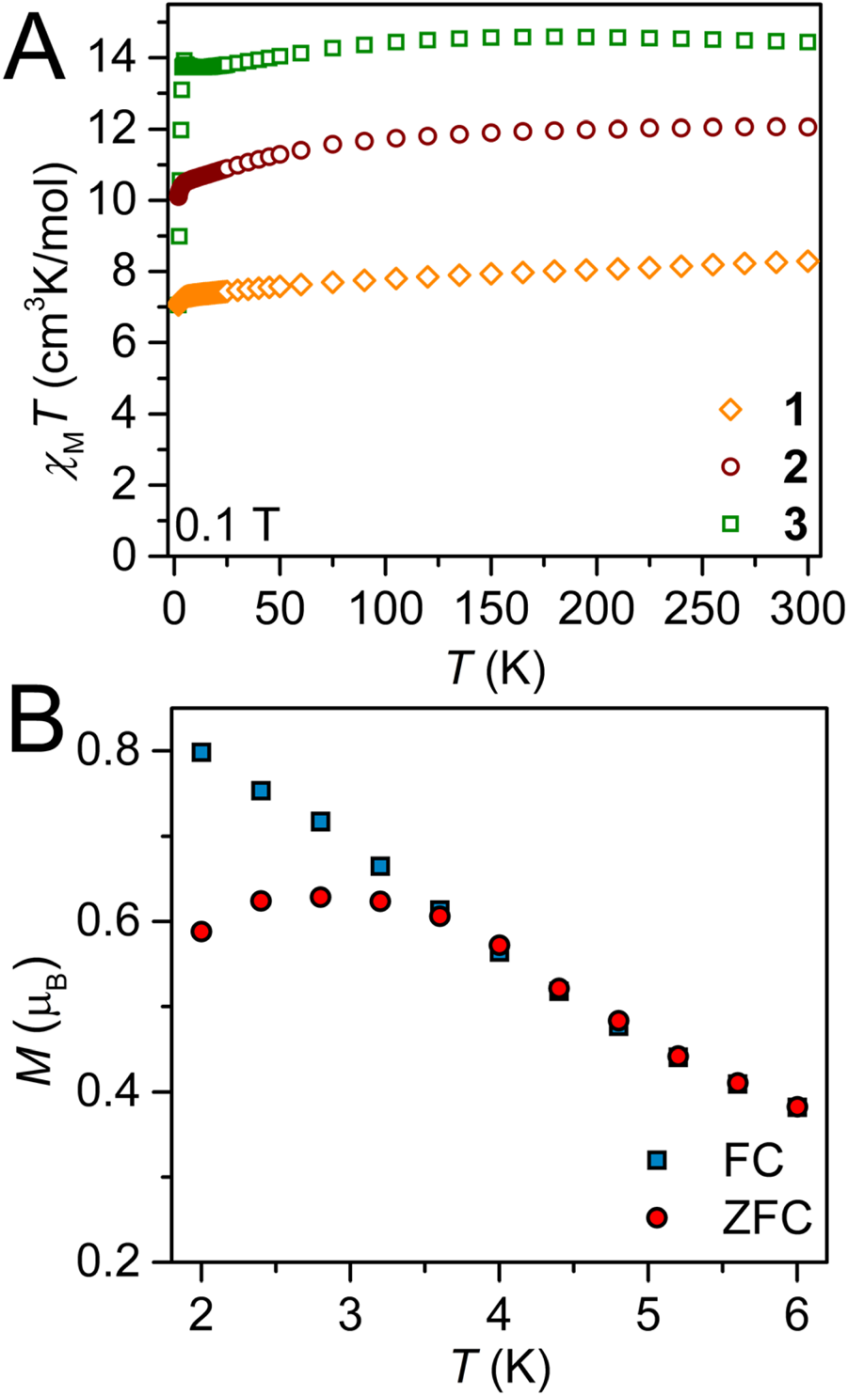
(A) Variable-temperature dc magnetic susceptibility data for 
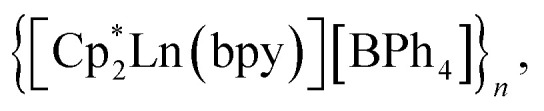
 where Ln = Gd, 1 (orange diamonds), Tb, 2 (maroon circles), Dy, 3 (green squares), collected under a 0.1 T applied dc field. (B) Plot of magnetisation *vs.* temperature for 3 during field-cooled (blue squares) and zero-field-cooled (red circles) measurements depicting the thermoremanent magnetization. The divergence at 4 K is indicative of magnetic blocking.

To investigate the presence of intra- or interchain magnetic exchange coupling in 1–3, temperature-dependent molar magnetic susceptibility (*χ*_M_*T*) data were collected on polycrystalline samples of 1–3 between 2 and 300 K under 0.1 T, 0.5 T, and 1.0 T applied dc fields (Fig. 3A and S31–S35). Under a 0.1 T applied dc field, the room-temperature *χ*_M_*T* values of 8.29, 12.06, and 14.08 cm^3^ K mol^−1^ are consistent with the presence of uncoupled Ln^III^ ions (Gd^III^: 4f^7 8^S_7/2_, *S* = 7/2, *L* = 0, *J* = 7/2, *g* = 2, *χ*_M_*T*_calc_ = 7.88 cm^3^ K mol^−1^, Tb^III^: 4f^8 7^F_6_, *S* = 3/2, *L* = 3, *J* = 6, *g* = 3/2, *χ*_M_*T*_calc_ = 11.82 cm^3^ K mol^−1^; Dy^III^: 4f^9^, ^6^H_15/2_, *S* = 5/2, *L* = 5, *J* = 15/2, *g* = 4/3, *χ*_M_*T*_calc_ = 14.17 cm^3^ K mol^−1^) (Fig. 3A). As the dc field increases, the room temperature *χ*_M_*T* values decrease to 7.74, 11.85, and 13.79 cm^3^ K mol^−1^ at 0.5 T for 1–3, respectively, and yield values of 7.65, 11.92, and 13.75 cm^3^ K mol^−1^ at 1.0 T for 1–3, respectively (Fig. S34 and S35). Notably, as the temperature is lowered under a 0.1 T field, the *χ*_M_*T* values for 3 remain largely unchanged between 300 K and 105 K, and gradually decline to 12.81 cm^3^ K mol^−1^ at 11 K. At lower temperatures, the *χ*_M_*T* values begin to rise, peaking at 12.98 cm^3^ K mol^−1^ at 4.8 K, before precipitously falling to 6.57 cm^3^ K mol^−1^ at 2 K. This significant change in the product of molar magnetic susceptibility and temperature is an indication for magnetic blocking due the pinning of the magnetic moment along the easy axis. The phenomenon of magnetic blocking was further confirmed through the divergence of field- and zero-field-cooled magnetic susceptibility data at 4 K (Fig. 3B). By contrast, the *χ*_M_*T* values monitored for 1 and 2 steadily decline with decreasing temperature reaching 7.28 and 10.50 cm^3^ K mol^−1^ at 5 K, before dropping to 7.06 and 10.15 cm^3^ K mol^−1^ at 2 K for 1 and 2, respectively. In sum, 1–3 feature essentially uncoupled lanthanide ions which is ascribed to the contracted nature of the Ln^III^ valence 4f-orbitals that mitigates effective magnetic exchange coupling when the metals are connected *via* closed-shell bridging ligands.^109^ However, pronounced superexchange between lanthanide ions has been realised through exploiting the diffuse valence orbitals innate to heavy p-block elements such as Bi.^43,44^ The intrachain distances between adjacent paramagnetic centres exceed 12 Å and thus, preclude dipolar magnetic exchange coupling between Ln^III^ ions.

Field-dependent magnetisation measurements were conducted between 2 and 10 K from 0–7 T (Fig. S39–S44). At 2 K, the nonlinear magnetisation curve monitored for 3 is another indication of magnetic blocking (Fig. S43). Furthermore, the highly anisotropic nature of 3 is also validated by the non-superimposable field-dependent reduced magnetisation curves (Fig. S44).

**Fig. 4:**
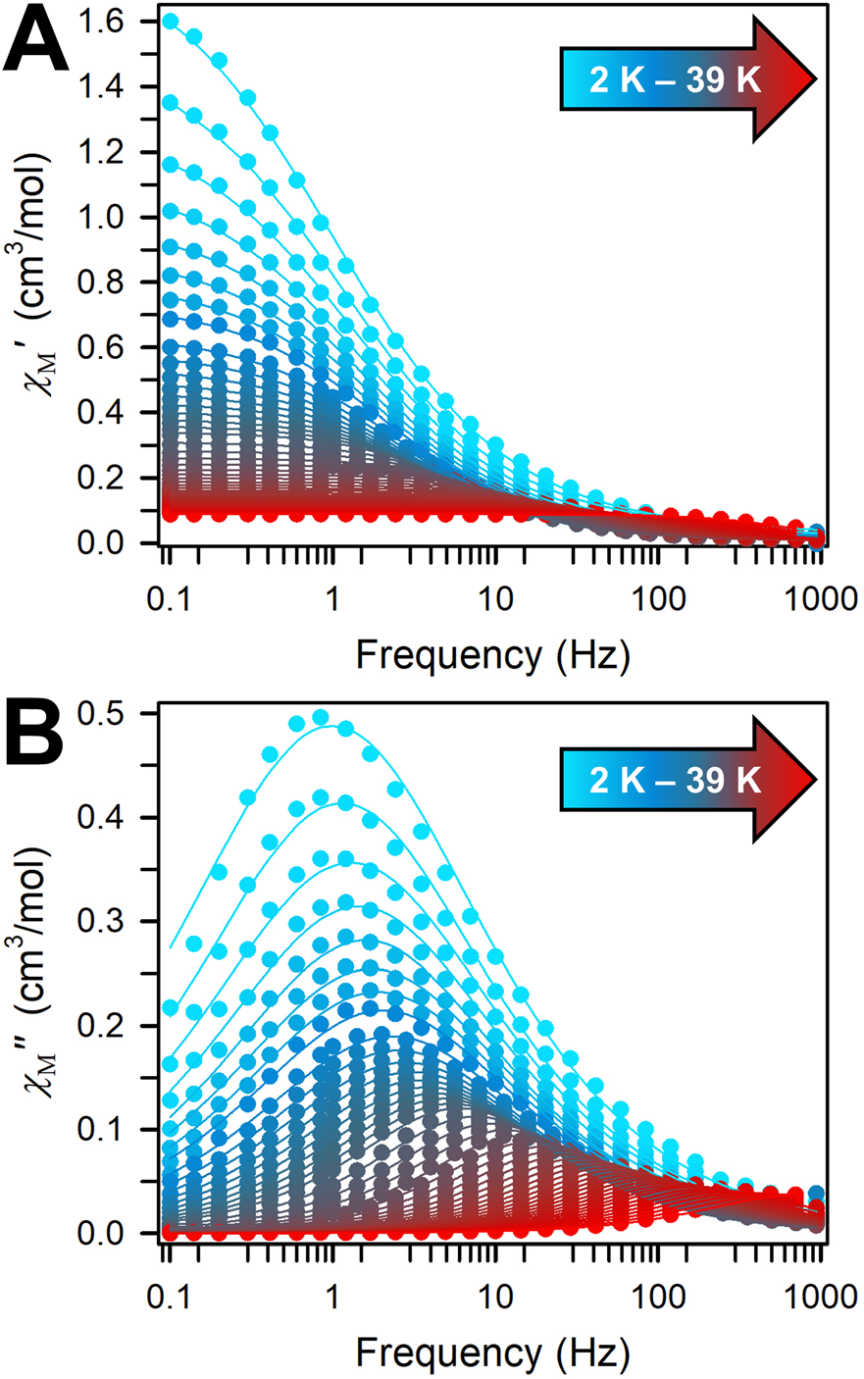
Variable-temperature, variable-frequency in-phase (*χ*_M_*′*) (top) and out-of-phase (*χ*_M_*′′*) (bottom) ac magnetic susceptibility data collected under a zero applied dc field for 
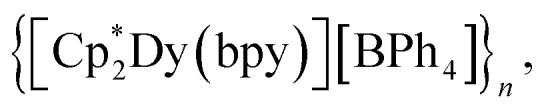
3, from 2 to 39 K. Solid lines indicate the fits to a generalized Debye model.

To elucidate the dynamic magnetic properties of 2 and 3, variable-frequency, variable-temperature in-phase (*χ*_M_*′*) and out-of-phase (*χ*_M_*′′*) ac magnetic susceptibility data were collected for 2 and 3. Indeed, out-of-phase (*χ*_M_*′′*) ac magnetic susceptibility signals emerge from 2 to 39 K under a zero applied dc field, verifying that 3 exhibits slow magnetic relaxation (Fig. 4). At temperatures between 2 and 4.8 K, the out-of-phase (*χ*_M_*′′*) maximum occurs at 1.7 Hz which decreases in intensity with raising temperature but remains invariant with regard to frequency. This temperature-independent behaviour at lower frequencies is an indication of quantum tunnelling of the magnetisation (QTM). At temperatures above 4.8 K, the out-of-phase (*χ*_M_*′′*) maximum shifts towards higher frequencies, until it moves beyond the frequency limit of the magnetometer. This temperature-dependence alludes to the presence of a thermally activated relaxation process.

By contrast, no out-of-phase signals were observed for 2 at 2 K under a zero applied dc field. Under high fields, only a broad out-of-phase (*χ*_M_*′′*) peak with a maximum at high frequencies was monitored, which precluded a thorough investigation of the temperature dependence of the magnetic relaxation in 2.

**Fig. 5:**
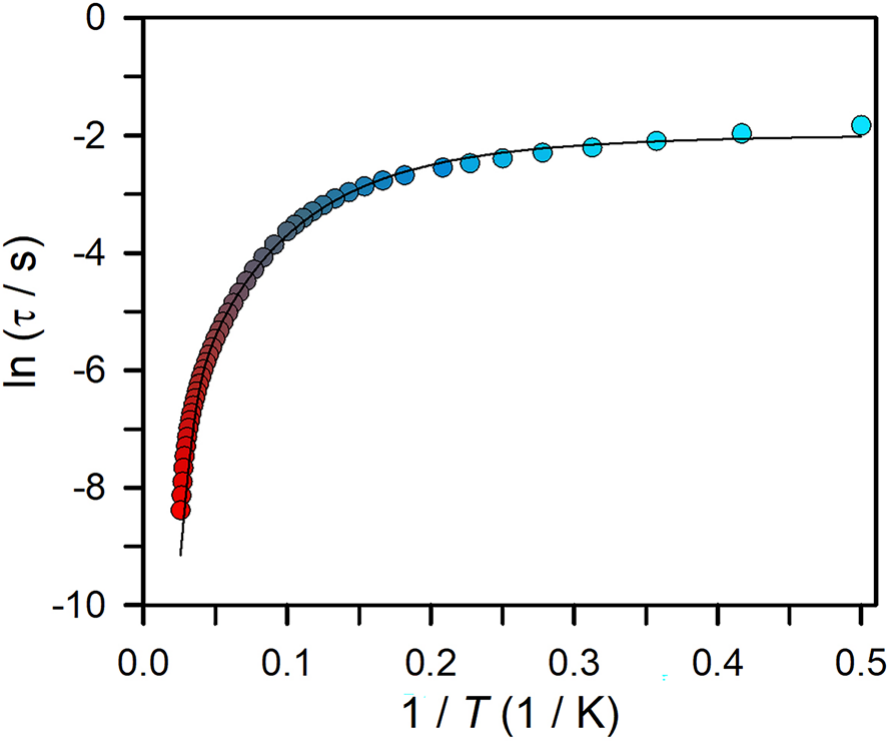
Plot of natural log of the relaxation time *versus* the inverse temperature for 
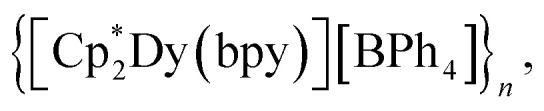
3 (temperature range 2 to 39 K). The black line represents a fit to a Orbach relaxation process, a Raman relaxation process, and a quantum tunneling pathway, as described in the main text, affording *U*_eff_ = 218.6 cm^−1^ and *τ*_0_ = 3.88 × 10^−8^ s. Individual contributions to these fits are shown in Fig. S38.

For 3, the magnetic relaxation times, *τ*, were extracted from fitting Cole–Cole plots employing a generalised Debye model (Fig. S36 and 37) and were applied to generate an Arrhenius plot (Fig. 5). This plot features the temperature dependence of *τ* which reveals detailed insight into the types of magnetic relaxation pathways operational in 3. For an activation barrier to spin-reversal to occur, the system must exchange energy with the lattice (as phonons) to rise to the top of the barrier before relaxation can set in. This preferred mechanism of spin relaxation, the so-called thermally activated Orbach relaxation process, exhibits an exponential dependence of *τ* upon temperature. By contrast, a quantum tunnelling process shows temperature independent *τ*.

The extracted *τ* values exhibit a pronounced curvature, suggestive of the magnetic moments in 3 having access to several spin relaxation pathways which elicit differing temperature dependences to the relaxation times. Thus, a satisfactory fit of the Arrhenius plot required multiple relaxation processes to be taken into account, ultimately leading to an accurate value for the relaxation barrier. The best description of *τ* necessitated the inclusion of a QTM mechanism, a Raman relaxation mechanism, and an Orbach relaxation mechanism, according to *τ*^−1^ = *τ*_QTM_^−1^ + *CT*^*n*^ + *τ*_0_^−1^ exp(−*U*_eff_/*k*_B_*T*), where *U*_eff_ denotes the effective spin-reversal barrier, and *τ*_0_ the pre-exponential factor. The best fit yielded a *U*_eff_ value of 218.6 cm^−1^ and a *τ*_0_ of 3.88 × 10^−8^ s.

**Fig. 6:**
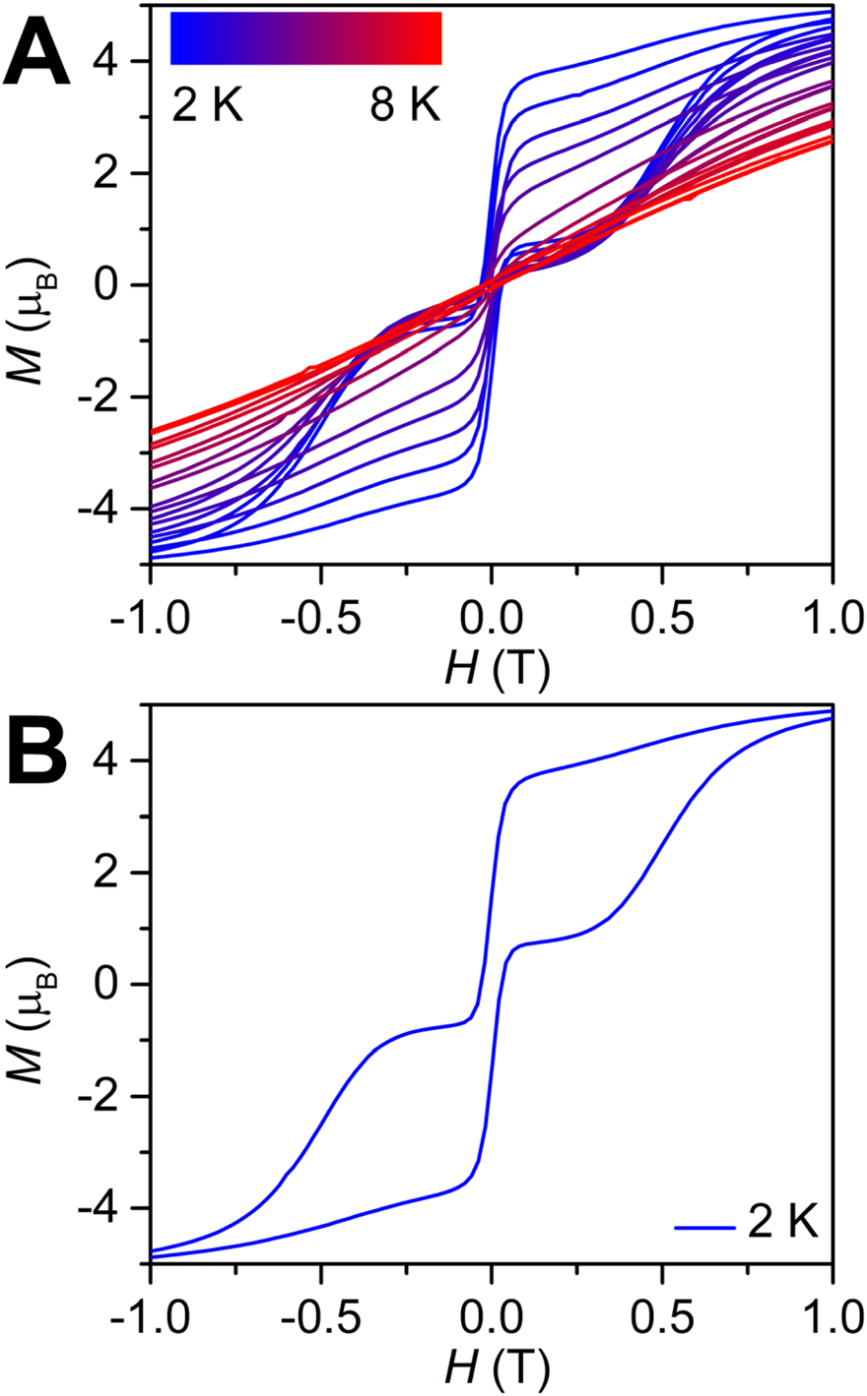
Variable-field magnetisation (*M*) data for 
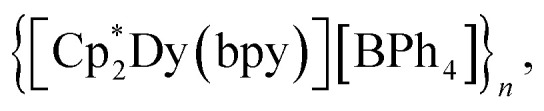
3, collected from 2 K (blue) to 8 K (red) at an average sweep rate of 0.01 T s^−1^ (A). Variable-field magnetisation data for 3 shown only at 2 K (blue) (B).

The prospects of SMMs in high-density information storage devices can be benchmarked through long relaxation times and high blocking temperatures, ideally leading to the observation of open magnetic hysteresis loops corresponding to the ability to retain magnetisation in the absence of an applied dc magnetic field. Here, the opening of the hysteresis is expressed as coercive field, *H*_c_, which equates to the reverse field required to drive the net magnetisation back to zero after having reached magnetic saturation. Indeed, 3 shows magnetic hysteresis loops in response to variable-field magnetisation measurements which were performed between +7 and −7 T, from 2 to 8 K, with an average sweep rate of 100 Oe s^−1^ (Fig. 6, S46 and S47). The measured hysteresis loops for 3, depicted between +1 and −1 T, are open at zero field at temperatures below 8 K, with a maximum *H*_c_ of 346 Oe and a remanent magnetisation *M*_R_ of 1.57*μ*_B_ at 2 K. Here, the variable-field magnetisation gradually falls from high to low fields before precipitously declining close to *H* = 0 Oe. This progression is distinct from a classical butterfly-shape in that the hysteresis loops stay open below 8 K considering the sweep rate employed. Nonetheless, the steep drop in magnetisation originates from rapid relaxation pathways operative in the system such as QTM. This is consistent with the ac magnetic susceptibility data collected in the same temperature regime under zero dc field, albeit taken on a different timescale.

By contrast, the Tb complex 2, exhibits superimposable curves between +7 to −7 T at 1.8 K (Fig. S45). This is in accordance with the rapid magnetic relaxation observed for 2 on the timescale of ac magnetic susceptibility measurements, where no SMM behaviour was detected under zero applied dc field.

Intriguingly, the blocking temperature (*T*_B_) of 8 K exceeds those of the dysprosium-based metallocenium complexes, 
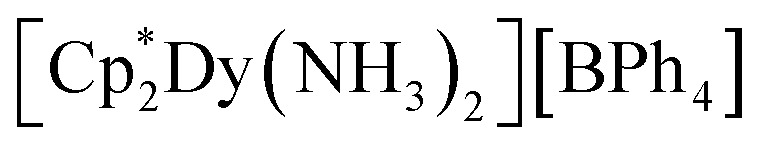
 (5.8 K) and 
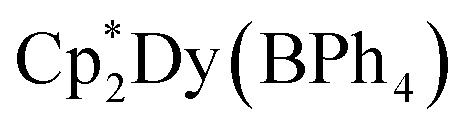
 (5.3 K), yet 3 exhibits a markedly lower energy barrier to spin-reversal than 327 and 95 cm^−1^, respectively, observed in the reported examples. In general, the presence of neutral equatorial ligands is expected to engender a weaker transverse anisotropy with respect to anionic ligands, as observed for instance for 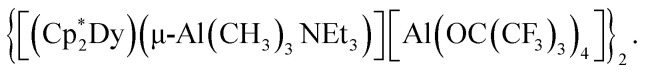
^110^ This idea, however, is complicated by the smaller *U*_eff_ value experimentally determined for 3 with respect to the parent 
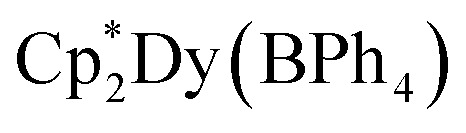
 complex. This trend was also monitored in solvent adducts of heteroleptic dysprosocenium complexes.^111,112^ The larger energy barrier for the di(ammonia) Dy complex can be rationalised through the greater axiality observed in the 
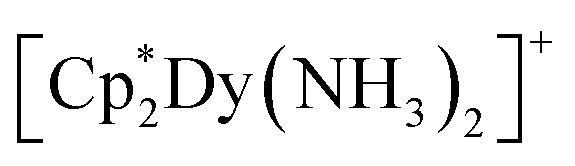
 motif compared to both 3 and 
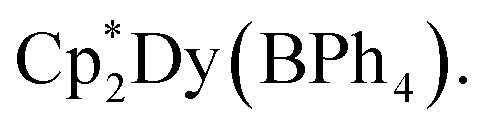
 An alternative explanation to the complex magnetic behaviour of 3 may be found in the context of the neutral N-donor ligands employed. The higher blocking temperature monitored for 3 may result from the coordination of two, long, diamagnetic bridging ligands. The presence of a 4,4′-bipyridine bridge gives rise to a large Dy⋯Dy separation of 12.063(1) Å. This in turn mitigates greater contribution from weak dipolar interactions that enable the onset of rapid relaxation mechanisms such as QTM.^113^ In fact, the shortest Dy⋯Dy distance in 3 is nearly 125% longer than that in 
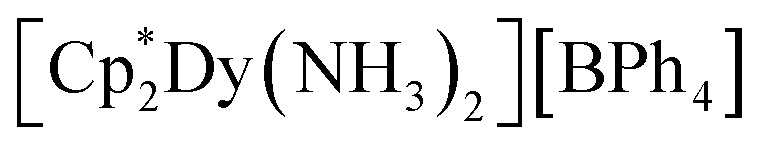
 (9.597(1) Å) and 
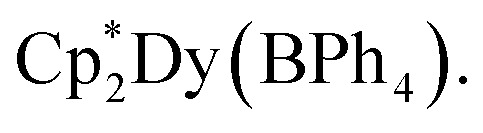
 (9.902(1) Å) (Tables S3 and S4). Importantly, the nature and number of equatorial ligands can also influence the magnetic properties of dysprosium-based SMMs.^108,112,114,115^ More specifically, the enhanced electron donation of bpy with respect to NH_3_ may engender greater *M*_J_ state mixing, undercutting the barrier to spin-reversal in 3. In addition, the weakly coordinated halobenzene adducts of 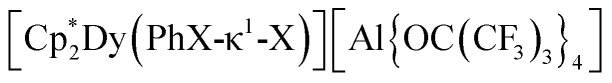 (where X = F, Cl) and 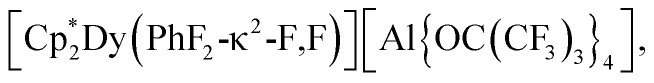 exhibit high blocking temperatures and large *U*_eff_ values. However the haloarene complexes feature significantly larger bite angles, which is known to enhance both the magnetic anisotropy and the resulting spin relaxation barrier of dysprosocenium complexes.^108,112,114^

### Computational insights

**Fig. 7:**
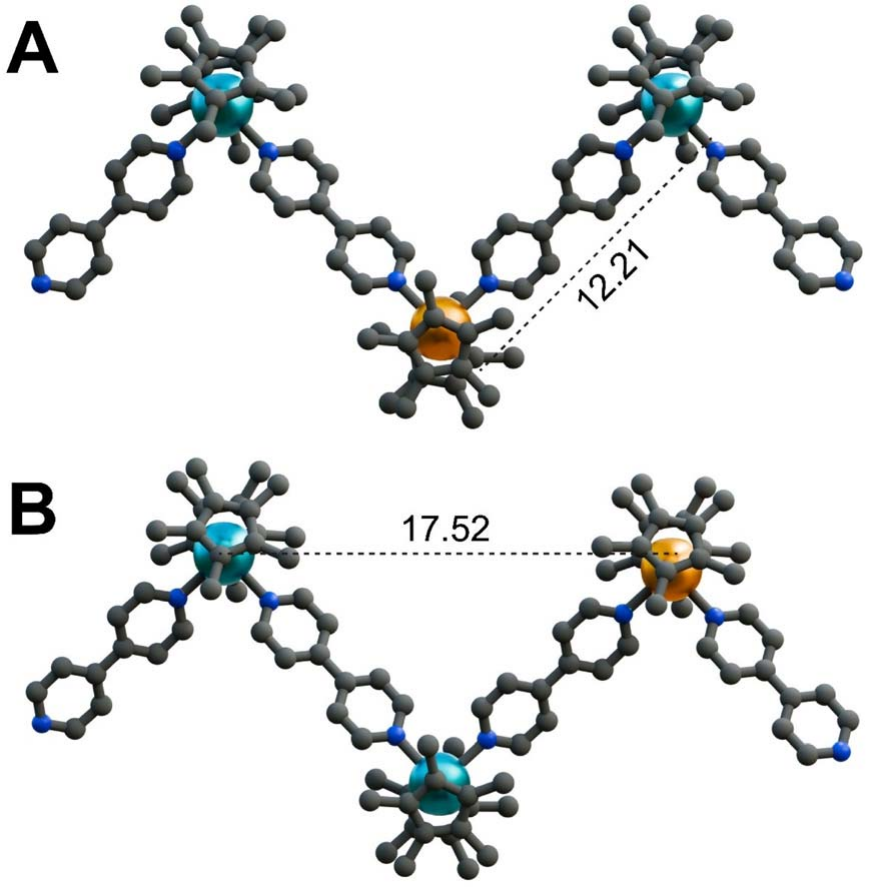
The arrangement of three Gd^III^ centres within the chain structure of 
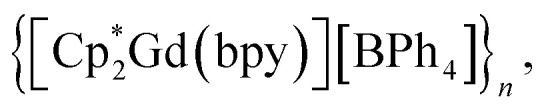
1. (A) Calculated spin density of a Gd^III^ centre in 1, in an antiferromagnetic configuration along the bpy ligands, separated by a distance of 12.21 Å. (B) Calculated spin density of a peripheral Gd^III^ centre in 1, in an antiferromagnetic configuration between non-bridging Gd^III^ ions separated by a distance of 17.52 Å. The orange, blue, and grey spheres represent Gd, N, and C atoms, respectively. Teal and orange surfaces represent the calculated spin densities. H atoms, solvent molecules in the crystal lattice, and the [BPh_4_]^−^ counteranions have been omitted for clarity. The depiction of spin density surfaces precludes the visibility of the Gd^III^ ions. Distances are given in Å.

The polymeric, zigzag-shaped structures of 1–3 contain triangular repeating units, which may usher in antiferromagnetic (AFM) interactions between adjacent Ln^III^ ions or a network of spin frustrated ions.^116^ To probe whether such magnetic phenomena are at play, magnetic exchange pathways between Gd^III^ in 1 were examined through broken-symmetry density functional theory (DFT). Within the crystal structure, two potential intrachain interactions may exist between adjacent Gd^III^ centres. Here, the first pathway corresponds to the interaction along the direction of the bpy ligands, where the metal centres are separated by 12.21 Å (Fig. 7A). The second pathway is along the *c* axis of the crystallographic lattice, translating into a 17.52 Å distance between Gd^III^ ions (Fig. 7B). To determine the most favourable spin density arrangement, these two spin systems were implemented with the spins oriented in an AFM fashion along each pathway. The computationally-derived energy values for these two systems hint at the AFM arrangement of spins along the propagated chain direction being energetically more favourable by 0.604 cm^−1^, Fig. 7A.

To assess the relative magnitudes of the potential exchange coupling constant (*J*) values across the two pathways, two systems were probed where: (a) one of the peripheral Gd^III^ centres was replaced by an Y^III^ ion and (b) the middle Gd^III^ centre was replaced by an Y^III^ centre (Fig. 7, S48 and S49). The spin of one Gd^III^ centre was flipped in these model systems and the *J* values were obtained through the equation *J* = −(*E*_HS_ − *E*_BS_)/(*S*_HS_^2^ − *S*_BS_^2^), where *E*_HS_ and *E*_BS_ are the energies of the high spin and the broken symmetry states, respectively, and *S*_HS_^2^ and *S*_BS_^2^ correspond to the total spin angular momenta for the high spin and broken symmetry states.^117^ This yields a small *J* value of −0.0123 cm^−1^ along the Gd^III^ ions separated by 12.21 Å; whereas no interaction was monitored computationally between Gd^III^ centres along the crystallographic *c* axis, additionally supporting that the AFM spin arrangement along the bpy ligands would be more energetically favourable. This predicted *J* value is in agreement with Gd–Gd exchange coupling monitored for typical dinuclear gadolinium complexes featuring diamagnetic ligands (|*J*| < 0.1 cm^−1^).^109^

To attain a comprehensive picture of potential exchange coupling pathways, an additional interchain interaction between Gd^III^ ions of neighbouring layers was considered. Within the crystal structure of 1, the two closest Gd^III^ centres are 9.099(1) Å apart, which is shorter than the smallest intrachain distance between Gd^III^ ions along the bpy ligands (Fig. S49). Thus, broken-symmetry DFT calculations were performed taking into account two 
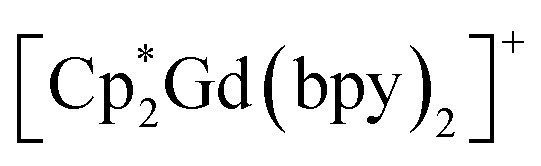
 fragments, and the computed interlayer interaction was energetically favourable by 0.144 cm^−1^ for a ferromagnetic (FM) exchange coupling of 0.0082 cm^−1^ compared to an AFM interaction. Importantly, this is smaller than the coupling constant along the chain regardless of the higher proximity between the Gd^III^ centres, implying the interaction of the Gd^III^ with bpy and Cp* imposes a preference for coupling along the direction of bpy. However, all these potential coupling pathways are predicted to have negligible exchange coupling constants. Thus, for subsequent *ab initio* calculations, it is warranted to consider that the magnetic relaxation of this system is arising solely from single-ion effects.

These results are comparable to the bpy-containing dinuclear Gd^III^ complex, [(Gd(thd)_3_)_2_(μ-bpy)] (Hthd = 2,2,6,6-tetramethyl-3,5-heptanedione), which exhibits weak ferromagnetic coupling (*J* = 0.0491 cm^−1^). The presence of a diamagnetic bridging bpy ligand precludes strong magnetic exchange between the two Gd^III^ centres.^118^ Similarly, 
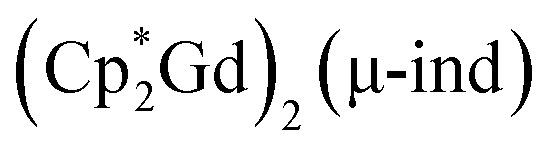
 (ind = indigo) features a diamagnetic bridging ligand between Gd^III^ centres, ultimately affording a weak AFM exchange coupling, *J* = −0.013 cm^−1^.^119^ Furthermore, an even weaker AFM coupling with a *J* of −0.0053(4) cm^−1^ has been observed in a dinuclear gadolinium metallocene complex, 
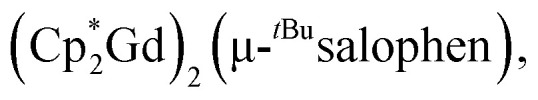
 comprising a large diamagnetic salophen bridge.^120^ This implies that the *J* values obtained through DFT calculations are validated in magnitude and propose potential exchange coupling pathways for 1 that are vanishingly small for experimental determination.

### *Ab initio* calculations

The slow magnetic relaxation in 3 could in principle arise from collective single chain magnet (SCM) behaviour or single-molecule magnet (SMM) behaviour stemming from single-ion effects enabled through isolated Dy^III^ centres. In the case of SCM behaviour, two potential magnetic exchange pathways may contribute to slow magnetic relaxation, namely ferromagnetic intrachain magnetic exchange coupling or interchain dipolar coupling. The first examples of Ln-based SCMs [Ln(hfac)_3_(NITPhOPh)], (where Ln = Eu, Gd, Tb, Dy, Ho, Er, Yb; hfac = hexafluoroacetylacetonate; NITPhOPh = 2,4′-benzoxo-4,4,5,5-tetramethylimidazoline-1-oxyl-3-oxide) contain nitronyl-nitroxide radicals leading to slow magnetic relaxation *via* Glauber mechanism for the Dy analogue, where the room temperature *χ*_M_*T* values matched a single Ln^III^ ion and a *S* = ½ organic radical.^121,122^ By contrast, the *χ*_M_*T* values of 1–3 at 300 K are close to the expected ones for isolated Ln^III^ ions. Notably, the diamagnetic bpy bridges yield negligible magnetic coupling and preclude a collective Glauber relaxation mechanism. Thus, the origin of magnetic relaxation in 3 most likely arises from single-ion effect due to the presence of essentially uncoupled, highly anisotropic Dy ions. Indeed, broken-symmetry DFT calculations on 1 suggest extremely weak, essentially non-existent, coupling between adjacent Gd^III^ sites. Furthermore, the coupling between nearest chains decreases by one order of magnitude. This effectively eliminates ferromagnetic intrachain magnetic exchange and interchain dipolar exchange coupling mechanisms as the origins of the magnetic properties observed in 3.

**Fig. 8:**
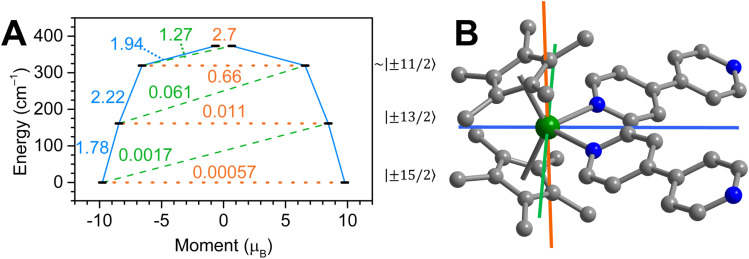
(A) Estimated relaxation barrier comprising the four lowest-lying Kramers doublets with relaxation pathways shown for 
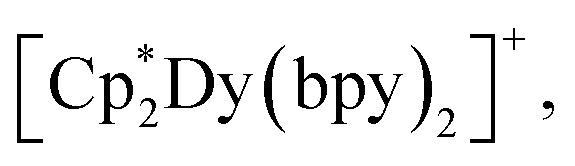
3′. Solid blue and dashed green lines indicate Orbach relaxation processes, orange dotted lines indicate quantum tunnelling of the magnetisation (QTM)/thermally activated QTM pathways. Values next to the arrows correspond to the respective transition magnetic moment matrix elements. The numbers on the right are given for the primary *M*_J_ state comprising the wave function for each state. (B) Plot of the *g*-tensor components calculated for the |±15/2〉 ground state Kramers doublet of 3′. Colour code: *g*_*x*_ (green), *g*_*y*_ (blue) and *g*_*z*_ (orange) with respective compositions 0.0007 (*g*_*x*_), 0.0027 (*g*_*y*_) and 19.5595 (*g*_*z*_).

To ascertain the origin of SMM behaviour, we conducted complete active space self-consistent field (CASSCF) calculations on a model of the repeating unit in 3, namely the 
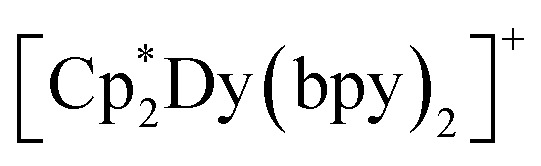
 fragment, 3′ (Fig. 8). CASSCF/NEVPT2/QDPT calculations were carried out using the Orca 5.0.4 program suite on crystal coordinates, optimised for the hydrogen positions. The low-lying energy spectrum of 3′ was calculated considering the first eight Kramers doublets (KD), and transition dipole moments (TDM) connecting these states were used to obtain an estimate for the relaxation barrier of a single repeating unit within 3.

The ground state KD1 anisotropy of the Dy^III^ ion in 3′ is strictly axial and determined by the bis(Cp*) ligand scaffold comprising essentially pure *M*_J_ = |±15/2〉 state and *g*_*z*_ = 19.56 with negligible *g*_*x*_ and *g*_*y*_ components (Fig. 8). Consequently, the TDM connecting these states is considerably smaller than the TDM connecting with the first excited KD2. The first KD2 lies 161 cm^−1^ above the ground KD1 and is of pure *M*_J_ = |±13/2〉 character, with the *g* tensor still being dominated by the *g*_*z*_ component (16.92) but noticeable *g*_*x*_/*g*_*y*_ = 0.03 contributions. The TDM connecting |−13/2〉 and |+13/2〉 states is two orders of magnitude larger than the one connecting KD1, but still small enough for the KD2 → KD3 transition to be more likely. Admixture of excited states becomes substantial for the second excited KD3 at 320 cm^−1^, where the dominant *M*_J_ = |±11/2〉 states are blended with higher-lying excited states such as |±9/2〉 and |±3/2〉, giving rise to non-negligible transverse *g*_*x*_ = 1.51 and *g*_*y*_ = 2.39 contributions, rivalling the *g*_*z*_ = 13.42 primary component. The TDM of KD3 is large enough to shortcut the relaxation barrier at ∼0.7, marking the high end of the estimated relaxation barrier for 3′.

The estimated barrier height of 161–320 cm^−1^ compares reasonably well to the experimentally obtained barrier of 218.6 cm^−1^ under a zero Oe applied dc field, given that vibrational effects were not accounted for in this model, and Raman/QTM processes were not suppressed to yield *U*_eff_.

### EPR spectroscopy

**Fig. 9:**
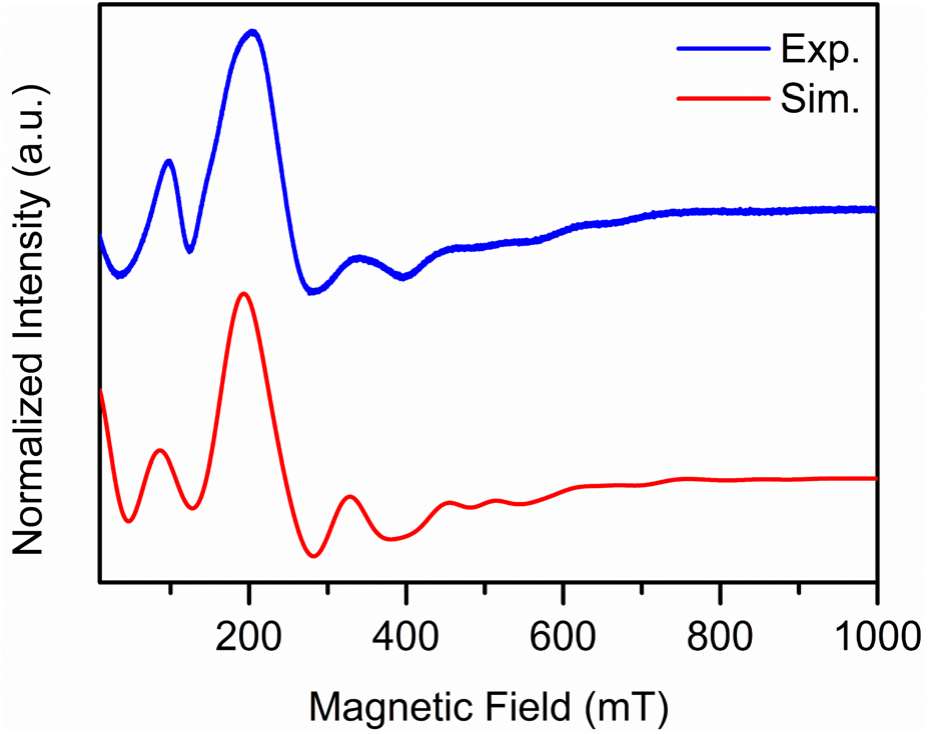
Experimental (blue) and simulated (red) cw-EPR spectrum of 
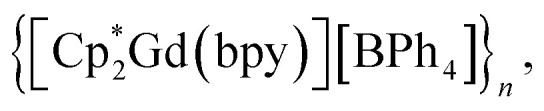
1, at 110 K collected on finely ground crystalline solids. The experimental spectrum was simulated using the parameters: *S* = 7/2, *g* = 2.0871, *D* = 0.09186 cm^−1^, *E* = 0.02269 cm^−1^ and lwpp = 48.

Continuous-wave EPR spectroscopy at X-band frequency was employed to probe the electronic structure of 1. The measurement was conducted on finely ground crystalline solids, and the obtained spectrum spans almost over a 1 T region with broad features (Fig. 9). The spin Hamiltonian of such a system with spin multiplicity larger than half (*S* > 1/2) can be described by eqn (2).^123,124^

2 *H*_S_ = *gβSB* + *D*[*S*_*z*_^2^ − *S*(*S* + 1)/3 + (*E*/*D*)(*S*_*x*_^2^ − *S*_*y*_^2^)]

Here, *g* is the dimensionless *g*-value, *β* is the Bohr magneton, *B* is the magnitude of the applied magnetic field, *D* is the axial zero-field splitting (ZFS) parameter and *E* is the rhombic ZFS parameter. The *E*/*D* value ranges from 0 to 1/3 depending on if the system is fully axial or fully rhombic. Understanding these terms enables unveiling electronic structure properties of the spin system.

Therefore, the experimental spectrum was simulated using the ‘pepper’ module of EasySpin. Initially, the first derivative spectrum of the collected data was used to determine the *g*-value of 2.0871 for the simulation. Then, the genetic algorithm of the esfit function was employed for the determination of *D* and *E*/*D* terms. Fitting the data, produced *D* and *E* values of 0.09186 cm^−1^ and 0.02269 cm^−1^, respectively. This leads to an *E*/*D* ratio of 0.247 indicating that the spin system is more rhombic in nature and agrees with the lower symmetry seen around the Gd centre in the crystal structure.^125^ Furthermore, the spin system simulation was carried out assuming no coupling between the metal centres as evidenced by magnetic measurements and computational analyses. To validate the accuracy of the EPR simulation, the first integral of the EPR spectrum was also simulated using the same parameters (Fig. S50).

This assessment bears resemblance to the X-band cw-EPR spectrum of the mononuclear complex, [Gd(H_2_O)(phen)_2_(NCS)_3_]·phen·0.5H_2_O (phen = 1,10-phenanthroline) collected on a polycrystalline sample. This low symmetry molecule has ZFS parameters of *D* = 0.092 cm^−1^ and *E* = 0.0089 cm^−1^, and exhibits a more axial nature owing to its bicapped-triangular prism geometry around the metal centre.^125^ Another comparable system constitutes the dinuclear quinone radical-bridged Gd complex {Gd[(QMe_4_)˙^−^Cl_2_(THF)_3_]}_2_ (where QMe_4_ = tetramethylquinone) where the two Gd^III^ centres show very weak antiferromagnetic coupling (*J*_Gd–Gd_ = −0.004 cm^−1^). This seven-coordinate system exhibits *D* = 0.122 cm^−1^ and *E* = 0.0327 cm^−1^, affording a more analogous *E*/*D* ratio of 0.268, indicative of a rhombic system.^126^ Moreover, the eight-coordinate mononuclear Gd complex 
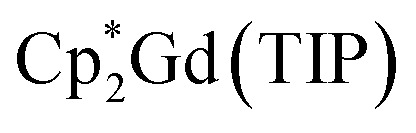
 (where TIP = tetraiodopyrrole), featuring a similar coordination geometry to 1, possesses the ZFS parameters *D* = −0.122 cm^−1^ and |*E*| = 0.037 cm^−1^ (|*E*/*D*| = 0.304), further suggesting that the rhombic assignment is suitable for such low symmetry systems.^127^ These comparisons demonstrate that describing 1 as an uncoupled, mononuclear system is accurate for the description of its electronic structure.

## Conclusions

The first organometallic lanthanide metallocene 1D networks containing organic bridging ligands were crystallised and unambiguously characterised. Single-crystal X-ray diffraction analysis confirmed the one-dimensional nature of the lanthanide-based chain compounds with the formula 
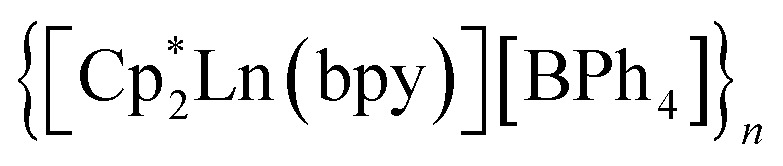
 (Ln = Gd (1), Tb (2), Dy (3)). The bipyridyl ligands are bound to the metals equatorially and act as bridges between adjacent metallocenium 
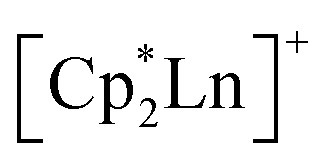
 units, giving rise to intricate zigzag layers, with the charge-balancing (BPh_4_)^−^ anions residing between the bipyridyl ligands. The developed synthetic route to such unprecedented organometallic polymers has potential to be highly tuneable, paving the synthetic way to various crystalline metallopolymers composed of [Cp^R^_2_Ln]^+^ and an organic linker. Pertaining to magnetism, the devised synthesis corresponds to a new approach to multinuclear one-dimensional systems bearing highly anisotropic ions. The substitution of Ln^III^ ions with redox-active actinides may lead to intriguing magnetic materials.

Notably, the dysprosium congener, 3, is a zero-field single-molecule magnet and shows slow magnetic relaxation under a zero dc field with an effective spin-relaxation barrier of *U*_eff_ = 218.6 cm^−1^. Excitingly, 3 exhibits open magnetic hysteresis loops at temperatures below 8 K corresponding to real magnetic memory effect. The origin of single-molecule magnetism in 3 stems solely from the high magnetic anisotropy of the single Dy^III^ ions and the crystal field, rather than a strongly exchange-coupled system. This is also corroborated by the *ab initio* calculations, which predict strong axiality in the ground state imposed by the bis(Cp*) framework. The predicted relaxation barrier arising from Dy^III^ ion single-ion effect is in accordance with the experimental data, further highlighting the negligible magnetic coupling between neighbouring Ln ions. Cw-EPR spectroscopic analysis conducted on a solid sample of 1 reflects the vanishing magnetic coupling as observed by magnetic measurements and computational predictions. Furthermore, the derived rhombic nature of the electronic distribution around the metal centres *via* EPR spectroscopy is in agreement with the low symmetry exhibited by these compounds. Intriguingly, the presence of redox-active bridging ligands, such as 4,4′-bipyridine, offers a promising avenue for future exploration, particularly through chemical reduction or oxidation to access compounds containing an open-shell unit. Such systems may exhibit strong metal–radical coupling, potentially leading to the formation of giant spin ground states and novel magnetic behaviours. Thus, the strategy for the generation of such unprecedented organometallic lanthanide polymers paves the way to new, highly tuneable magnetic and spintronic materials.

## Author contributions

Ernesto Castellanos prepared and characterised each compound by crystallography, magnetometry, vibrational spectroscopy, and elemental analysis. Florian Benner provided experimental assistance and performed *ab initio* calculations. Saroshan Deshapriya collected EPR data and performed density functional theory calculations. Selvan Demir assisted with data analysis, formulated, and directed the research, and wrote the manuscript with input from all authors.

## Conflicts of interest

There are no conflicts to declare.

## Supplementary Material

SC-016-D5SC05460E-s001

SC-016-D5SC05460E-s002

## Data Availability

All computational data, spectroscopic data, supplementary figures and tables, and detailed crystallographic information can be found in the SI. See DOI: https://doi.org/10.1039/d5sc05460e. Crystallographic data are available *via* the Cambridge Crystallographic Data Centre (CCDC): 2474597 (1), 2474596 (2), and 2474598 (3). The data supporting this article have been included as part of the SI.^128*a*–*c*^

## References

[cit1] Williams K. A., Boydston A. J., Bielawski C. W. (2007). Main-chain organometallic polymers: synthetic strategies, applications, and perspectives. Chem. Soc. Rev..

[cit2] Whittell G. R., Manners I. (2007). Metallopolymers: New Multifunctional Materials. Adv. Mater..

[cit3] Marin V., Holder E., Hoogenboom R., Schubert U. S. (2007). Functional ruthenium(ii)- and iridium(iii)-containing polymers for potential electro-optical applications. Chem. Soc. Rev..

[cit4] Wong W., Harvey P. D. (2010). Recent Progress on the Photonic Properties of Conjugated Organometallic Polymers Built Upon the *trans*-Bis(*para*-ethynylbenzene)bis(phosphine)platinum(ii) Chromophore and Related Derivatives. Macromol. Rapid Commun..

[cit5] Liu F., Abdiryim T., Liu X. (2024). Metallopolymers as functional materials for multiple applications. Polymer.

[cit6] Whittell G. R., Hager M. D., Schubert U. S., Manners I. (2011). Functional soft materials from metallopolymers and metallosupramolecular polymers. Nat. Mater..

[cit7] Caruana D. J., Heller A. (1999). Enzyme-Amplified Amperometric Detection of Hybridization and of a Single Base Pair Mutation in an 18-Base Oligonucleotide on a 7 μm-Diameter Microelectrode. J. Am. Chem. Soc..

[cit8] Gajovic N., Binyamin G., Warsinke A., Scheller F. W., Heller A. (2000). Operation of a Miniature Redox Hydrogel-Based Pyruvate Sensor in Undiluted Deoxygenated Calf Serum. Anal. Chem..

[cit9] Chen R., Feng J., Jeon J., Sheehan T., Rüttiger C., Gallei M., Shukla D., Su X. (2021). Structure and Potential-Dependent Selectivity in Redox-Metallopolymers: Electrochemically Mediated Multicomponent Metal Separations. Adv. Funct. Mater..

[cit10] Greenfield J. L., Di Nuzzo D., Evans E. W., Senanayak S. P., Schott S., Deacon J. T., Peugeot A., Myers W. K., Sirringhaus H., Friend R. H., Nitschke J. R. (2021). Electrically Induced Mixed Valence Increases the Conductivity of Copper Helical Metallopolymers. Adv. Mater..

[cit11] Valério C., Fillaut J.-L., Ruiz J., Guittard J., Blais J.-C., Astruc D. (1997). The Dendritic Effect in Molecular Recognition: Ferrocene Dendrimers and Their Use as Supramolecular Redox Sensors for the Recognition of Small Inorganic Anions. J. Am. Chem. Soc..

[cit12] Astruc D. (1987). How to Design Organometallic “Electron-Reservoirs”. Comments Inorg. Chem..

[cit13] Russell T. P. (2002). Surface-Responsive Materials. Science.

[cit14] Kumar A., Bawa S., Ganorkar K., Kumar Ghosh S., Bandyopadhyay A. (2020). Syntheses, characterization, multi-acid fluorescence sensing and electroluminescence properties of Cr(ii)-based metallopolymers. Polym. Chem..

[cit15] Djeda R., Rapakousiou A., Liang L., Guidolin N., Ruiz J., Astruc D. (2010). Click Syntheses of 1,2,3-Triazolylbiferrocenyl Dendrimers and the Selective Roles of the Inner and Outer Ferrocenyl Groups in the Redox Recognition of ATP^2−^ and Pd^2+^. Angew. Chem., Int. Ed..

[cit16] Péter M., Lammertink R. G. H., Hempenius M. A., Vancso G. J. (2005). Electrochemistry of Surface-Grafted Stimulus-Responsive Monolayers of Poly(ferrocenyldimethylsilane) on Gold. Langmuir.

[cit17] Spokoyny A. M., Kim D., Sumrein A., Mirkin C. A. (2009). Infinite coordination polymer nano- and microparticle structures. Chem. Soc. Rev..

[cit18] Carné A., Carbonell C., Imaz I., Maspoch D. (2010). Nanoscale metal–organic materials. Chem. Soc. Rev..

[cit19] Peng Y., Yang W. (2020). 2D Metal-Organic Framework Materials for Membrane-Based Separation. Adv. Mater. Interfaces.

[cit20] Kitagawa S., Kitaura R., Noro S. (2004). Functional Porous Coordination Polymers. Angew. Chem., Int. Ed..

[cit21] Furukawa H., Cordova K. E., O'Keeffe M., Yaghi O. M. (2013). The Chemistry and Applications of Metal-Organic Frameworks. Science.

[cit22] Stanley J. M., Holliday B. J. (2012). Luminescent lanthanide-containing metallopolymers. Coord. Chem. Rev..

[cit23] Dovzhenko A. P., Vasilyev V. A., Kornev T. A., Chinarev A. A., Nizovtsev A. V., Zairov R. R., Sinyashin O. G., Mustafina A. R. (2024). Bimodal Magneto-Luminescent Response of Lanthanide Metallopolymers for Distinguishing of Phosphates in Aqueous Solutions. Macromol. Chem. Phys..

[cit24] Eggers S. H., Kopf J., Fischer R. D. (1986). On the metal coordination in base-free tris(cyclopentadienyl) complexes of the lanthanoids. 2. The X-ray structure of tris(cyclopentadienyl)lanthanum(iii): a notably stable polymer displaying more than three different lanthanum-carbon interactions. Organometallics.

[cit25] Hinrichs W., Melzer D., Rehwoldt M., Jahn W., Fischer R. D. (1983). Koordinationsverhältnisse in basenfreien tricyclopentadienyl-lanthanoid(iii)-komplexen: I. Röntgenstrukturanalyse von tricyclopentadienyl-praseodym(iii). J. Organomet. Chem..

[cit26] Eggers S. H., Schultze H., Kopf J., Fischer R. D. (1986). Isomorphism of Tris(cyclopentadienyl)lutetium(iii) and Tris(cyclopentadienyl)scandium(iii): An Unexpected Consequence of the Lanthanoid Contraction. Angew. Chem., Int. Ed..

[cit27] Moreno-Pineda E., Wernsdorfer W. (2021). Measuring molecular magnets for quantum technologies. Nat. Rev. Phys..

[cit28] Committee on Identifying Opportunities at the Interface of Chemistry and Quantum Information Science , Board on Chemical Sciences and Technology, Board on Life Sciences, Division on Earth and Life Studies, and National Academies of Sciences, Engineering, and Medicine, Advancing Chemistry and Quantum Information Science: An Assessment of Research Opportunities at the Interface of Chemistry and Quantum Information Science in the United States, National Academies Press, Washington, D.C., 2023

[cit29] Shiddiq M., Komijani D., Duan Y., Gaita-Ariño A., Coronado E., Hill S. (2016). Enhancing coherence in molecular spin qubits *via* atomic clock transitions. Nature.

[cit30] Kundu K., White J. R. K., Moehring S. A., Yu J. M., Ziller J. W., Furche F., Evans W. J., Hill S. (2022). A 9.2 GHz clock transition in a Lu(ii) molecular spin qubit arising from a 3467 MHz hyperfine interaction. Nat. Chem..

[cit31] Sanvito S. (2010). Molecular spintronics: the rise of spinterface science. Nat. Phys..

[cit32] Bogani L., Wernsdorfer W. (2008). Molecular spintronics using single-molecule magnets. Nat. Mater..

[cit33] Goodwin C. A. P., Ortu F., Reta D., Chilton N. F., Mills D. P. (2017). Molecular magnetic hysteresis at 60 kelvin in dysprosocenium. Nature.

[cit34] Guo F.-S., Day B. M., Chen Y.-C., Tong M.-L., Mansikkamäki A., Layfield R. A. (2017). A Dysprosium Metallocene Single-Molecule Magnet Functioning at the Axial Limit. Angew. Chem., Int. Ed..

[cit35] Guo F.-S., Day B. M., Chen Y., Tong M., Mansikkamäki A., Layfield R. A. (2020). Corrigendum: A Dysprosium Metallocene Single-Molecule Magnet Functioning at the Axial Limit. Angew. Chem., Int. Ed..

[cit36] Gould C. A., Randall McClain K., Yu J. M., Groshens T. J., Furche F., Harvey B. G., Long J. R. (2019). Synthesis and Magnetism of Neutral, Linear Metallocene Complexes of Terbium(ii) and Dysprosium(ii). J. Am. Chem. Soc..

[cit37] Demir S., Gonzalez M. I., Darago L. E., Evans W. J., Long J. R. (2017). Giant coercivity and high magnetic blocking temperatures for N_2_^3−^ radical-bridged dilanthanide complexes upon ligand dissociation. Nat. Commun..

[cit38] Guo F. S., Day B. M., Chen Y. C., Tong M. L., Mansikkamäki A., Layfield R. A. (2018). Magnetic hysteresis up to 80 kelvin in a dysprosium metallocene single-molecule magnet. Science.

[cit39] Gould C. A., McClain K. R., Reta D., Kragskow J. G. C., Marchiori D. A., Lachman E., Choi E.-S., Analytis J. G., Britt R. D., Chilton N. F., Harvey B. G., Long J. R. (2022). Ultrahard magnetism from mixed-valence dilanthanide complexes with metal-metal bonding. Science.

[cit40] Benner F., La Droitte L., Cador O., Le Guennic B., Demir S. (2023). Magnetic hysteresis and large coercivity in bisbenzimidazole radical-bridged dilanthanide complexes. Chem. Sci..

[cit41] Benner F., Demir S. (2023). From unprecedented 2,2′-bisimidazole-bridged rare earth organometallics to magnetic hysteresis in the dysprosium congener. Inorg. Chem. Front..

[cit42] Demir S., Jeon I.-R., Long J. R., Harris T. D. (2015). Radical ligand-containing single-molecule magnets. Coord. Chem. Rev..

[cit43] Zhang P., Benner F., Chilton N. F., Demir S. (2022). Organometallic lanthanide bismuth cluster single-molecule magnets. Chem.

[cit44] Zhang P., Nabi R., Staab J. K., Chilton N. F., Demir S. (2023). Taming Super-Reduced Bi_2_^3−^ Radicals with Rare Earth Cations. J. Am. Chem. Soc..

[cit45] Pugh T., Chilton N. F., Layfield R. A. (2017). Antimony-ligated dysprosium single-molecule magnets as catalysts for stibine dehydrocoupling. Chem. Sci..

[cit46] Ruan Z.-Y., Tong M.-L. (2022). Single-molecule magnets bridged by a bismuth Zintl ion. Chem.

[cit47] Gould C. A., Mu E., Vieru V., Darago L. E., Chakarawet K., Gonzalez M. I., Demir S., Long J. R. (2020). Substituent Effects on Exchange Coupling and Magnetic Relaxation in 2,2′-Bipyrimidine Radical-Bridged Dilanthanide Complexes. J. Am. Chem. Soc..

[cit48] Du J., Cobb P. J., Ding J., Mills D. P., Liddle S. T. (2023). f-Element heavy pnictogen chemistry. Chem. Sci..

[cit49] Pugliese E. R., Benner F., Demir S. (2023). Isolation of an organometallic yttrium bismuth cluster and elucidation of its electronic structure. Chem. Commun..

[cit50] Pugliese E. R., Benner F., Demir S. (2023). From an Isolable Bismolyl Anion to an Yttrium–Bismolyl Complex with μ-Bridging Bismuth(I) Centers and Polar Covalent Y-Bi Bonds. Chem.–Eur. J..

[cit51] Delano IV F., Castellanos E., McCracken J., Demir S. (2021). A rare earth metallocene containing a 2,2′-azopyridyl radical anion. Chem. Sci..

[cit52] Delano IV F., Demir S. (2024). Implementation of 2,2′-azobispyridine radical mono- and dianions in dinuclear rare earth metal complexes. Chem. Commun..

[cit53] Benner F., Demir S. (2024). Isolation of Elusive Fluoflavine Radicals in Two Differing Oxidation States. J. Am. Chem. Soc..

[cit54] Gould C. A., Darago L. E., Gonzalez M. I., Demir S., Long J. R. (2017). A Trinuclear Radical-Bridged Lanthanide Single-Molecule Magnet. Angew. Chem., Int. Ed..

[cit55] Konchenko S. N., Pushkarevsky N. A., Gamer M. T., Köppe R., Schnöckel H., Roesky P. W. (2009). [{(η^5^-C_5_Me_5_)_2_Sm}_4_P_8_]: A Molecular Polyphosphide of the Rare-Earth Elements. J. Am. Chem. Soc..

[cit56] Zhang P., Luo Q., Zhu Z., He W., Song N., Lv J., Wang X., Zhai Q., Zheng Y., Tang J. (2023). Radical-Bridged Heterometallic Single-Molecule Magnets Incorporating Four Lanthanoceniums. Angew. Chem., Int. Ed..

[cit57] Zhang J., Cai R., Chen Z., Zhou X. (2007). Facile Construction of Lanthanide Metallomacrocycles with the Bridging Imidazolate and Triazolate Ligands and Their Ring Expansions. Inorg. Chem..

[cit58] Schoo C., Bestgen S., Egeberg A., Seibert J., Konchenko S. N., Feldmann C., Roesky P. W. (2019). Samarium Polyarsenides Derived from Nanoscale Arsenic. Angew. Chem., Int. Ed..

[cit59] Schoo C., Bestgen S., Egeberg A., Klementyeva S., Feldmann C., Konchenko S. N., Roesky P. W. (2018). Samarium Polystibides Derived from Highly Activated Nanoscale Antimony. Angew. Chem., Int. Ed..

[cit60] Bajaj N., Mavragani N., Kitos A. A., Chartrand D., Maris T., Mansikkamäki A., Murugesu M. (2024). Hard single-molecule magnet behavior and strong magnetic coupling in pyrazinyl radical-bridged lanthanide metallocenes. Chem.

[cit61] Mavragani N., Kitos A. A., Gálico D. A., Mansikkamäki A., Murugesu M. (2023). Probing the magnetic and magneto-optical properties of a radical-bridged Tb_4_ single-molecule magnet. Chem. Commun..

[cit62] Mavragani N., Errulat D., Gálico D. A., Kitos A. A., Mansikkamäki A., Murugesu M. (2021). Radical-Bridged Ln_4_ Metallocene Complexes with Strong Magnetic Coupling and a Large Coercive Field. Angew. Chem., Int. Ed..

[cit63] Gupta H., Vincenzini B. D., Bacon A. M., Schelter E. J. (2024). Assembly of a trapped valent Ce^III/IV^–TCNQ complex through metal–ligand redox cooperativity. Chem. Commun..

[cit64] Voigt L., Kubus M., Pedersen K. S. (2020). Chemical engineering of quasicrystal approximants in lanthanide-based coordination solids. Nat. Commun..

[cit65] Ferreira da Rosa P. P., Kitagawa Y., Shoji S., Oyama H., Imaeda K., Nakayama N., Fushimi K., Uekusa H., Ueno K., Goto H., Hasegawa Y. (2022). Preparation of photonic molecular trains *via* soft-crystal polymerization of lanthanide complexes. Nat. Commun..

[cit66] Matthes P. R., Eyley J., Klein J. H., Kuzmanoski A., Lambert C., Feldmann C., Müller-Buschbaum K. (2015). Photoluminescent One-Dimensional Coordination Polymers from Suitable Pyridine Antenna and LnCl_3_ for Visible and Near-IR Emission. Eur. J. Inorg. Chem..

[cit67] Armelao L., Belli Dell'Amico D., Bellucci L., Bottaro G., Labella L., Marchetti F., Samaritani S. (2016). A convenient synthesis of highly luminescent lanthanide 1D-zigzag coordination chains
based only on 4,4′-bipyridine as connector. Polyhedron.

[cit68] Ahmad Bhat S., Ahmad Zargar R., Hasan N., Ali J., Ahmad Mir N., Wankar S., Iftikhar K. (2024). 1D-photosensitized lanthanide polymers with flexible 4,4′-bipyridyl linker. J. Photochem. Photobiol., A.

[cit69] Sorg J. R., Wehner T., Matthes P. R., Sure R., Grimme S., Heine J., Müller-Buschbaum K. (2018). Bismuth as a versatile cation for luminescence in coordination polymers from BiX_3_/4,4′-bipy: understanding of photophysics by quantum chemical calculations and structural parallels to lanthanides. Dalton Trans..

[cit70] Long B. N., Sperling J. M., Windorff C. J., Huffman Z. K., Albrecht-Schönzart T. E. (2023). Expanding Transuranium Organoactinide Chemistry: Synthesis and Characterization of (Cp_3_′M)_2_(μ-4,4-bpy) (M = Ce, Np, Pu). Inorg. Chem..

[cit71] Long B. N., Beltrán-Leiva M. J., Celis-Barros C., Sperling J. M., Poe T. N., Baumbach R. E., Windorff C. J., Albrecht-Schönzart T. E. (2022). Cyclopentadienyl coordination induces unexpected ionic Am–N bonding in an americium bipyridyl complex. Nat. Commun..

[cit72] Evans W. J., Kozimor S. A., Ziller J. W., Kaltsoyannis N. (2004). Structure, Reactivity, and Density Functional Theory Analysis of the Six-Electron Reductant, [(C_5_Me_5_)_2_U]_2_(μ*-*η^6^:η^6^-C_6_H_6_), Synthesized *via* a New Mode of (C_5_Me_5_)_3_M Reactivity. J. Am. Chem. Soc..

[cit73] Barker B. J., Sears P. G. (1974). Conductance behavior of some ammonium and partially substituted ammonium tetraphenylborates in 3-methyl-2-oxazolidone and 3-*tert*-butyl-2-oxazolidone at 25.deg. J. Phys. Chem..

[cit74] Demir S., Zadrozny J. M., Nippe M., Long J. R. (2012). Exchange Coupling and Magnetic Blocking in Bipyrimidyl Radical-Bridged Dilanthanide Complexes. J. Am. Chem. Soc..

[cit75] Evans W. J., Davis B. L., Champagne T. M., Ziller J. W. (2006). C–H bond activation through steric crowding of normally inert ligands in the sterically crowded gadolinium and yttrium (C_5_Me_5_)_3_M complexes. Proc. Natl. Acad. Sci. U. S. A..

[cit76] Sheldrick G. M. (2015). SHELXT – integrated space-group and crystal-structure determination. Acta Crystallogr., Sect. A.

[cit77] Dolomanov O. V., Bourhis L. J., Gildea R. J., Howard J. A. K., Puschmann H. (2009). OLEX2: a complete structure solution, refinement and analysis program. J. Appl. Crystallogr..

[cit78] Stoll S., Schweiger A. (2006). EasySpin, a comprehensive software package for spectral simulation and analysis in EPR. J. Magn. Reson..

[cit79] Bain G. A., Berry J. F. (2008). Diamagnetic Corrections and Pascal's Constants. J. Chem. Educ..

[cit80] Neese F. (2012). The ORCA program system. Wiley Interdiscip. Rev.: Comput. Mol. Sci..

[cit81] Neese F. (2022). Software update: the ORCA program system—version 5.0. Wiley Interdiscip. Rev.: Comput. Mol. Sci..

[cit82] Stephens P. J., Devlin F. J., Chabalowski C. F., Frisch M. J. (1994). *Ab Initio* Calculation of Vibrational Absorption and Circular Dichroism Spectra Using Density Functional Force Fields. J. Phys. Chem..

[cit83] Becke A. D. (1992). The effect of the exchange-only gradient gradient correction. J. Chem. Phys..

[cit84] van Lenthe E., van Leeuwen R., Baerends E. J., Snijders J. G. (1996). Relativistic regular two-component Hamiltonians. Int. J. Quantum Chem..

[cit85] Weigend F., Ahlrichs R. (2005). Balanced basis sets of split valence, triple zeta valence and quadruple zeta valence quality for H to Rn: design and assessment of accuracy. Phys. Chem. Chem. Phys..

[cit86] Aravena D., Neese F., Pantazis D. A. (2016). Improved Segmented All-Electron Relativistically Contracted Basis Sets for the Lanthanides. J. Chem. Theory Comput..

[cit87] Rolfes J. D., Neese F., Pantazis D. A. (2020). All-electron scalar relativistic basis sets for the elements Rb–Xe. J. Comput. Chem..

[cit88] Weigend F. (2006). Accurate Coulomb-fitting basis sets for H to Rn. Phys. Chem. Chem. Phys..

[cit89] Pantazis D. A., Neese F. (2009). All-Electron Scalar Relativistic Basis Sets for the Lanthanides. J. Chem. Theory Comput..

[cit90] Grimme S., Ehrlich S., Goerigk L. (2011). Effect of the damping function in dispersion corrected density functional theory. J. Comput. Chem..

[cit91] Humphrey W., Dalke A., Schulten K. (1996). VMD: visual molecular dynamics. J. Mol. Graphics.

[cit92] Weigend F., Ahlrichs R. (2005). Balanced basis sets of split valence, triple zeta valence and quadruple zeta valence quality for H to Rn: design and assessment of accuracy. Phys. Chem. Chem. Phys..

[cit93] Rolfes J. D., Neese F., Pantazis D. A. (2020). All-electron scalar relativistic basis sets for the elements Rb–Xe. J. Comput. Chem..

[cit94] Aravena D., Neese F., Pantazis D. A. (2016). Improved Segmented All-Electron Relativistically Contracted Basis Sets for the Lanthanides. J. Chem. Theory Comput..

[cit95] Stoychev G. L., Auer A. A., Neese F. (2017). Automatic Generation of Auxiliary Basis Sets. J. Chem. Theory Comput..

[cit96] Angeli C., Cimiraglia R., Malrieu J.-P. (2002). *n*-electron valence state perturbation theory: a spinless formulation and an efficient implementation of the strongly contracted and of the partially contracted variants. J. Chem. Phys..

[cit97] Angeli C., Cimiraglia R., Malrieu J.-P. (2001). N-electron valence state perturbation theory: a fast implementation of the strongly contracted variant. Chem. Phys. Lett..

[cit98] Angeli C., Cimiraglia R., Evangelisti S., Leininger T., Malrieu J.-P. (2001). Introduction of *n*-electron valence states for multireference perturbation theory. J. Chem. Phys..

[cit99] Kollmar C., Sivalingam K., Guo Y., Neese F. (2021). An efficient implementation of the NEVPT2 and CASPT2 methods avoiding higher-order density matrices. J. Chem. Phys..

[cit100] Guo Y., Sivalingam K., Kollmar C., Neese F. (2021). Approximations of density matrices in N-electron valence state second-order perturbation theory (NEVPT2). II. The full rank NEVPT2 (FR-NEVPT2) formulation. J. Chem. Phys..

[cit101] Neese F., Petrenko T., Ganyushin D., Olbrich G. (2007). Advanced aspects of *ab initio* theoretical optical spectroscopy of transition metal complexes: multiplets, spin-orbit coupling and resonance Raman intensities. Coord. Chem. Rev..

[cit102] Atanasov M., Aravena D., Suturina E., Bill E., Maganas D., Neese F. (2015). First principles approach to the electronic structure, magnetic anisotropy and spin relaxation in mononuclear 3d-transition metal single molecule magnets. Coord. Chem. Rev..

[cit103] Neese F. (2005). Efficient and accurate approximations to the molecular spin-orbit coupling operator and their use in molecular *g*-tensor calculations. J. Chem. Phys..

[cit104] Visscher L., Dyall K. G. (1997). Dirac-Fock Atomic Electronic Structure Calculations Using Different Nuclear Charge Distributions. At. Data Nucl. Data Tables.

[cit105] Chibotaru L. F., Ungur L. (2012). *Ab initio* calculation of anisotropic magnetic properties of complexes. I. Unique definition of pseudospin Hamiltonians and their derivation. J. Chem. Phys..

[cit106] Shannon R. D. (1976). Revised effective ionic radii and systematic studies
of interatomic distances in halides and chalcogenides. Acta Crystallogr., Sect. A.

[cit107] Demir S., Boshart M. D., Corbey J. F., Woen D. H., Gonzalez M. I., Ziller J. W., Meihaus K. R., Long J. R., Evans W. J. (2017). Slow Magnetic Relaxation in a Dysprosium Ammonia Metallocene Complex. Inorg. Chem..

[cit108] Corner S. C., Gransbury G. K., Vitorica-Yrezabal I. J., Whitehead G. F. S., Chilton N. F., Mills D. P. (2024). Halobenzene Adducts of a Dysprosocenium Single-Molecule Magnet. Inorg. Chem..

[cit109] Roy L. E., Hughbanks T. (2006). Magnetic Coupling in Dinuclear Gd Complexes. J. Am. Chem. Soc..

[cit110] Evans P., Reta D., Goodwin C. A. P., Ortu F., Chilton N. F., Mills D. P. (2020). A double-dysprosocenium single-molecule magnet bound together with neutral ligands. Chem. Commun..

[cit111] Gransbury G. K., Corner S. C., Kragskow J. G. C., Evans P., Yeung H. M., Blackmore W. J. A., Whitehead G. F. S., Vitorica-Yrezabal I. J., Oakley M. S., Chilton N. F., Mills D. P. (2023). AtomAccess: A Predictive Tool for Molecular Design and Its Application to the Targeted Synthesis of Dysprosium Single-Molecule Magnets. J. Am. Chem. Soc..

[cit112] Corner S. C., Gransbury G. K., Mills D. P. (2025). Influence of weakly coordinating anions binding to the hexa-*tert*-butyl dysprosocenium cation. Dalton Trans..

[cit113] Jackson C. E., Moseley I. P., Martinez R., Sung S., Zadrozny J. M. (2021). A reaction-coordinate perspective of magnetic relaxation. Chem. Soc. Rev..

[cit114] Corner S. C., Gransbury G. K., Vitorica-Yrezabal I. J., Whitehead G. F. S., Chilton N. F., Mills D. P. (2024). Synthesis and Magnetic Properties of Bis-Halobenzene Decamethyldysprosocenium Cations. Inorg. Chem..

[cit115] Meng Y., Xiong J., Yang M., Qiao Y., Zhong Z., Sun H., Han J., Liu T., Wang B., Gao S. (2020). Experimental Determination of Magnetic Anisotropy in Exchange-Bias Dysprosium Metallocene Single-Molecule Magnets. Angew. Chem., Int. Ed..

[cit116] Deng W., Du S.-N., Chen Y.-C., Liu J.-L., Tong M.-L. (2025). Nontrivial Spin Dynamics Synergy in a Weakly Coupled Heteroanisotropic Dysprosium Chain-Based Array. J. Am. Chem. Soc..

[cit117] Soda T., Kitagawa Y., Onishi T., Takano Y., Shigeta Y., Nagao H., Yoshioka Y., Yamaguchi K. (2000). *Ab initio* computations of effective exchange integrals for H–H, H–He–H and Mn_2_O_2_ complex: comparison of broken-symmetry approaches. Chem. Phys. Lett..

[cit118] Yao X., An G., Li Y., Yan P., Li W., Li G. (2019). Effect of nuclearity and symmetry on the single-molecule magnets behavior of seven-coordinated β-diketonate Dy(III) complexes. J. Solid State Chem..

[cit119] Guo F.-S., Layfield R. A. (2017). Strong direct exchange coupling and single-molecule magnetism in indigo-bridged lanthanide dimers. Chem. Commun..

[cit120] Castellanos E., Benner F., Demir S. (2022). Taming salophen in rare earth metallocene chemistry. Inorg. Chem. Front..

[cit121] Bernot K., Bogani L., Caneschi A., Gatteschi D., Sessoli R. (2006). A Family of Rare-Earth-Based Single Chain Magnets: Playing with Anisotropy. J. Am. Chem. Soc..

[cit122] Bogani L., Sangregorio C., Sessoli R., Gatteschi D. (2005). Molecular engineering for single-chain-magnet behavior in a one-dimensional dysprosium-nitronyl nitroxide compound. Angew. Chem., Int. Ed..

[cit123] Barra A.-L., Brunel L.-C., Gatteschi D., Pardi L., Sessoli R. (1998). High-Frequency EPR Spectroscopy of Large Metal Ion Clusters: From Zero Field Splitting to Quantum Tunneling of the Magnetization. Acc. Chem. Res..

[cit124] Andersson K. K., Schmidt P. P., Katterle B., Strand K. R., Palmer A. E., Lee S.-K., Solomon E. I., Gräslund A., Barra A.-L. (2003). Examples of high-frequency EPR studies in bioinorganic chemistry. J. Biol. Inorg. Chem..

[cit125] Petrosyants S. P., Ilyukhin A. B., Babeshkin K. A., Ugolkova E. A., Minin V. V., Koroteev P. S., Efimov N. N. (2024). Influence of the Coordination Environment on the EPR Spectra of Mononuclear Gd Thiocyanates. Russ. J. Coord. Chem..

[cit126] Han T., Petersen J. B., Li Z.-H., Zhai Y.-Q., Kostopoulos A., Ortu F., McInnes E. J. L., Winpenny R. E. P., Zheng Y.-Z. (2020). Dimerized *p*-Semiquinone Radical Anions Stabilized by a Pair of Rare-Earth Metal Ions. Inorg. Chem..

[cit127] Delano F., Benner F., Jang S., Greer S. M., Demir S. (2024). Construction of intermolecular σ-hole interactions in rare earth metallocene complexes using a 2,3,4,5-tetraiodopyrrolyl anion. Chem. Sci..

[cit128] (a) Castellanos E. Benner F. , Deshapriya S. and Demir S., CCDC 2474596: Experimental Crystal Structure Determination, 2025, 10.5517/ccdc.csd.cc2p20ph

