# Supplementary material for: Magnetic hysteresis in 1D organometallic lanthanide chain compounds containing 4,4′-bipyridine
Source: Chem Sci. 2025 Sep 29;16(40):18616–31. doi: 10.1039/d5sc05460e (PMC12478534; doi:10.1039/d5sc05460e)
Supplement: SC-016-D5SC05460E-s001 [file SC-016-D5SC05460E-s001.pdf]

Supplementary Information (SI) for Chemical Science.
This journal is © The Royal Society of Chemistry 2025

Supplementary Information

for

Magnetic Hysteresis in 1D Organometallic Lanthanide Chain Compounds Containing 4,4′-Bipyridine

Ernesto Castellanos, Florian Benner, Saroshan Deshapriya, and Selvan Demir

Department of Chemistry, Michigan State University, 578 South Shaw Lane, East Lansing, Michigan 48824, USA

*Correspondence to: sdemir@chemistry.msu.edu (S.D.)

Table of Contents

Figure S1. Crystallisation glassware for $\{[Cp^*_2Ln(bpy)][BPh_4]\}_n$ complexes. S5

1. X-ray Crystallography **S6**

Table S1. Crystallographic data and structural refinement of $\{[Cp^*_2Ln(bpy)][BPh_4]\}_n$, where Ln = Gd (**1**), Tb (**2**), and Dy (**3**). S6
Figure S2. Polymeric structure of $\{[Cp^*_2Gd(bpy)][BPh_4]\}_n$, **1**. S7
Figure S3. Thermal ellipsoid plot of $\{[Cp^*_2Gd(bpy)][BPh_4]\}_n$, **1**. S8
Figure S4. Unit cell depiction of $\{[Cp^*_2Gd(bpy)][BPh_4]\}_n$, **1**, along the *ac* plane. S9
Figure S5. Unit cell depiction of $\{[Cp^*_2Gd(bpy)][BPh_4]\}_n$, **1**, along the *bc* plane. S10
Figure S6. Unit cell depiction of $\{[Cp^*_2Gd(bpy)][BPh_4]\}_n$, **1**, along the *ab* plane. S11
Figure S7. Crystal packing diagram of $\{[Cp^*_2Gd(bpy)][BPh_4]\}_n$, **1**, along the *ac* plane. S12
Figure S8. Crystal packing diagram of $\{[Cp^*_2Gd(bpy)][BPh_4]\}_n$, **1**, along the *bc* plane. S13
Figure S9. Crystal packing diagram of $\{[Cp^*_2Gd(bpy)][BPh_4]\}_n$, **1**, along the *ab* plane. S14
Figure S10. Polymeric structure of $\{[Cp^*_2Tb(bpy)][BPh_4]\}_n$, **2**. S15
Figure S11. Thermal ellipsoid plot of $\{[Cp^*_2Tb(bpy)][BPh_4]\}_n$, **2**. S16
Figure S12. Unit cell depiction of $\{[Cp^*_2Tb(bpy)][BPh_4]\}_n$, **2**, along the *ac* plane. S17
Figure S13. Unit cell depiction of $\{[Cp^*_2Tb(bpy)][BPh_4]\}_n$, **2**, along the *bc* plane. S18
Figure S14. Unit cell depiction of $\{[Cp^*_2Tb(bpy)][BPh_4]\}_n$, **2**, along the *ab* plane. S19
Figure S15. Crystal packing diagram of $\{[Cp^*_2Tb(bpy)][BPh_4]\}_n$, **2**, along the *ac* plane. S20
Figure S17. Crystal packing diagram of $\{[Cp^*_2Tb(bpy)][BPh_4]\}_n$, **2**, along the *bc* plane. S21
Figure S17. Crystal packing diagram of $\{[Cp^*_2Tb(bpy)][BPh_4]\}_n$, **2**, along the *ab* plane. S22
Figure S18. Polymeric structure of $\{[Cp^*_2Dy(bpy)][BPh_4]\}_n$, **3**. S23
Figure S19. Thermal ellipsoid plot of $\{[Cp^*_2Dy(bpy)][BPh_4]\}_n$, **3**. S24
Figure S20. Unit cell depiction of $\{[Cp^*_2Dy(bpy)][BPh_4]\}_n$, **3**, along the *ac* plane. S25
Figure S21. Unit cell depiction of $\{[Cp^*_2Dy(bpy)][BPh_4]\}_n$, **3**, along the *bc* plane. S26
Figure S22. Unit cell depiction of $\{[Cp^*_2Dy(bpy)][BPh_4]\}_n$, **3**, along the *ab* plane. S27
Figure S23. Crystal packing diagram of $\{[Cp^*_2Dy(bpy)][BPh_4]\}_n$, **3**, along the *ac* plane. S28
Figure S24. Crystal packing diagram of $\{[Cp^*_2Dy(bpy)][BPh_4]\}_n$, **3**, S29

along the *bc* plane.
Figure S25. Crystal packing diagram of {[Cp*$_2$Dy(bpy)][BPh$_4$]}$_n$, **3**, along the *ab* plane. S30
Table S2. Metrical parameters for the crystallographic distances and angles in {[Cp*$_2$Ln(bpy)][BPh$_4$]}$_n$, where Ln = Gd (**1**), Tb (**2**), and Dy (**3**). S31

2. IR Spectroscopy **S32**
Figure S26. FTIR spectrum of {[Cp*$_2$Gd(bpy)][BPh$_4$]}$_n$, **1**. S32
Figure S27. FTIR spectrum of {[Cp*$_2$Tb(bpy)][BPh$_4$]}$_n$, **2**. S33
Figure S28. FTIR spectrum of {[Cp*$_2$Dy(bpy)][BPh$_4$]}$_n$, **3**. S34
Figure S29. Superimposed FTIR spectra of {[Cp*$_2$Ln(bpy)][BPh$_4$]}$_n$, where Ln = Gd (**1**), Tb (**2**), and Dy (**3**). S35
Figure S30. Magnification of the FTIR spectra of {[Cp*$_2$Ln(bpy)][BPh$_4$]}$_n$, where Ln = Gd (**1**), Tb (**2**), and Dy (**3**). S36

3. Magnetic Data **S37**
Table S3. Select examples of mononuclear dysprosium metallocene complexes bearing neutral ligands or weakly coordinating anions. S37
Table S4. Select examples of multinuclear dysprosium metallocene complexes bearing diamagnetic bridging ligands. S38
Figure S31. Variable-temperature dc magnetic susceptibility data of {[Cp*$_2$Gd(bpy)][BPh$_4$]}$_n$, **1**. S39
Figure S32. Variable-temperature dc magnetic susceptibility data of {[Cp*$_2$Tb(bpy)][BPh$_4$]}$_n$, **2**. S40
Figure S33. Variable-temperature dc magnetic susceptibility data of {[Cp*$_2$Dy(bpy)][BPh$_4$]}$_n$, **3**. S41
Figure S34. Variable-temperature dc magnetic susceptibility data of {[Cp*$_2$Ln(bpy)][BPh$_4$]}$_n$, where Ln = Gd (**1**), Tb (**2**), and Dy (**3**) under a 0.5 T applied dc field. S42
Figure S35. Variable-temperature dc magnetic susceptibility data of {[Cp*$_2$Ln(bpy)][BPh$_4$]}$_n$, where Ln = Gd (**1**), Tb (**2**), and Dy (**3**) under a 1.0 T applied dc field. S43
Figure S36. Cole–Cole plots for ac susceptibility collected under zero applied dc field for {[Cp*$_2$Dy(bpy)][BPh$_4$]}$_n$, **3**. S44
Figure S37. Cole–Cole plots for ac susceptibility collected under zero applied dc field for {[Cp*$_2$Dy(bpy)][BPh$_4$]}$_n$, **3**, shown for the temperature range 9 to 39 K. S45
Figure S38. Plots of the natural log of the relaxation time, τ, vs. inverse temperature, 1/T, for {[Cp*$_2$Dy(bpy)][BPh$_4$]}$_n$, **3**. S46
Figure S39. Field-dependent magnetisation data for {[Cp*$_2$Gd(bpy)][BPh$_4$]}$_n$, **1**, collected from 0 to 7 T between 2 K and 10 K. S47
Figure S40. Reduced magnetisation data for {[Cp*$_2$Gd(bpy)][BPh$_4$]}$_n$, **1**, collected from 2 to 10 K, between 0 and 7 T. S48
Figure S41. Field-dependent magnetisation data for {[Cp*$_2$Tb(bpy)][BPh$_4$]}$_n$, **2**, collected from 0 to 7 T between 2 K and 10 K. S49
Figure S42. Reduced magnetisation data for S50

{[$Cp^*_2Tb(bpy)$][BPh_4]}$_n$, **2**, collected from 2 to 10 K, between 0 and 7 T.
Figure S43. Field-dependent magnetisation data for {[$Cp^*_2Dy(bpy)$][BPh_4]}$_n$, **3**, collected from 0 to 7 T between 2 K and 10 K. S51
Figure S44. Reduced magnetisation data for {[$Cp^*_2Dy(bpy)$][BPh_4]}$_n$, **3**, collected from 2 to 10 K, between 0 and 7 T. S52
Figure S45. Plot of magnetisation (*M*) vs dc magnetic field (*H*) for {[$Cp^*_2Tb(bpy)$][BPh_4]}$_n$, **2**, at 1.8 K. S53
Figure S46. Plot of magnetisation (*M*) vs dc magnetic field (*H*) for {[$Cp^*_2Dy(bpy)$][BPh_4]}$_n$, **3**, between 2 and 8 K. S54
Figure S47. Plot of magnetisation (*M*) vs dc magnetic field (*H*) for {[$Cp^*_2Dy(bpy)$][BPh_4]}$_n$, **3**, at 2 K. S55

4. DFT Calculations **S56**
Figure S48. Calculated intrachain exchange coupling pathways in {[$Cp^*_2Gd(bpy)$][BPh_4]}$_n$, **1**. S56
Figure S49. Calculated interchain exchange coupling pathways in {[$Cp^*_2Gd(bpy)$][BPh_4]}$_n$, **1**. S57

5. EPR Spectroscopy **S58**
Figure S50. First integral of X-band EPR spectra of {[$Cp^*_2Gd(bpy)$][BPh_4]}$_n$, **1**. S58

6. *Ab initio* Calculations **S59**
Table S5. Computed magnetic moment, *g*-tensors, and wavefunction composition for the calculated Kramers doublets of the theoretical [$Cp^*_2Dy(bpy)_2$]$^+$ fragment. S59
Table S6. Calculated Crystal field parameters of the theoretical [$Cp^*_2Dy(bpy)_2$]$^+$ fragment. S60
Table S7. Calculated average transition dipole moments of the theoretical [$Cp^*_2Dy(bpy)_2$]$^+$ fragment. S61
Figure S51. Calculated relaxation barrier for the theoretical [$Cp^*_2Dy(bpy)_2$]$^+$ fragment. S62
Figure S52. Variable-temperature dc magnetic susceptibility data of {[$Cp^*_2Dy(bpy)$][BPh_4]}$_n$, **3**, collected under a 0.1 T applied dc field with *ab initio*-calculated susceptibility. S63
Table S8. Coordinates of the optimised [$Cp^*_2Dy(bpy)_2$]$^+$ fragment. S64

7. References **S66**

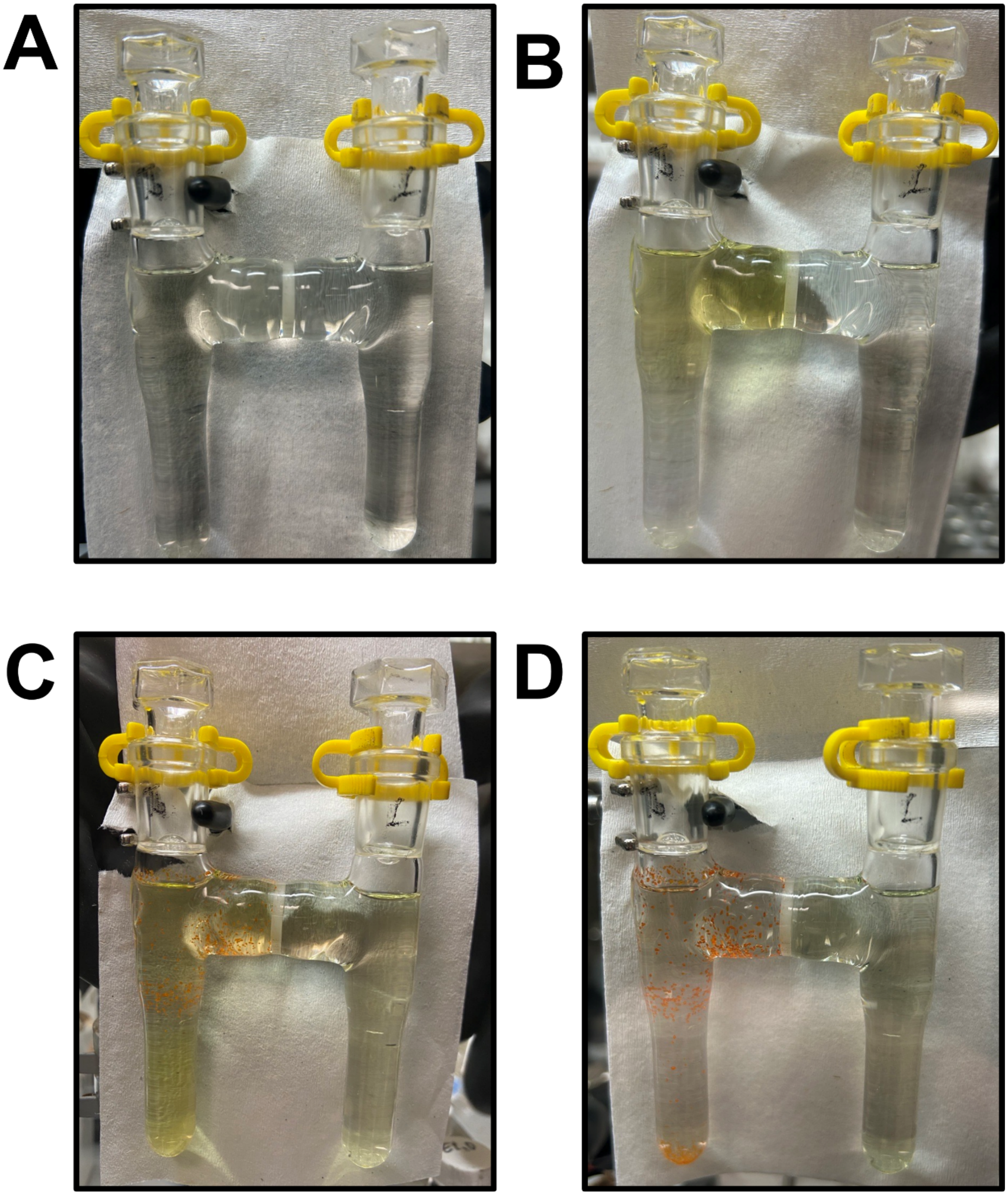

Figure S1. Crystallisation of $\{[Cp^*_2Tb(bpy)][BPh_4]\}_n$•$3(C_4H_8O)$, **2**•$3(C_4H_8O)$, from the diffusion of THF solutions of $Cp^*_2Tb(BPh_4)$ and 4,4′-bipyridine using an H-shaped tube with a fine porosity glass frit bridging the two sides. Orange, block-shaped crystals are observed within five days, however increased crystalline yields were obtained after allowing for the slow diffusion to proceed for 14 days at room temperature. Photos were taken on the first (A), second (B), fifth (C), and fourteenth (D) day after initiating the reaction and sealing the H-tube.

1 X-ray Crystallography

Table S1. Crystallographic data and structural refinement of {[Cp*$_2$Ln(bpy)][BPh$_4$]}$_n$, where Ln = Gd (**1**), Tb (**2**), and Dy (**3**). **1**–**3** crystallised with three tetrahydrofuran (THF) molecule in the lattice as: {[Cp*$_2$Ln(bpy)][BPh$_4$]}$_n$•3(C_4H_8O).

	**1**	**2**	**3**
CCDC Accession Codes	2474597	2474596	2474598
Empirical formula	$C_{66}H_{82}BN_2O_3Gd$	$C_{66}H_{82}BN_2O_3Tb$	$C_{66}H_{82}BN_2O_3Dy$
Formula weight	1119.39	1121.06	1124.64
Temperature/K	100.00(10)	99.8(9)	100.00(10)
Crystal system	monoclinic	monoclinic	monoclinic
Space group	*C*2/*c*	*C*2/*c*	*C*2/*c*
a/Å	17.4145(8)	17.6459(6)	17.6459(6)
b/Å	21.4824(5)	21.3539(6)	21.3539(6)
c/Å	17.5221(7)	17.3232(7)	17.3232(7)
α/°	90	90	90
β/°	119.353(6)	120.680(5)	120.680(5)
γ/°	90	90	90
Volume/Å^3	5713.6(5)	5613.9(4)	5613.9(4)
Z	4	4	4
ρ_{calc}g/cm^3	1.301	1.326	1.331
μ/mm^{-1}	1.208	1.308	7.481
F(000)	2340.0	2344.0	2348.0
Crystal size/mm^3	0.262 × 0.168 × 0.148	0.206 × 0.154 × 0.103	0.228 × 0.153 × 0.13
Radiation	MoKα (λ = 0.71073)	Mo Kα (λ = 0.71073)	Cu Kα (λ = 1.54184)
2Θ range for data collection/°	4.992 to 57.65	5.99 to 50.774	7.142 to 154.65
Index ranges	-22 ≤ h ≤ 23, -28 ≤ k ≤ 27, -23 ≤ l ≤ 23	-21 ≤ h ≤ 21, -25 ≤ k ≤ 25, -20 ≤ l ≤ 20	-19 ≤ h ≤ 22, -21 ≤ k ≤ 26, -21 ≤ l ≤ 21
Reflections collected	33311	47856	22393
Independent reflections	6731 [R_{int} = 0.0387, R_{sigma} = 0.0284]	5101 [R_{int} = 0.0544, R_{sigma} = 0.0237]	5701 [R_{int} = 0.0430, R_{sigma} = 0.0277]
Data/restraints/parameters	6731/721/432	5101/721/438	5701/721/432
Goodness-of-fit on F^2	1.135	1.186	1.064
Final R indexes [I>=2σ (I)]	R_1 = 0.0586, wR_2 = 0.1375	R_1 = 0.0592, wR_2 = 0.1390	R_1 = 0.0534, wR_2 = 0.1429
Final R indexes [all data]	R_1 = 0.0789, wR_2 = 0.1581	R_1 = 0.0637, wR_2 = 0.1412	R_1 = 0.0609, wR_2 = 0.1483
Largest diff. peak/hole / e Å^{-3}	3.06/-2.07	2.47/-1.42	1.07/-0.97

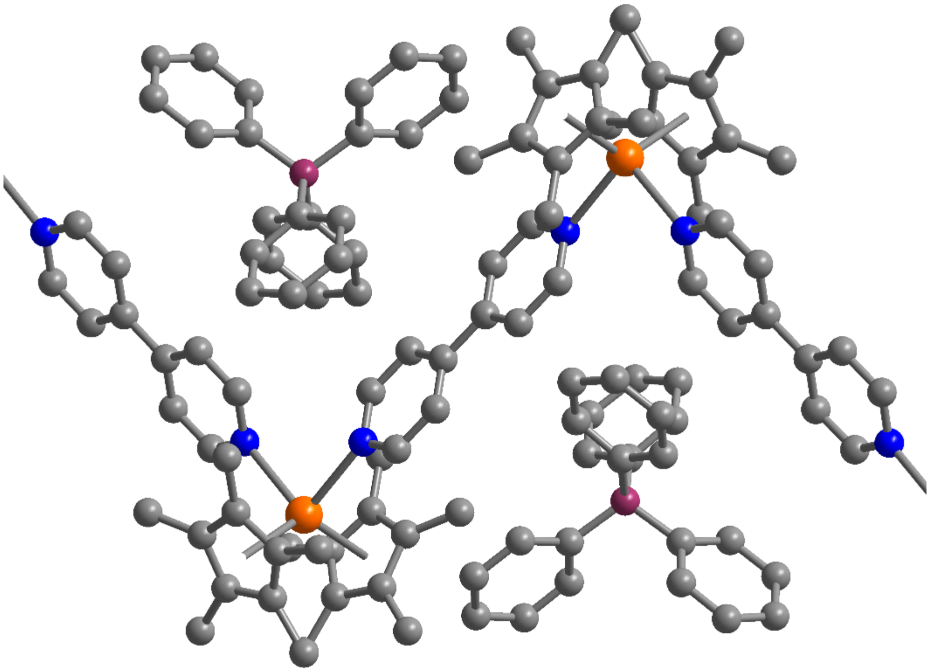

Figure S2. Polymeric structure of $\{[Cp^*_2Gd(bpy)][BPh_4]\}_n$, **1**, in a crystal of $\{[Cp^*_2Gd(bpy)][BPh_4]\}_n \cdot 3(C_4H_8O)$. Orange, blue, grey, and purple spheres represent Gd, N, C, and B atoms, respectively. Hydrogen atoms and solvent molecules in the crystal lattice have been omitted for clarity.

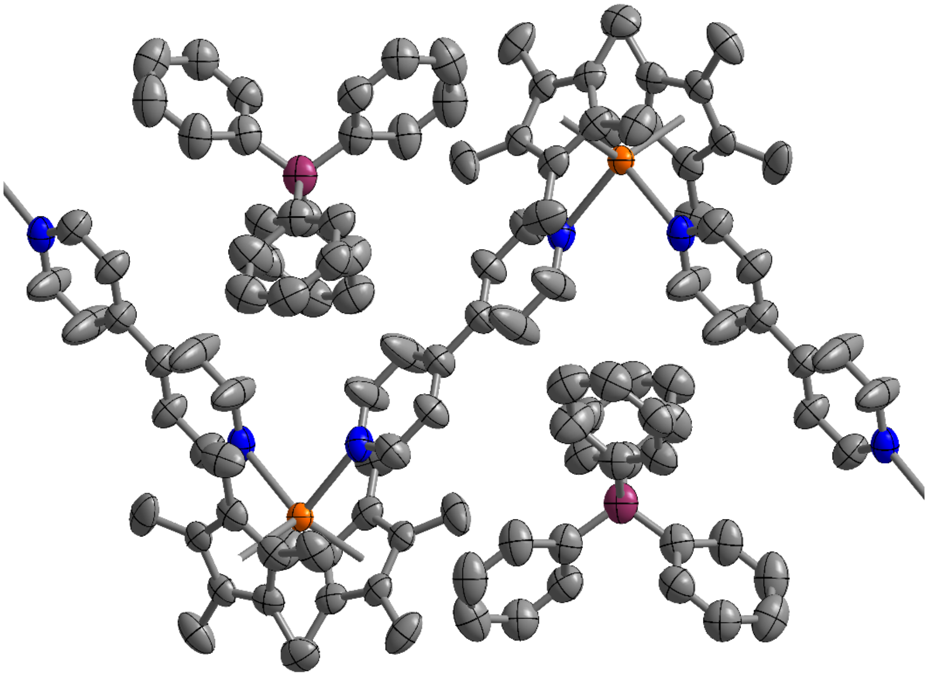

Figure S3. Structure of {[Cp*$_2$Gd(bpy)][BPh$_4$]}$_n$, **1**, in a crystal of {[Cp*$_2$Gd(bpy)][BPh$_4$]}$_n$•3(C_4H_8O) with thermal ellipsoids drawn at 50% probability level. Orange, blue, grey, and purple spheres represent Gd, N, C, and B atoms, respectively. Hydrogen atoms and solvent molecules in the crystal lattice have been omitted for clarity.

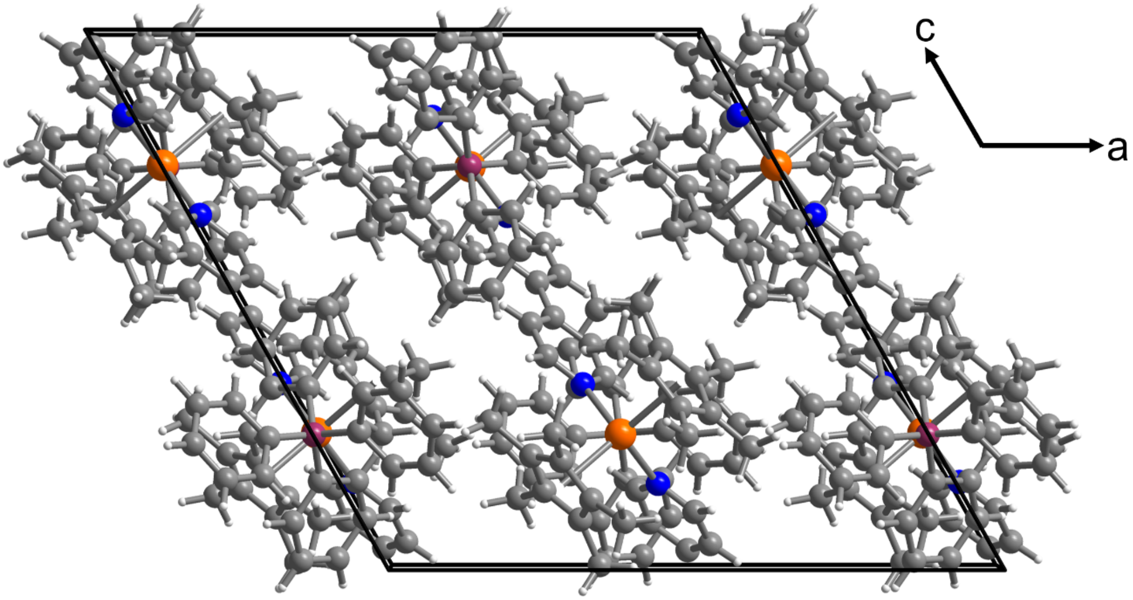

Figure S4. Unit cell depiction of {[Cp*$_2$Gd(bpy)][BPh$_4$]}$_n$, **1**, in a crystal of {[Cp*$_2$Gd(bpy)][BPh$_4$]}$_n$•3(C_4H_8O) along the *ac* plane. Orange, blue, grey, purple, and white-grey spheres represent Gd, N, C, B, and H atoms, respectively. Solvent molecules in the crystal lattice have been omitted for clarity.

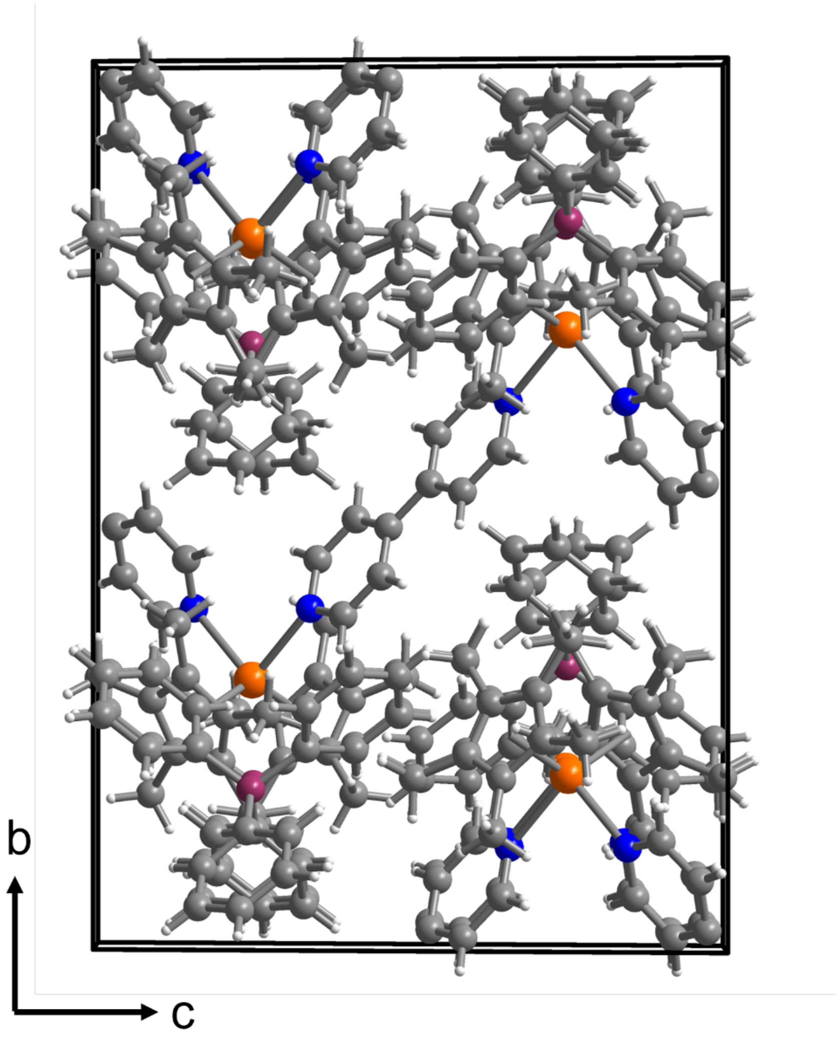

Figure S5. Unit cell depiction of $\{[Cp^*_2Gd(bpy)][BPh_4]\}_n$, **1**, in a crystal of $\{[Cp^*_2Gd(bpy)][BPh_4]\}_n \cdot 3(C_4H_8O)$ along the *bc* plane. Orange, blue, grey, purple, and white-grey spheres represent Gd, N, C, B, and H atoms, respectively. Solvent molecules in the crystal lattice have been omitted for clarity.

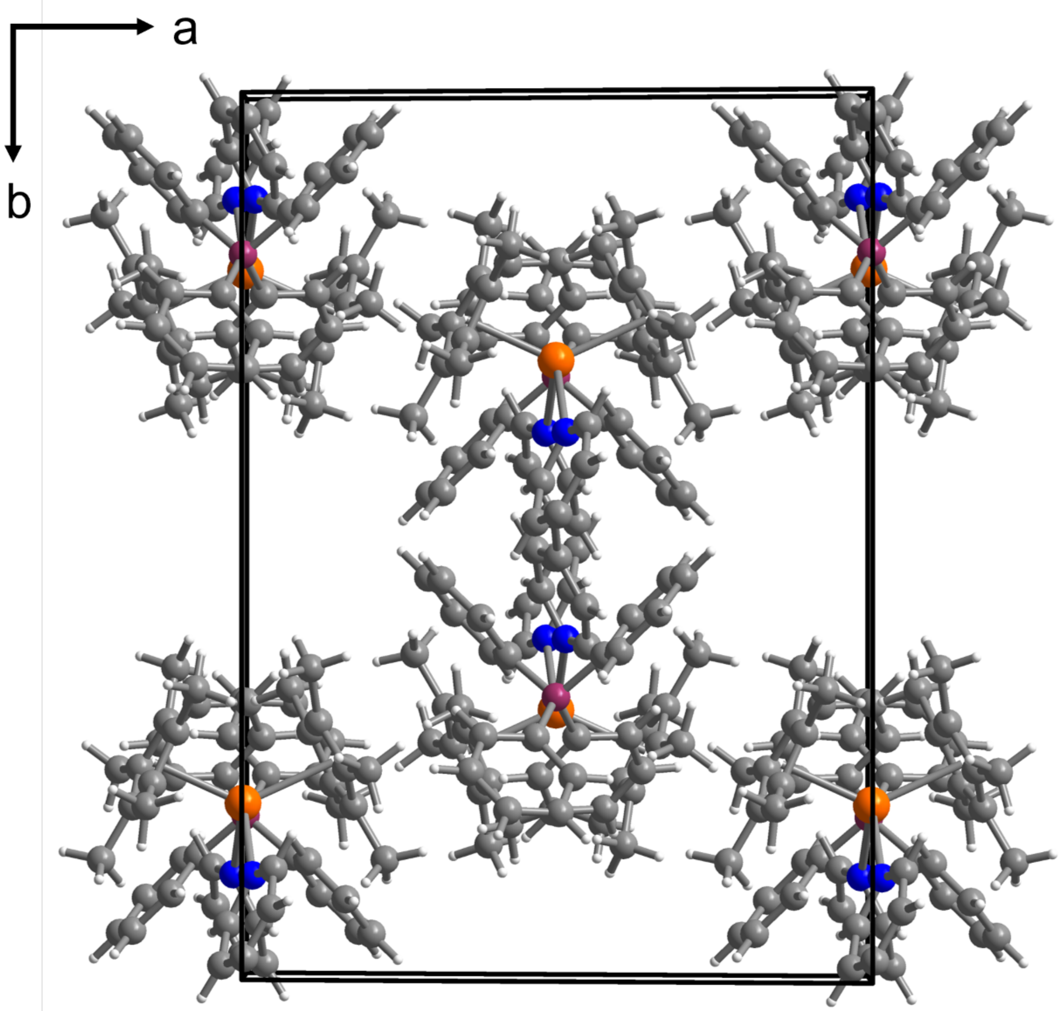

Figure S6. Unit cell depiction of {[Cp*$_2$Gd(bpy)][BPh$_4$]}$_n$, **1**, in a crystal of {[Cp*$_2$Gd(bpy)][BPh$_4$]}$_n$•3(C_4H_8O) along the *ab* plane. Orange, blue, grey, purple, and white-grey spheres represent Gd, N, C, B, and H atoms, respectively. Solvent molecules in the crystal lattice have been omitted for clarity.

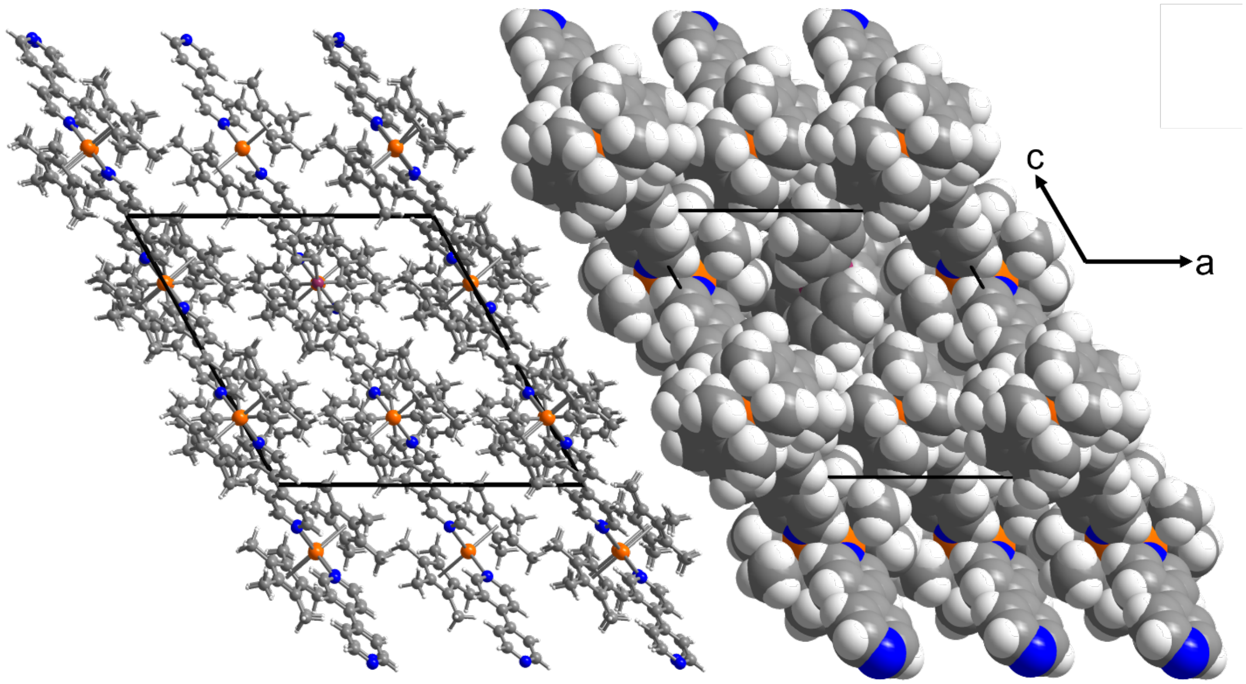

Figure S7. Crystal packing diagram of {[Cp*$_2$Gd(bpy)][BPh$_4$]}$_n$, **1**, in a crystal of {[Cp*$_2$Gd(bpy)][BPh$_4$]}$_n$•3(C_4H_8O) along the *ac* plane with atoms displayed as standard spheres (left), and as a space-filling model (right). Orange, blue, grey, purple, and white-grey spheres represent Gd, N, C, B, and H atoms, respectively. Solvent molecules in the crystal lattice have been omitted for clarity.

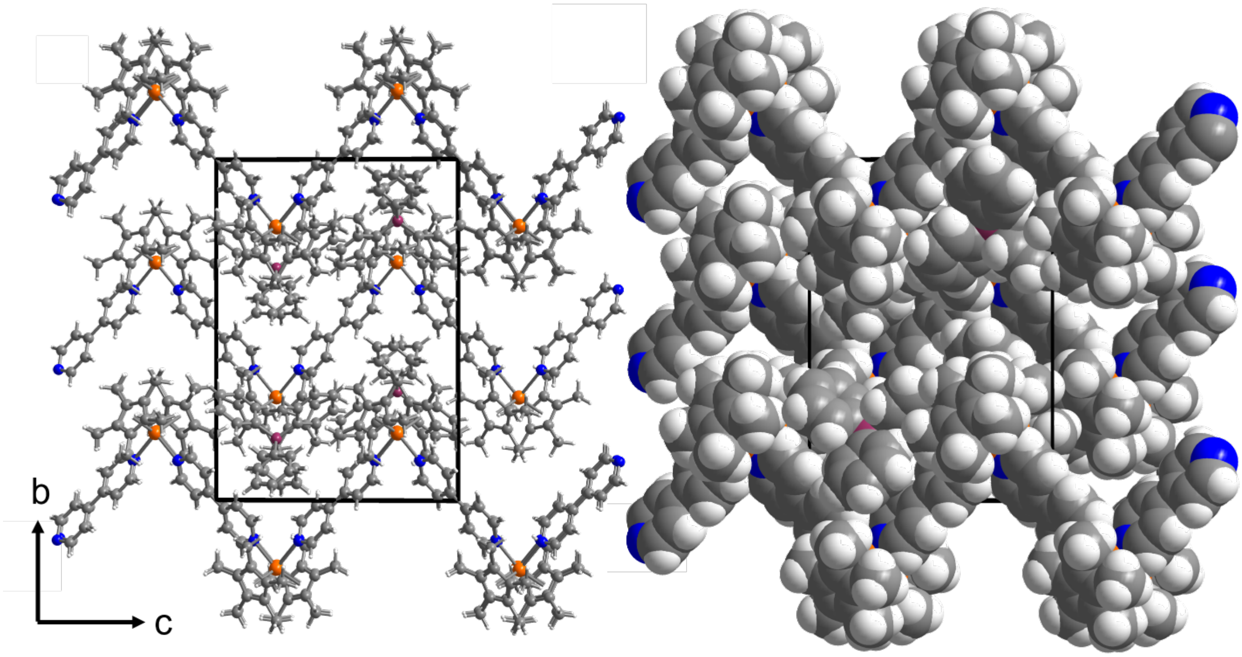

Figure S8. Crystal packing diagram of {[Cp*$_2$Gd(bpy)][BPh$_4$]}$_n$, **1**, in a crystal of {[Cp*$_2$Gd(bpy)][BPh$_4$]}$_n$•3(C_4H_8O) along the *bc* plane with atoms displayed as standard spheres (left), and as a space-filling model (right). Orange, blue, grey, purple, and white-grey spheres represent Gd, N, C, B, and H atoms, respectively. Solvent molecules in the crystal lattice have been omitted for clarity.

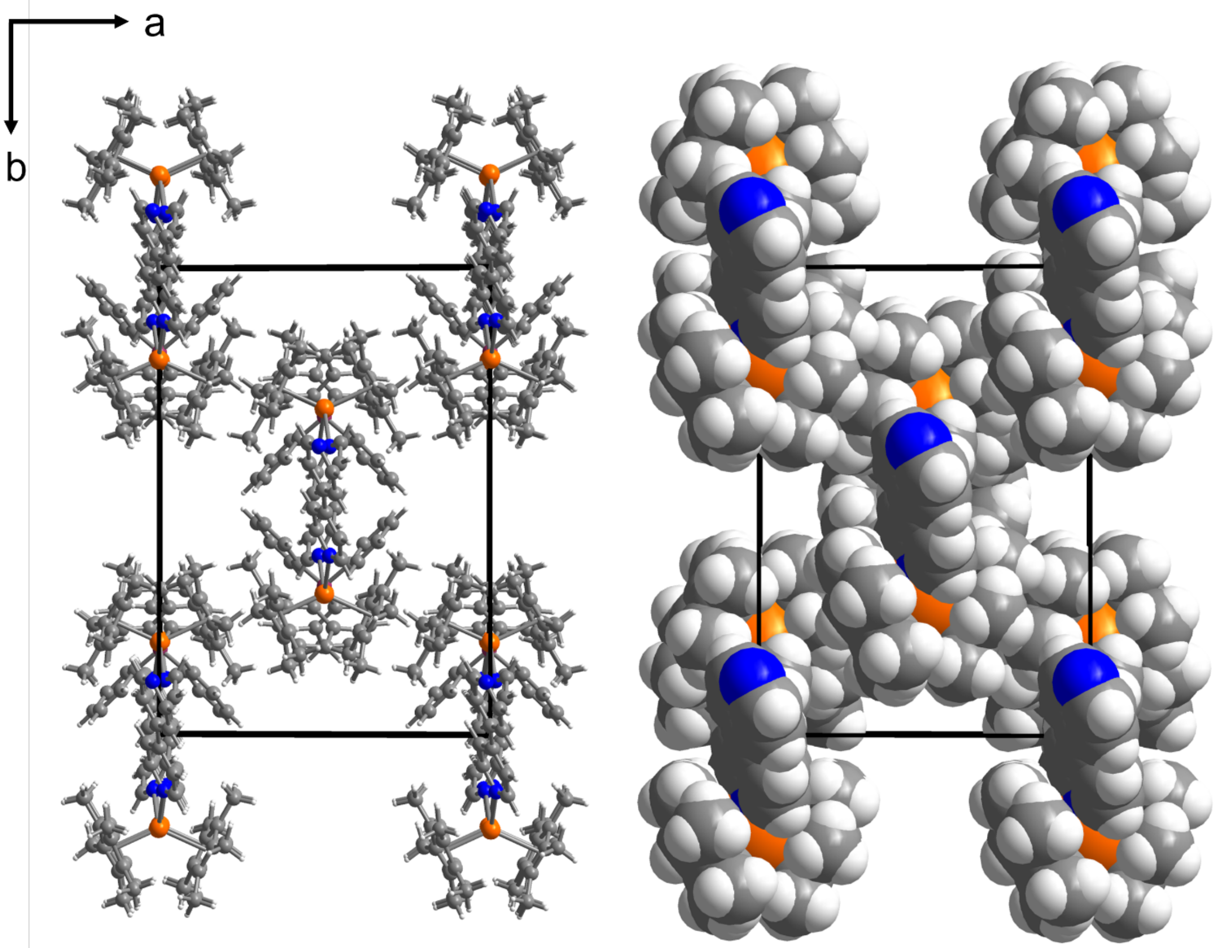

Figure S9. Crystal packing diagram of {[Cp*$_2$Gd(bpy)][BPh$_4$]}$_n$, **1**, in a crystal of {[Cp*$_2$Gd(bpy)][BPh$_4$]}$_n$•3(C_4H_8O) along the *ab* plane with atoms displayed as standard spheres (left), and as a space-filling model (right). Orange, blue, grey, purple, and white-grey spheres represent Gd, N, C, B, and H atoms, respectively. Solvent molecules in the crystal lattice have been omitted for clarity.

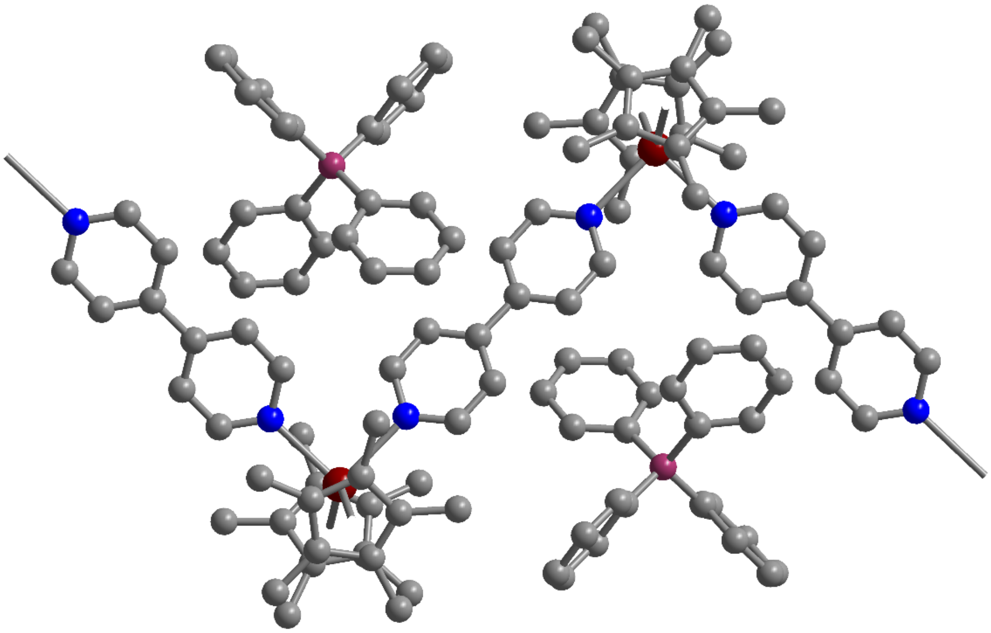

Figure S10. Polymeric structure of $\{[Cp^*_2Tb(bpy)][BPh_4]\}_n$, **2**, in a crystal of $\{[Cp^*_2Tb(bpy)][BPh_4]\}_n \cdot 3(C_4H_8O)$. Maroon, blue, grey, and purple spheres represent Tb, N, C, and B atoms, respectively. Hydrogen atoms and solvent molecules in the crystal lattice have been omitted for clarity.

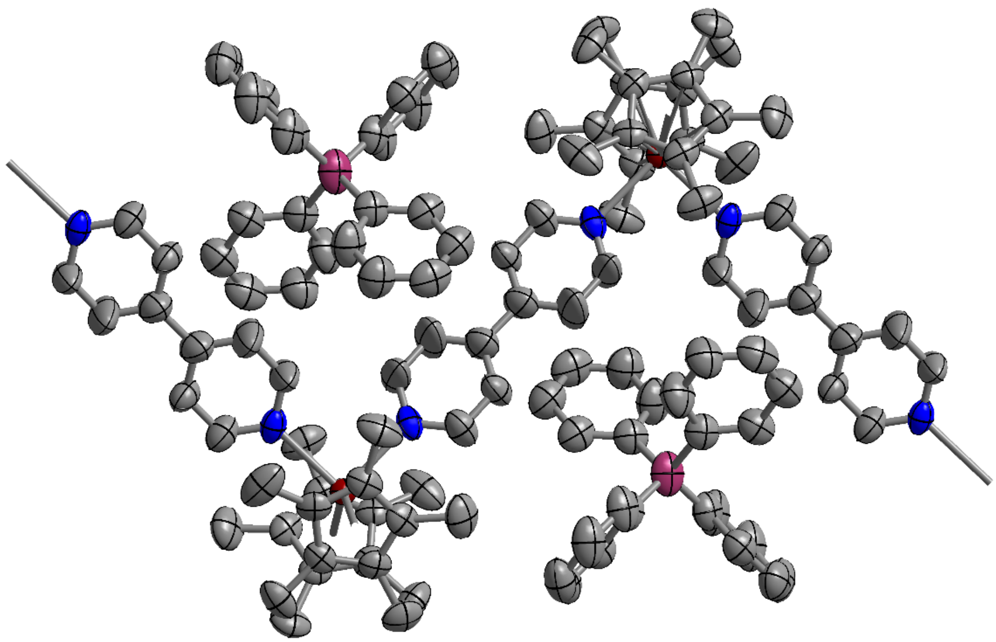

Figure S11. Structure of $\{[Cp^*_2Tb(bpy)][BPh_4]\}_n$, **2**, in a crystal of $\{[Cp^*_2Tb(bpy)][BPh_4]\}_n \cdot 3(C_4H_8O)$ with thermal ellipsoids drawn at 50% probability level. Maroon, blue, grey, and purple spheres represent Tb, N, C, and B atoms, respectively. Hydrogen atoms and solvent molecules in the crystal lattice have been omitted for clarity.

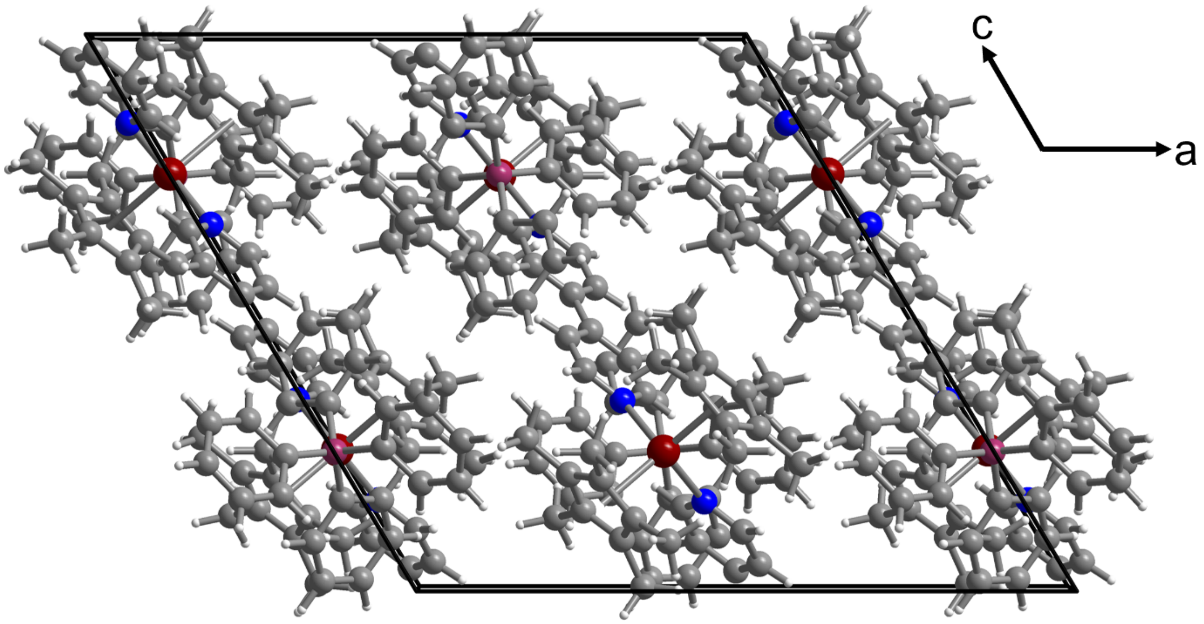

Figure S12. Unit cell depiction of {[Cp*$_2$Tb(bpy)][BPh$_4$]}$_n$, **2**, in a crystal of {[Cp*$_2$Tb(bpy)][BPh$_4$]}$_n$•3(C_4H_8O) along the *ac* plane. Maroon, blue, grey, purple, and white-grey spheres represent Tb, N, C, B, and H atoms, respectively. Solvent molecules in the crystal lattice have been omitted for clarity.

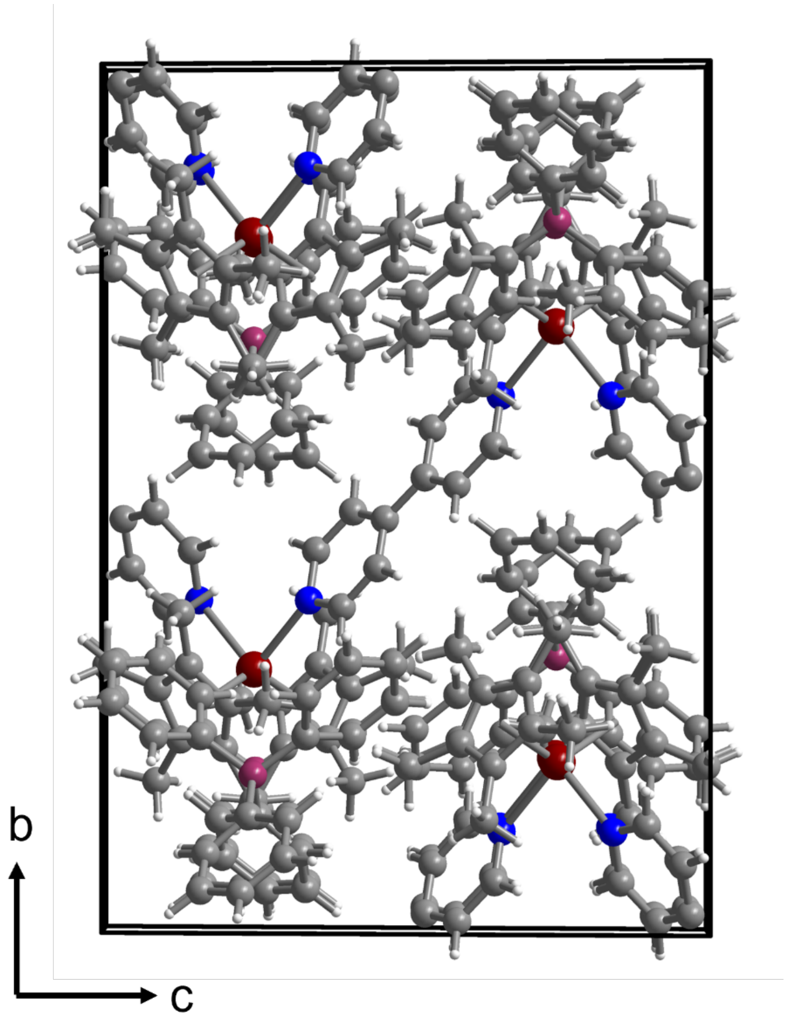

Figure S13. Unit cell depiction of {[Cp*$_2$Tb(bpy)][BPh_4]}$_n$, **2**, in a crystal of {[Cp*$_2$Tb(bpy)][BPh_4]}$_n$•3(C_4H_8O) along the *bc* plane. Maroon, blue, grey, purple, and white-grey spheres represent Tb, N, C, B, and H atoms, respectively. Solvent molecules in the crystal lattice have been omitted for clarity.

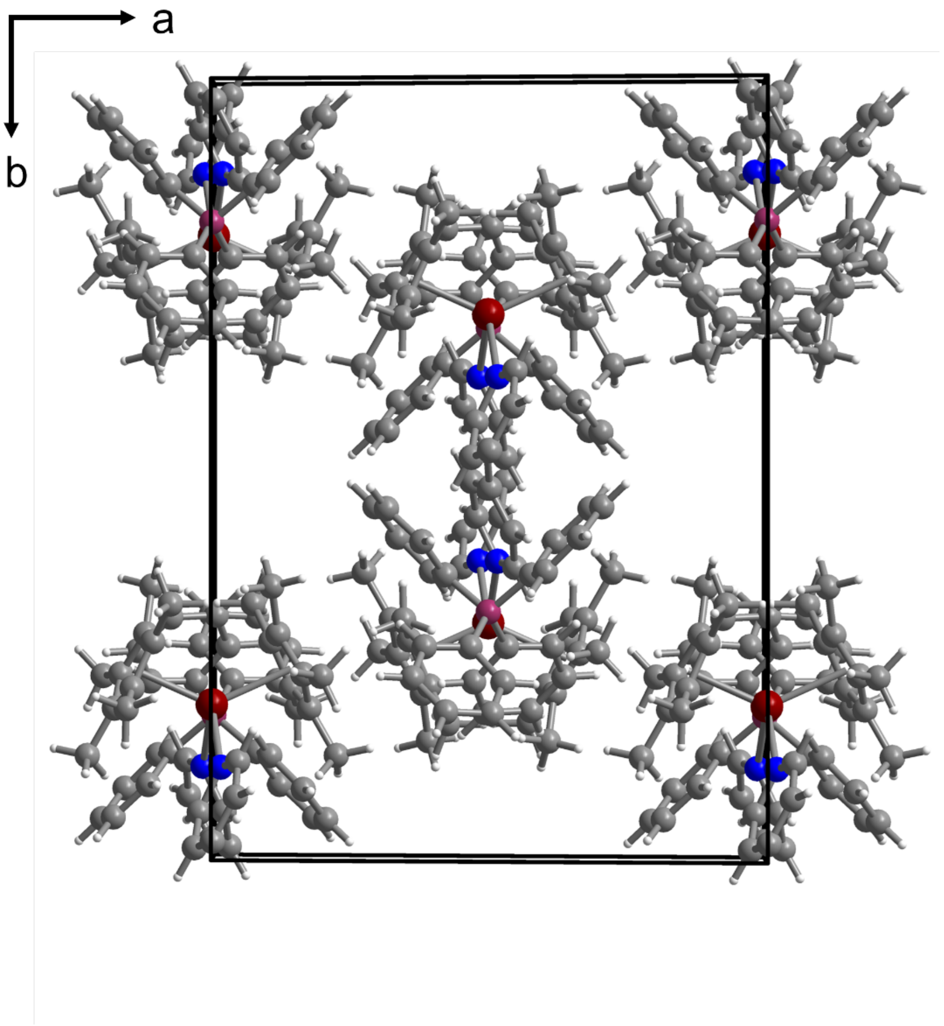

Figure S14. Unit cell depiction of {[Cp*$_2$Tb(bpy)][BPh$_4$]}$_n$, **2**, in a crystal of {[Cp*$_2$Tb(bpy)][BPh$_4$]}$_n$•3(C_4H_8O) along the *ab* plane. Maroon, blue, grey, purple, and white-grey spheres represent Tb, N, C, B, and H atoms, respectively. Solvent molecules in the crystal lattice have been omitted for clarity.

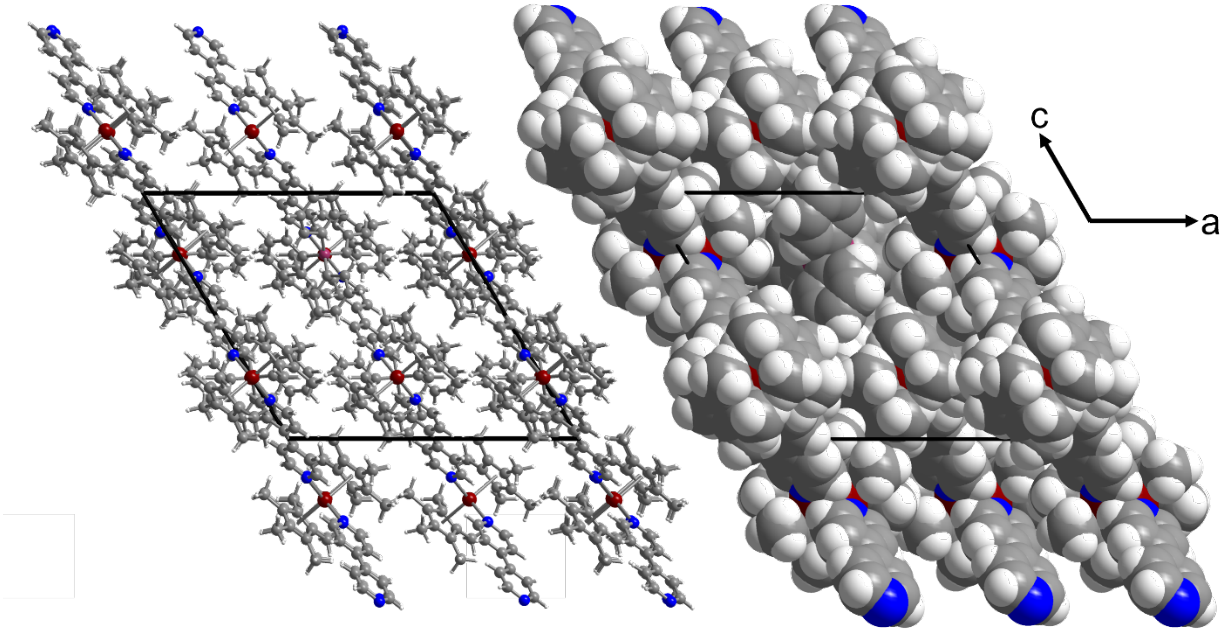

Figure S15. Crystal packing diagram of {[Cp*$_2$Tb(bpy)][BPh$_4$]}$_n$, **2**, in a crystal of {[Cp*$_2$Tb(bpy)][BPh$_4$]}$_n$•3(C_4H_8O) along the *ac* plane with atoms displayed as standard spheres (left), and as a space-filling model (right). Maroon, blue, grey, purple, and white-grey spheres represent Tb, N, C, B, and H atoms, respectively. Solvent molecules in the crystal lattice have been omitted for clarity.

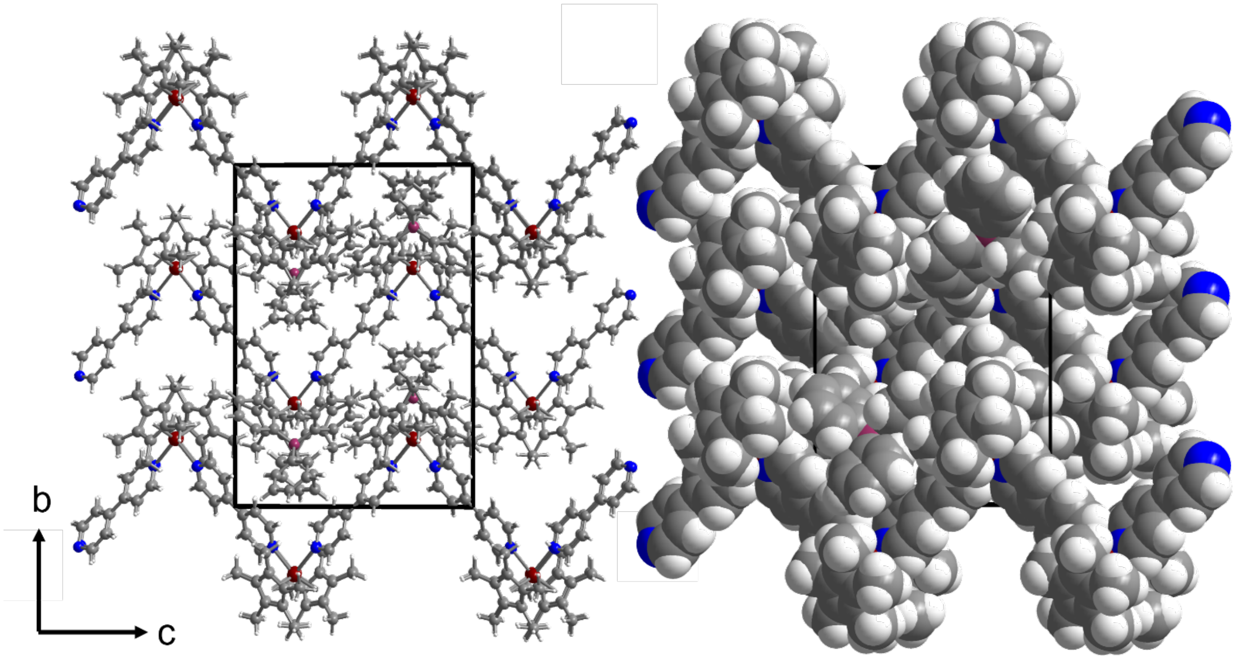

Figure S16. Unit cell depiction of {[Cp*$_2$Tb(bpy)][BPh$_4$]}$_n$, **2**, in a crystal of {[Cp*$_2$Tb(bpy)][BPh$_4$]}$_n$•3(C_4H_8O) along the *bc* plane with atoms displayed as standard spheres (left), and as a space-filling model (right). Maroon, blue, grey, purple, and white-grey spheres represent Tb, N, C, B, and H atoms, respectively. Solvent molecules in the crystal lattice have been omitted for clarity.

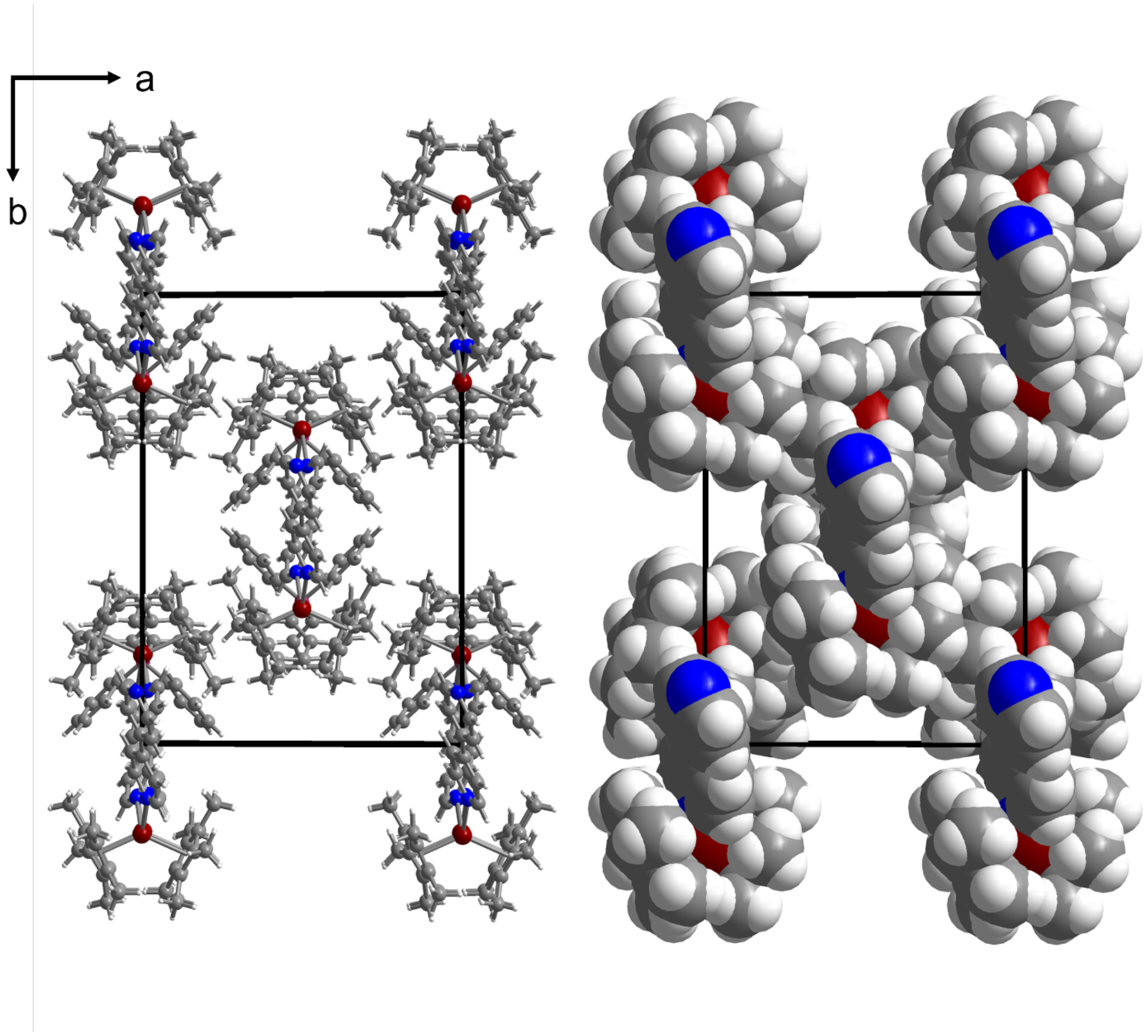

Figure S17. Crystal packing diagram of {[Cp*$_2$Tb(bpy)][BPh$_4$]}$_n$, **2**, in a crystal of {[Cp*$_2$Tb(bpy)][BPh$_4$]}$_n$•3(C_4H_8O) along the *ab* plane with atoms displayed as standard spheres (left), and as a space-filling model (right). Maroon, blue, grey, purple, and white-grey spheres represent Tb, N, C, B, and H atoms, respectively. Solvent molecules in the crystal lattice have been omitted for clarity.

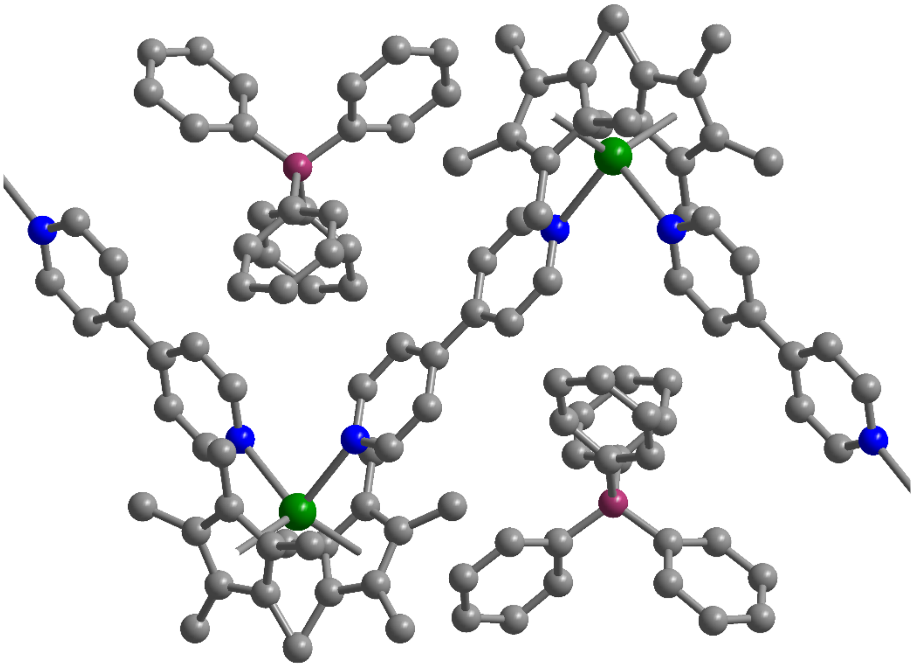

Figure S18. Polymeric structure of {[Cp*$_2$Dy(bpy)][BPh$_4$]}$_n$, **3**, in a crystal of {[Cp*$_2$Dy(bpy)][BPh$_4$]}$_n$•3(C_4H_8O). Green, blue, grey, and purple spheres represent Dy, N, C, and B atoms, respectively. Hydrogen atoms and solvent molecules in the crystal lattice have been omitted for clarity.

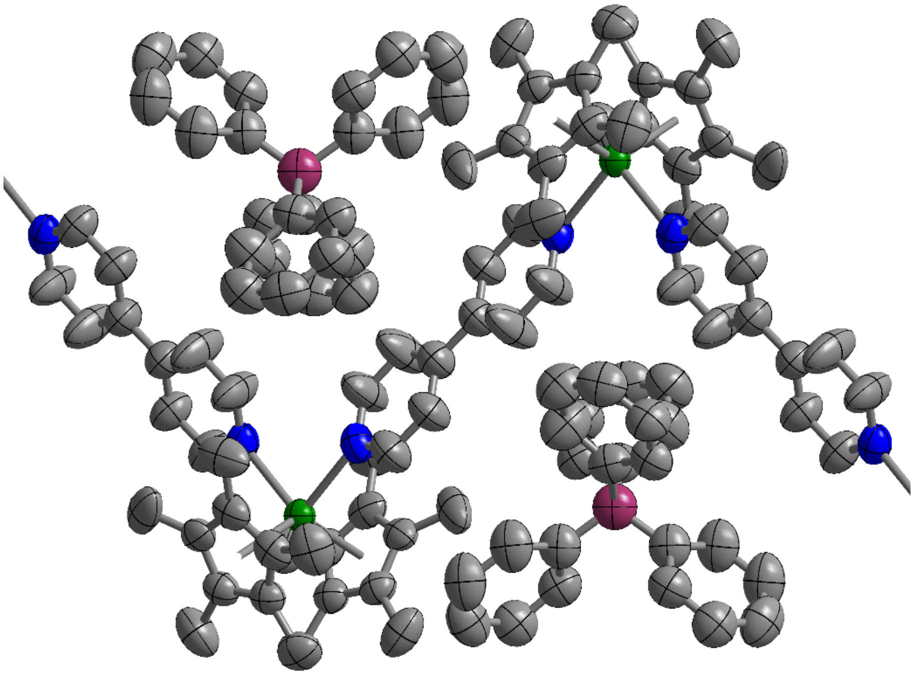

Figure S19. Structure of $\{[Cp^*_2Dy(bpy)][BPh_4]\}_n$, **3**, in a crystal of $\{[Cp^*_2Dy(bpy)][BPh_4]\}_n{\bullet}3(C_4H_8O)$ with thermal ellipsoids drawn at 50% probability level. Green, blue, grey, and purple spheres represent Dy, N, C, and B atoms, respectively. Hydrogen atoms and solvent molecules in the crystal lattice have been omitted for clarity.

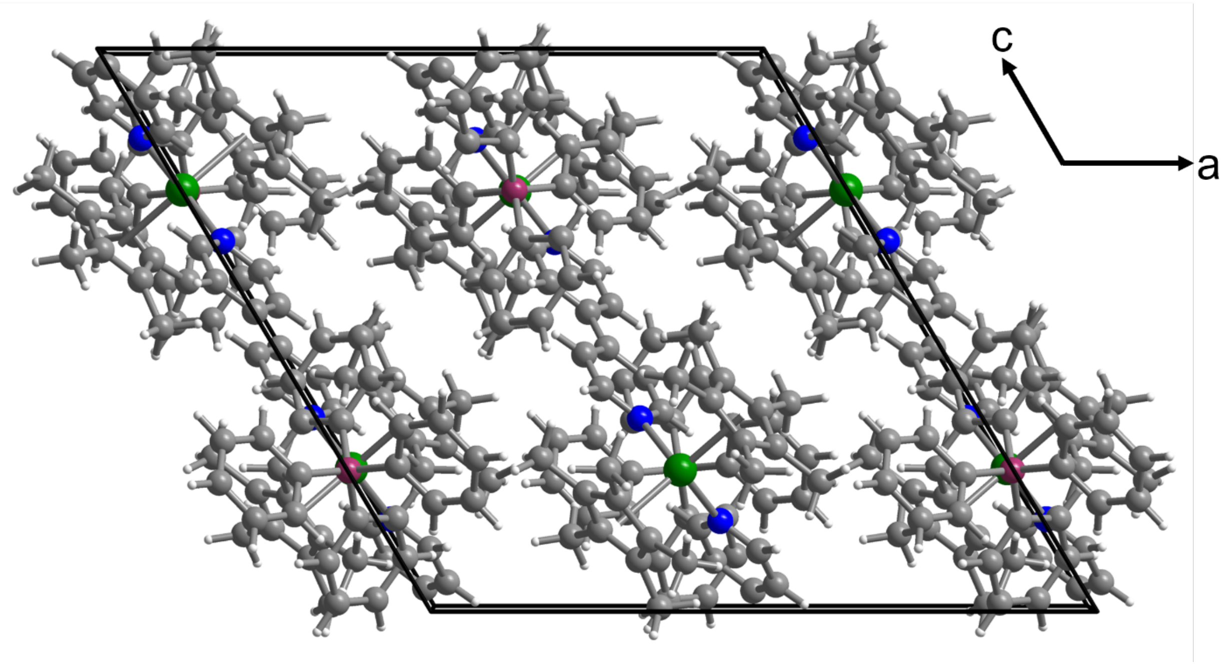

Figure S20. Unit cell depiction of {[Cp*$_2$Dy(bpy)][BPh$_4$]}$_n$, **3**, in a crystal of {[Cp*$_2$Dy(bpy)][BPh$_4$]}$_n$•3(C_4H_8O) along the *ac* plane. Green, blue, grey, purple, and white-grey spheres represent Dy, N, C, B, and H atoms, respectively. Solvent molecules in the crystal lattice have been omitted for clarity.

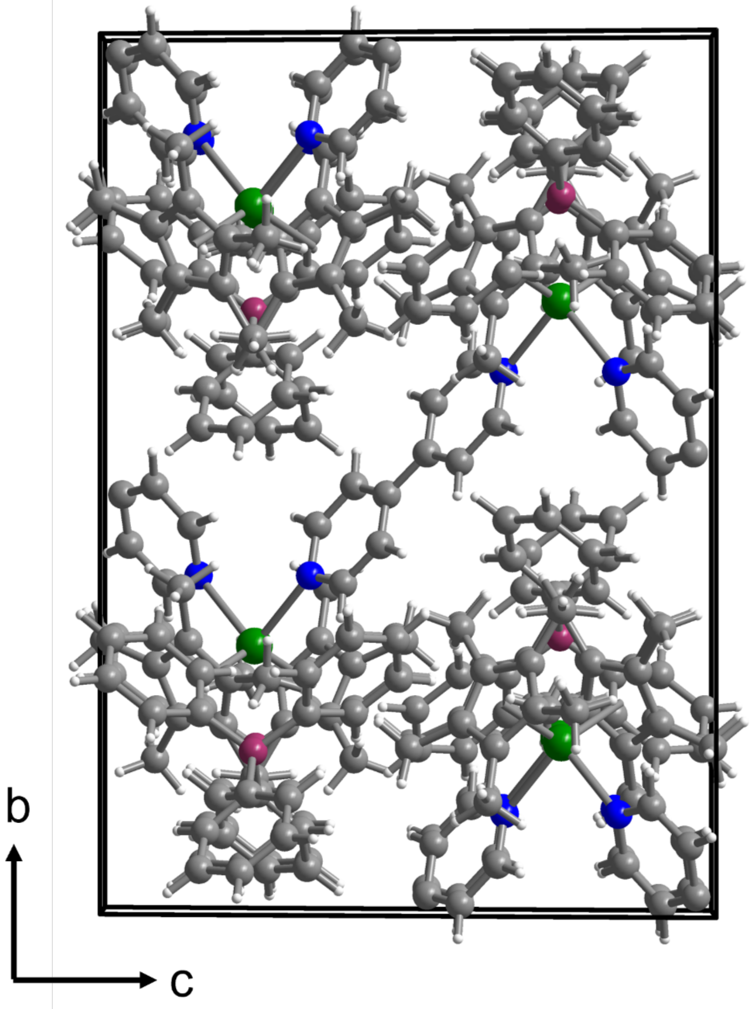

Figure S21. Unit cell depiction of {[Cp*$_2$Dy(bpy)][BPh$_4$]}$_n$, **3**, in a crystal of {[Cp*$_2$Dy(bpy)][BPh$_4$]}$_n$•3(C_4H_8O) along the *bc* plane. Green, blue, grey, purple, and white-grey spheres represent Dy, N, C, B, and H atoms, respectively. Solvent molecules in the crystal lattice have been omitted for clarity.

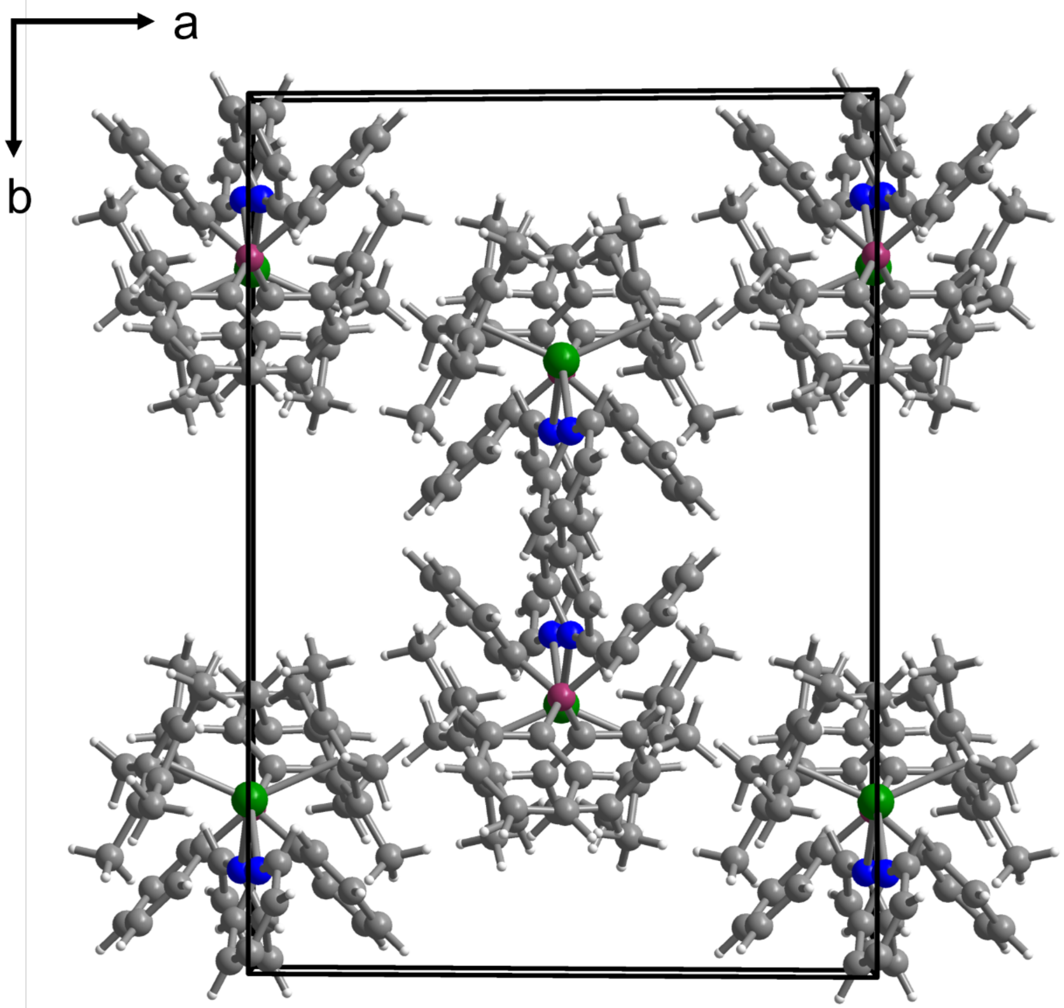

Figure S22. Unit cell depiction of {[Cp*$_2$Dy(bpy)][BPh$_4$]}$_n$, **3**, in a crystal of {[Cp*$_2$Dy(bpy)][BPh$_4$]}$_n$•3(C_4H_8O) along the *ab* plane. Green, blue, grey, purple, and white-grey spheres represent Dy, N, C, B, and H atoms, respectively. Solvent molecules in the crystal lattice have been omitted for clarity.

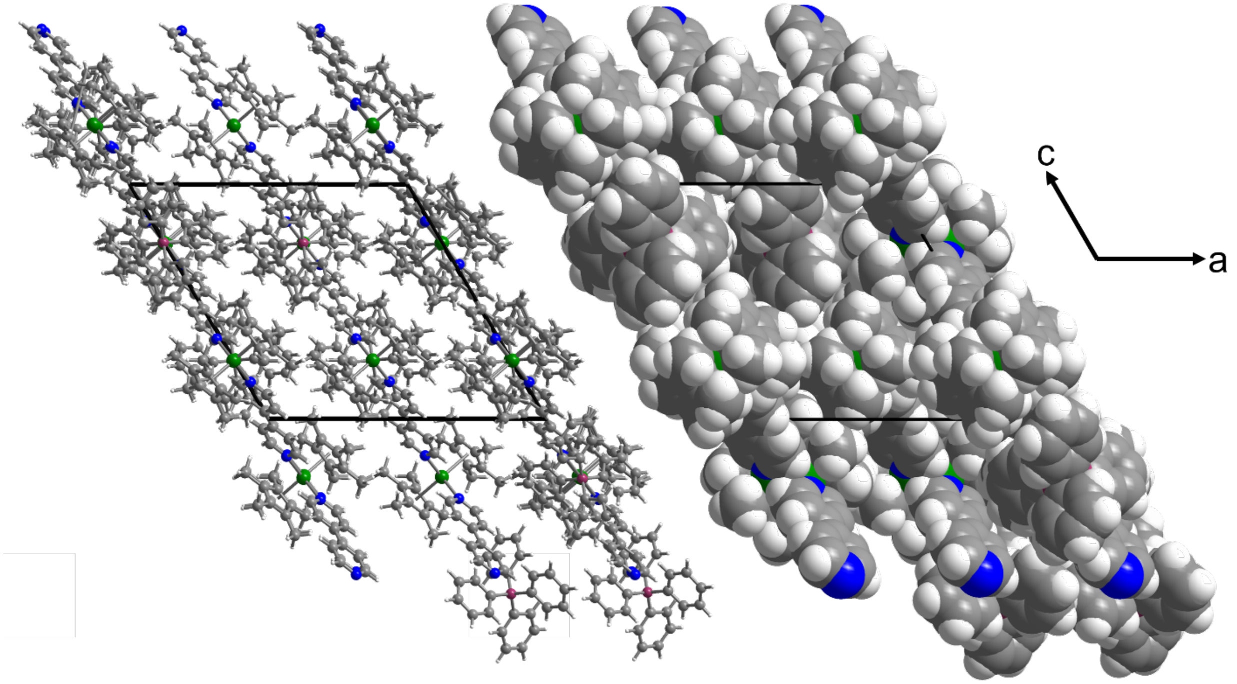

Figure S23. Crystal packing diagram of $\{[Cp^*_2Dy(bpy)][BPh_4]\}_n$, **3**, in a crystal of $\{[Cp^*_2Dy(bpy)][BPh_4]\}_n \cdot 3(C_4H_8O)$ along the *ac* plane with atoms displayed as standard spheres (left), and as a space-filling model (right). Green, blue, grey, purple, and white-grey spheres represent Dy, N, C, B, and H atoms, respectively. Solvent molecules in the crystal lattice have been omitted for clarity.

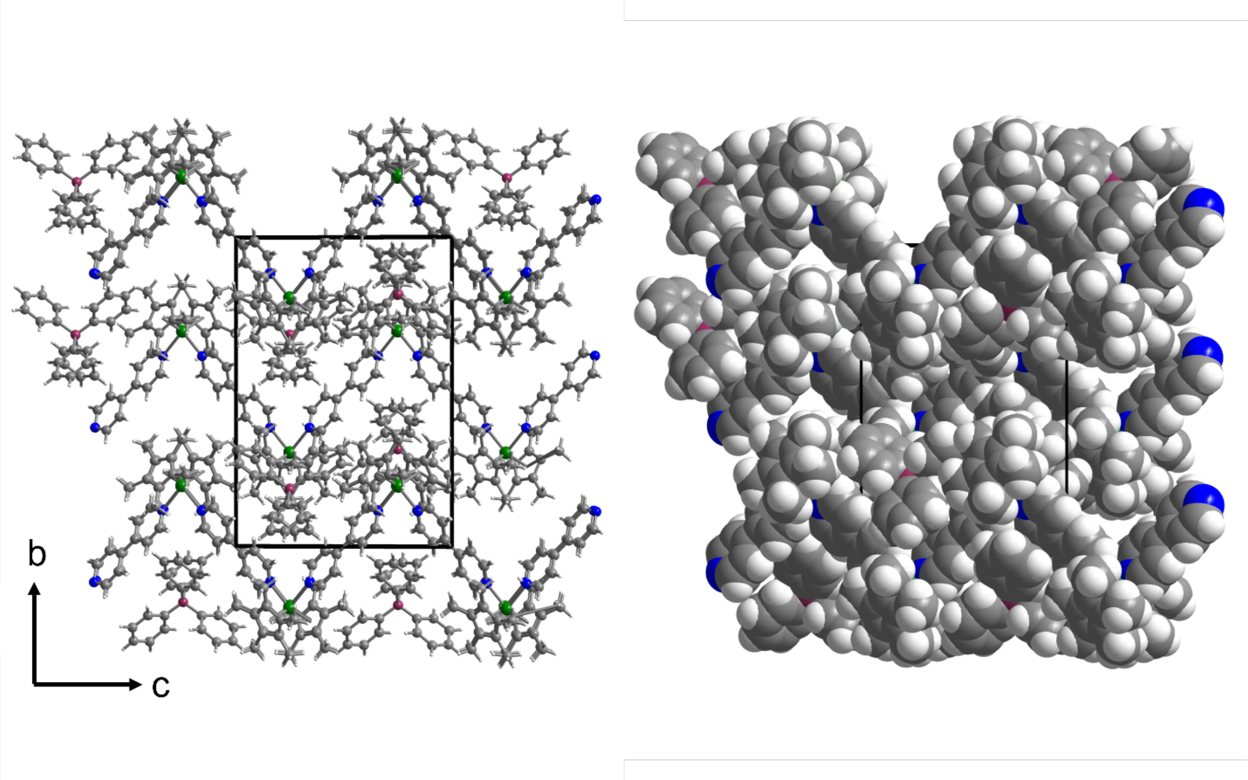

Figure S24. Unit cell depiction of {[Cp*$_2$Dy(bpy)][BPh$_4$]}$_n$, **3**, in a crystal of {[Cp*$_2$Dy(bpy)][BPh$_4$]}$_n$•3(C_4H_8O) along the *bc* plane with atoms displayed as standard spheres (left), and as a space-filling model (right). Green, blue, grey, purple, and white-grey spheres represent Dy, N, C, B, and H atoms, respectively. Solvent molecules in the crystal lattice have been omitted for clarity.

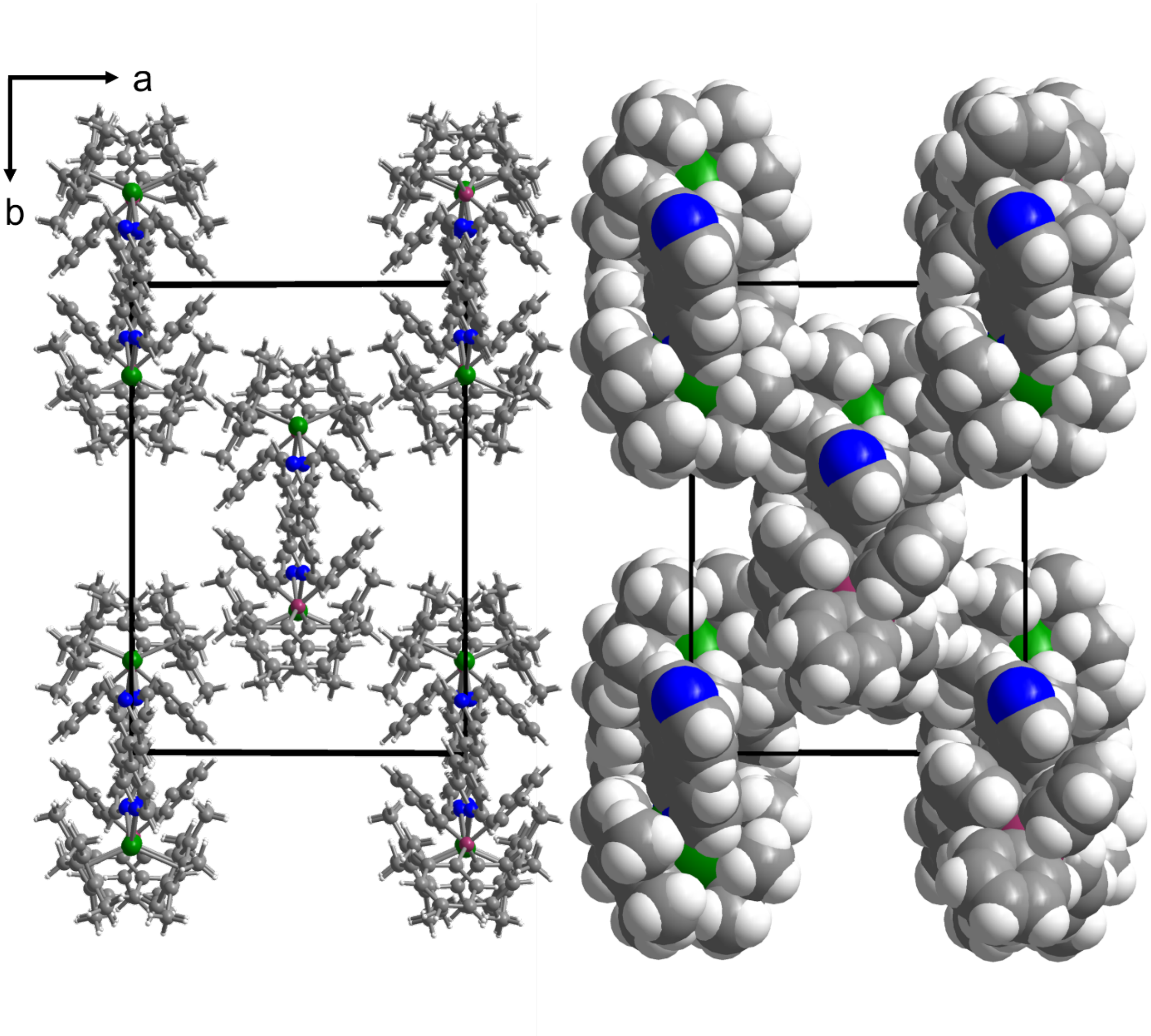

Figure S25. Crystal packing diagram of {[Cp*$_2$Dy(bpy)][BPh$_4$]}$_n$, **3**, in a crystal of {[Cp*$_2$Dy(bpy)][BPh$_4$]}$_n$•3(C_4H_8O) along the *ab* plane with atoms displayed as standard spheres (left), and as a space-filling model (right). Green, blue, grey, purple, and white-grey spheres represent Dy, N, C, B, and H atoms, respectively. Solvent molecules in the crystal lattice have been omitted for clarity.

Table S2. Metrical parameters for the crystallographic distances and angles in $\{[Cp^*_2Ln(bpy)][BPh_4]\}_n$, where Ln = Gd (**1**), Tb (**2**), and Dy (**3**).

	**Gd (1)**	**Tb (2)**	**Dy (3)**
	Distances (Å)		
Ln–N	2.488(6)	2.447(7)	2.444(5)
Ln–C_{Cp^*}	2.676(10)<br>2.743(24)	2.633(12)<br>2.747(26)	2.649(19)<br>2.711(21)
Ln–Cnt	2.423	2.395	2.392
Ln–B	8.151(10)<br>8.768(1)	8.130(20)<br>8.668(1)	8.146(1)<br>8.665(1)
Ln···$Ln_{intrachain}$	12.209(1)<br>17.522(1)	12.111(1)<br>17.323(1)	12.063(1)<br>17.323(1)
Ln···$Ln_{interchain}$	9.099(1)<br>13.827(1)<br>15.244(1)	8.932(1)<br>13.851(1)<br>15.354(1)	8.949(1)<br>13.851(1)<br>15.364(1)
B···B	11.729(12)	11.649(20)	11.741(12)
	Angles (°)		
N–Ln–N	91.1(2)	91.5(2)	90.6(2)
Cnt–Ln–Cnt	131.7	132.3	133.0
Ln···Ln···Ln	91.7(1)	91.3(0)	91.8(1)
Ln···Ln···Ln···Ln	180.0(1)	180.0(1)	180.0(1)
bpy···bpy	48.7(2)	46.2(2)	45.1(2)
Ionic Radius (Å)‡	1.053	1.04	1.027

‡Ionic radius for eight-coordinate Ln^{III} ion.[1]

2 IR Spectroscopy

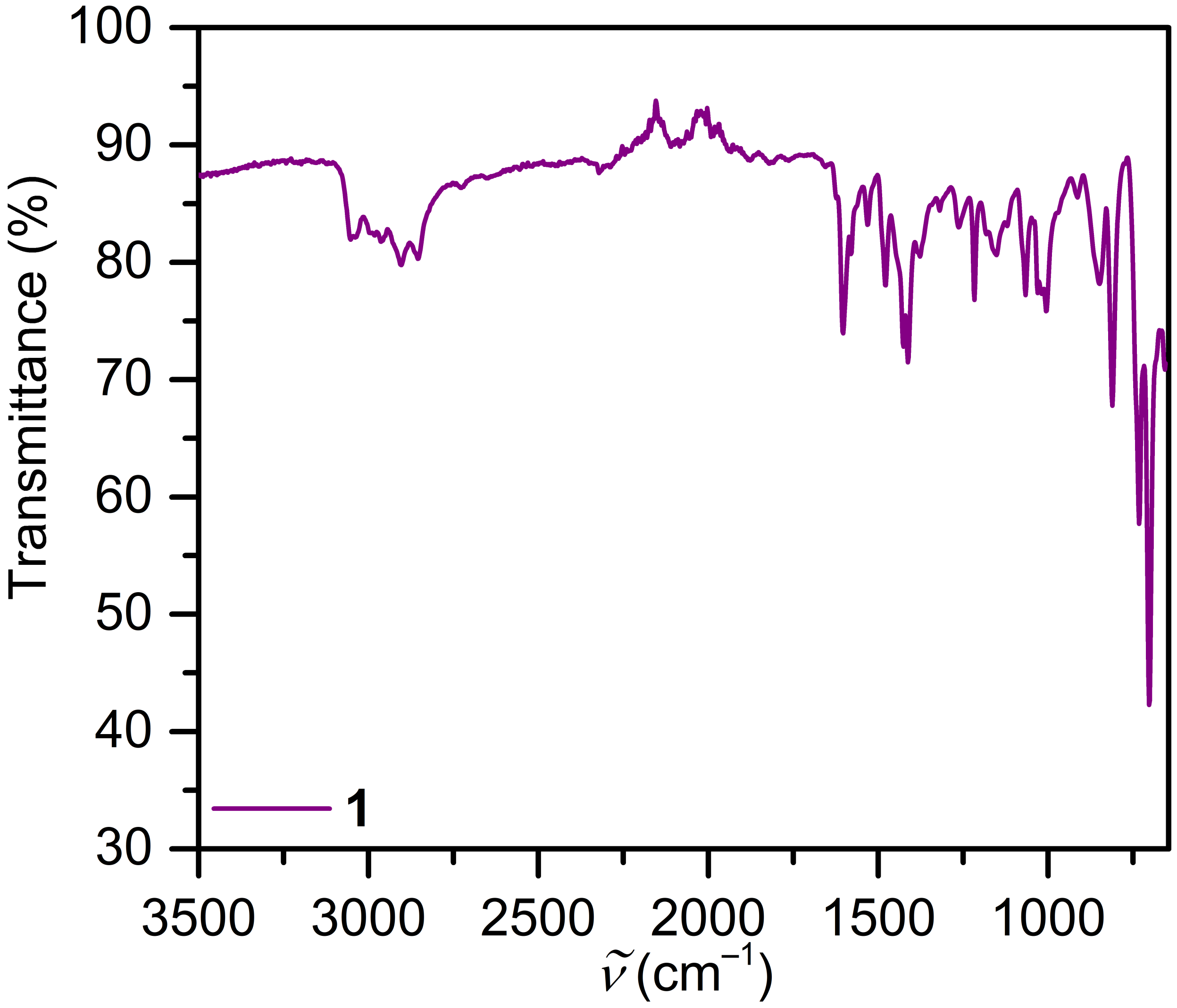

Figure S26. Experimental FTIR spectrum of $\{[Cp^*_2Gd(bpy)][BPh_4]\}_n$, **1** (dark purple line), measured on crushed crystalline solids in a nitrogen-filled glovebox.

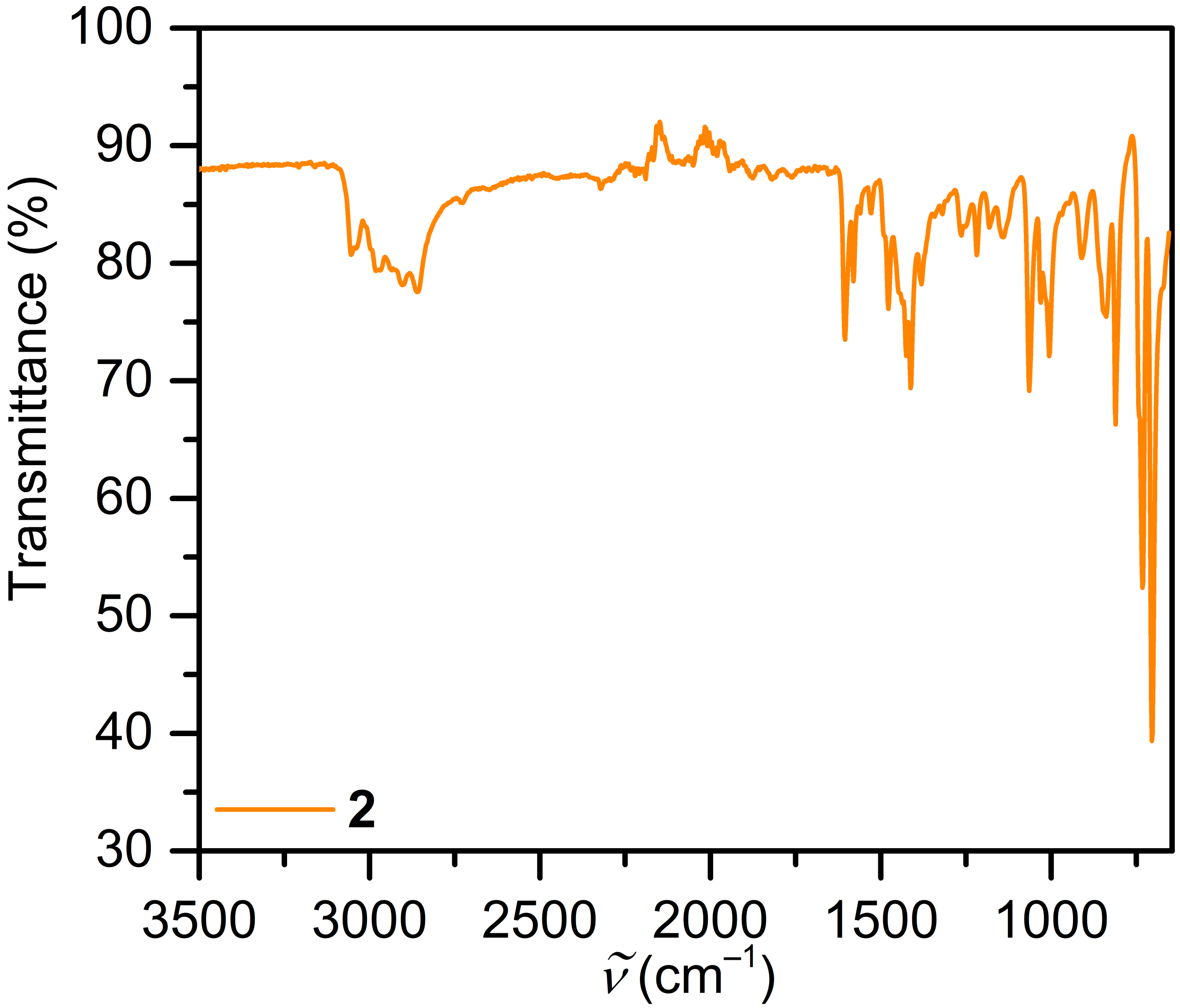

Figure S27. Experimental FTIR spectrum of {[Cp*$_2$Tb(bpy)][BPh$_4$]}$_n$, **2** (orange line), measured on crushed crystalline solids in a nitrogen-filled glovebox.

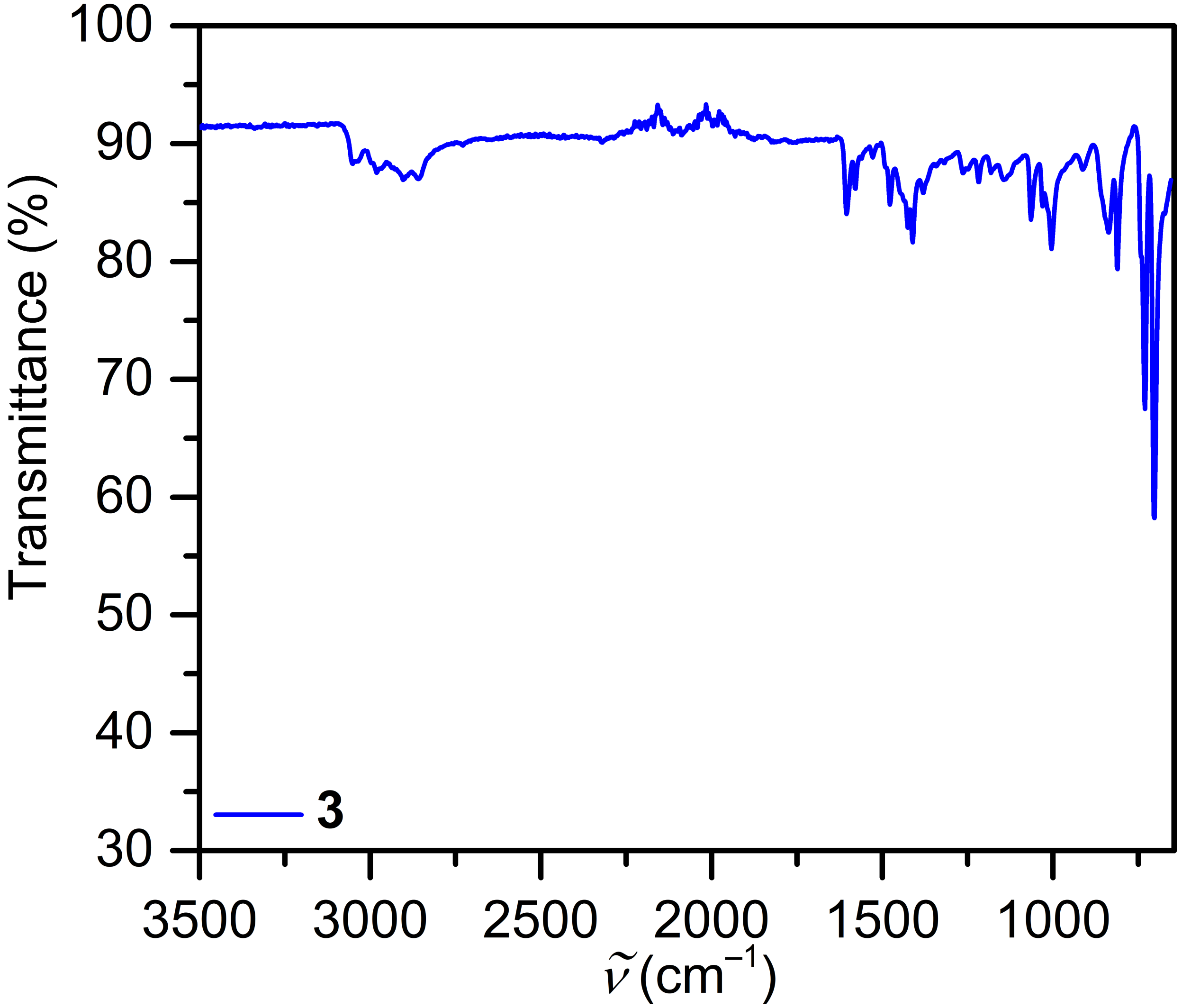

Figure S28. Experimental FTIR spectrum of $\{[Cp^*_2Dy(bpy)][BPh_4]\}_n$, **3** (blue line), measured on crushed crystalline solids in a nitrogen-filled glovebox.

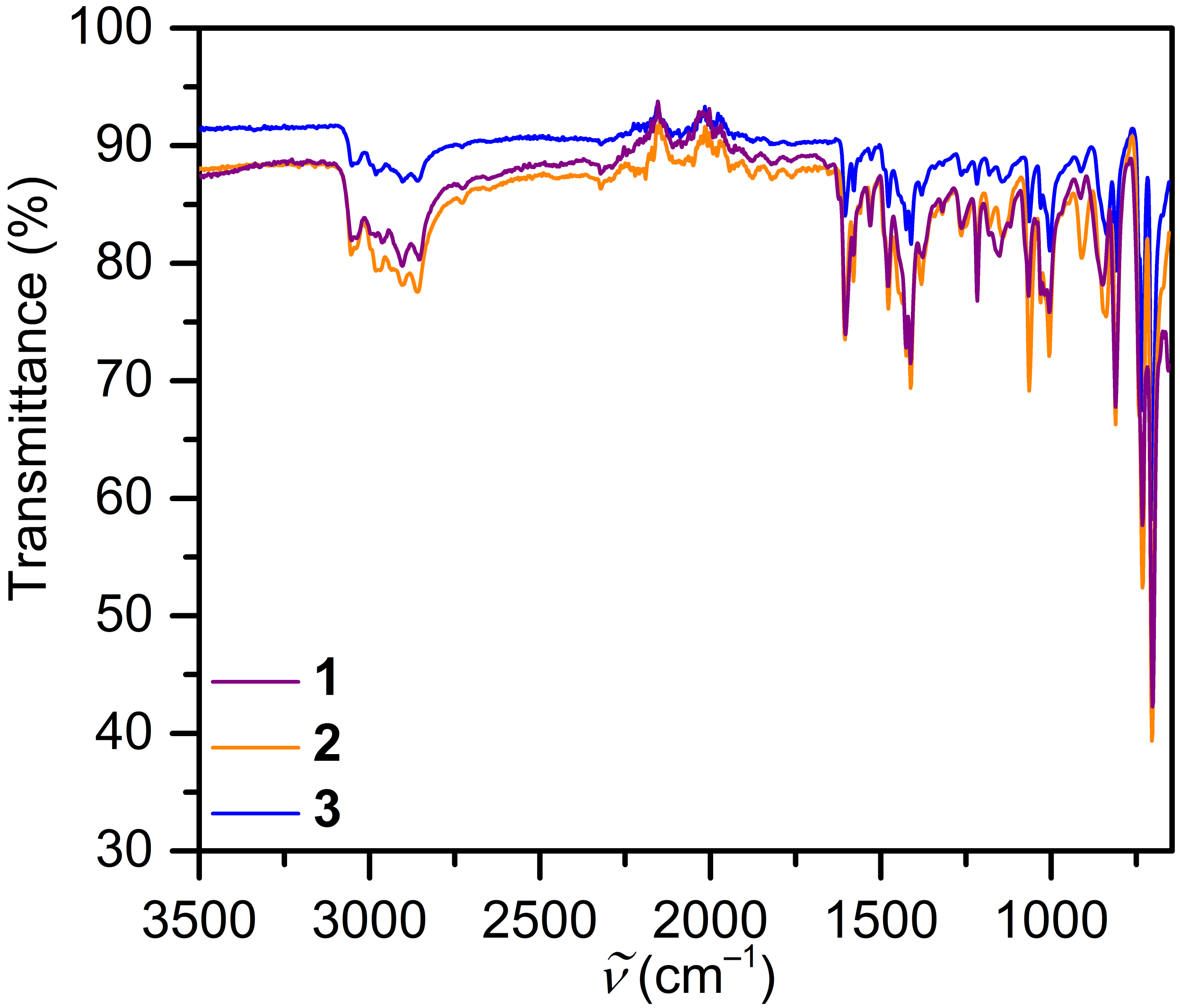

Figure S29. Experimental FTIR spectra of $\{[Cp^*_2Ln(bpy)][BPh_4]\}_n$ (where Ln = Gd (**1**, dark purple line), Tb (**2**, orange line), Dy (**3**, blue line)), measured on crushed crystalline solids in a nitrogen-filled glovebox.

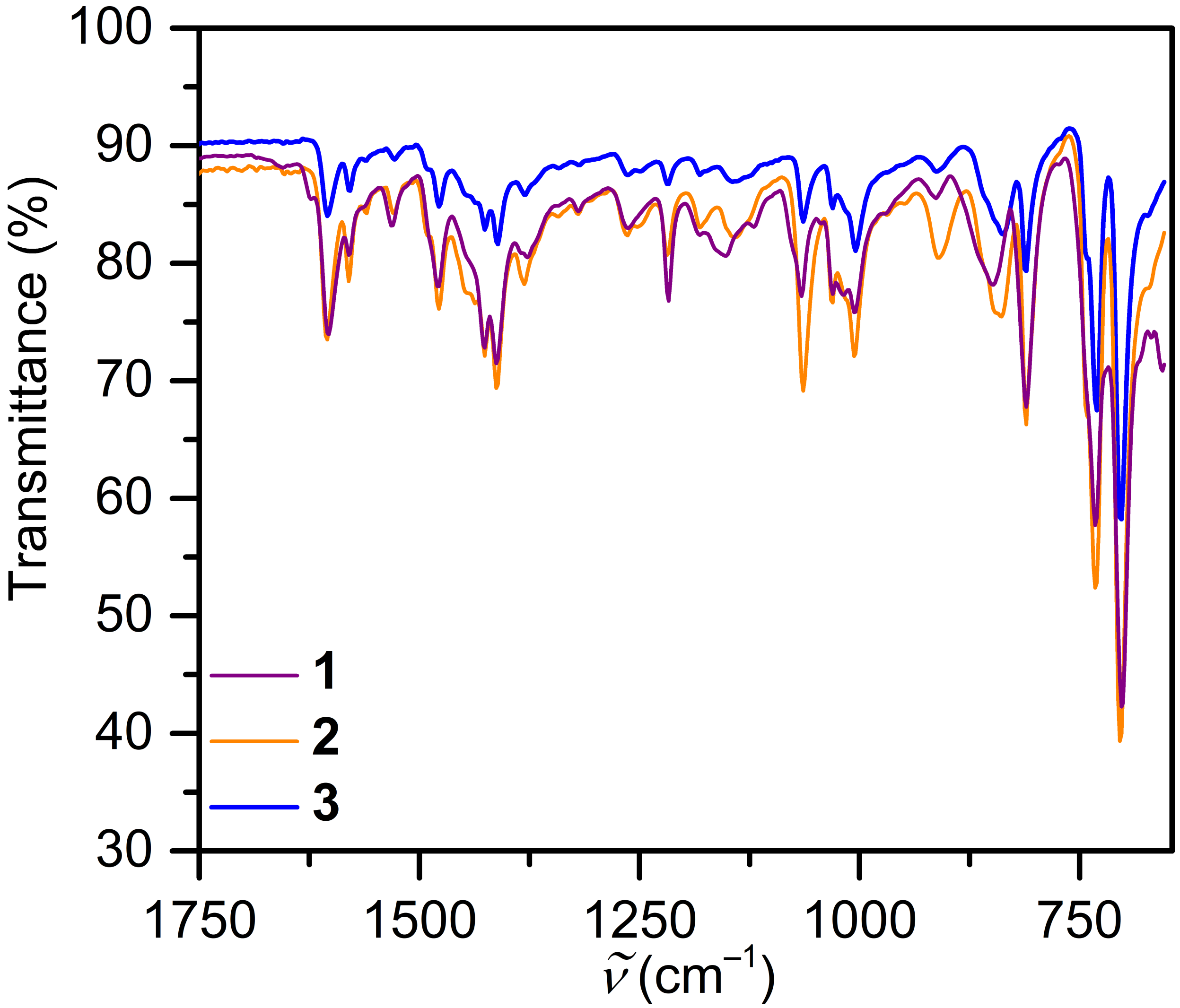

Figure S30. Magnification of the experimental FTIR spectra of $\{[Cp^*_2Ln(bpy)][BPh_4]\}_n$ (where Ln = Gd (**1**, dark purple line), Tb (**2**, orange line), Dy (**3**, blue line)), measured on crushed crystalline solids in a nitrogen-filled glovebox.

3 Magnetic Data

Table S3. Selected mononuclear dysprosium metallocene complexes bearing neutral coordinating ligands or weakly coordinating anions.

Chemical Formula	U_{eff} (cm^{-1})	T_b (K)	M···M[a] (Å)	Ref
$[(Cp^*_2Dy)_2(\eta^2\text{-}Ph_2BPh_2)]$	312 (H_{dc} = 0 Oe) 314 (H_{dc} = 0 Oe)	5.3	9.802(1)	2
$[Cp^*_2Dy(NH_3)_2][BPh_4]$	546(6) (H_{dc} = 0 Oe) 609(44) (H_{dc} = 1400 Oe)	5.8	9.597(1)	3
$[Cp^{ttt}DyCp^*(PhF\text{-}\kappa^1\text{-}F)][Al\{OC(CF_3)_3\}_4]$	1100(9) (H_{dc} = 0 Oe)	22	13.604(1)	4
$[Cp^{ttt}DyCp^*(PhCl\text{-}\kappa^1\text{-}Cl)][Al\{OC(CF_3)_3\}_4]$	1125(12) (H_{dc} = 0 Oe)	24	13.650(1)	4
$[Cp^{ttt}DyCp^*(PhBr\text{-}\kappa^1\text{-}Br)][Al\{OC(CF_3)_3\}_4]$	1182(9) (H_{dc} = 0 Oe)	24	11.412(1)	4
$[Cp^*_2Dy(PhF\text{-}\kappa^1\text{-}F)_2][Al\{OC(CF_3)_3\}_4]$	930(6) (H_{dc} = 0 Oe)	8	8.725(1)	5
$[Cp^*_2Dy(PhCl\text{-}\kappa^1\text{-}Cl)_2][Al\{OC(CF_3)_3\}_4]$	921(6) (H_{dc} = 0 Oe)	10	13.143(1)	5
$[Cp^*_2Dy(PhF_2\text{-}\kappa^2\text{-}F,F)(PhF_2\text{-}\kappa^1\text{-}F)]$ $[Al\{OC(CF_3)_3\}_4]$	776(11) (H_{dc} = 0 Oe)	4	9.698(1)	5

‡Cp^{ttt} = $C_5H_2(CMe_3)_2$; Cp* = C_5Me_5.

Table S4. Selected multinuclear dysprosium metallocene complexes bearing diamagnetic bridging-ligands.

Chemical Formula	U_{eff} (cm^{-1})	T_b (K)	M···M[a] (Å)	Ref
$[(Cp^*{}_2Dy)_2(\mu\text{-}n^2\text{:}n^2\text{-}Ph_2BPh_2][Al\{OC(CF_3)_3\}_4]$	330 (H_{dc} = 0 Oe) 340 (H_{dc} = 1600 Oe)	6.5	9.07	6
$[(Cp^{ttt}DyCp^{Me4t})_2(\mu\text{:}k^2\text{:}k^2\text{:-}BH_4)][B(C_6F_5)_4]$	533(18) (H_{dc} = 1000 Oe)	2	5.710(1)	7
$[(Cp'_2Dy)(\mu\text{-}F)]_3$	60 (H_{dc} = 0 Oe) 66 (H_{dc} = 1000 Oe)	–	4.29	8
$[(Cp'_2Dy)(\mu\text{-}Cl)]_2$	106 (H_{dc} = 0 Oe)	7	4.07	9
$[(Cp'_2Dy)(\mu\text{-}Br)]_2$	139 (H_{dc} = 0 Oe)	8	4.22	9
$[(Cp'_2Dy\ )(\mu\text{-}I)]_2$	341 (H_{dc} = 0 Oe)	10	4.47	9
$[(Cp'_2Dy)\{\mu\text{-}As(H)Mes\}]_3$	256(5) (H_{dc} = 0 Oe)	1.8	5.443(1)	10
$[Li(THF)_4]_2[(Cp'_2Dy)_3(\mu^3\text{-}AsMes)_3Li]$	23(2) (H_{dc} = 0 Oe)	1.8	5.257(1)	10
$[(Cp'_2Dy)\{\mu\text{-}SeMes\}]_3$	252(4) (H_{dc} = 0 Oe) 285(4) (H_{dc} = 1000 Oe)	1.8	5.291(1)	10
$[(Cp^*{}_2Dy)_2(\mu\text{-}Bbim)]$	182.1(1)	5	6.203(3)	11
$[\{Cp^*{}_2Dy(\mu\text{-}Me_3AlNEt_3)\}_2][Al\{OC(CF_3)_3\}_4]_2$	860(60) (H_{dc} = 0 Oe)	12	8.712(1)	12

‡Cp^{ttt} = $C_5H_2(CMe_3)_2$; Cp* = C_5Me_5; Cp′ = $C_5H_4SiMe_3$; Mes = 2,4,6-$C_6H_2Me_3$.

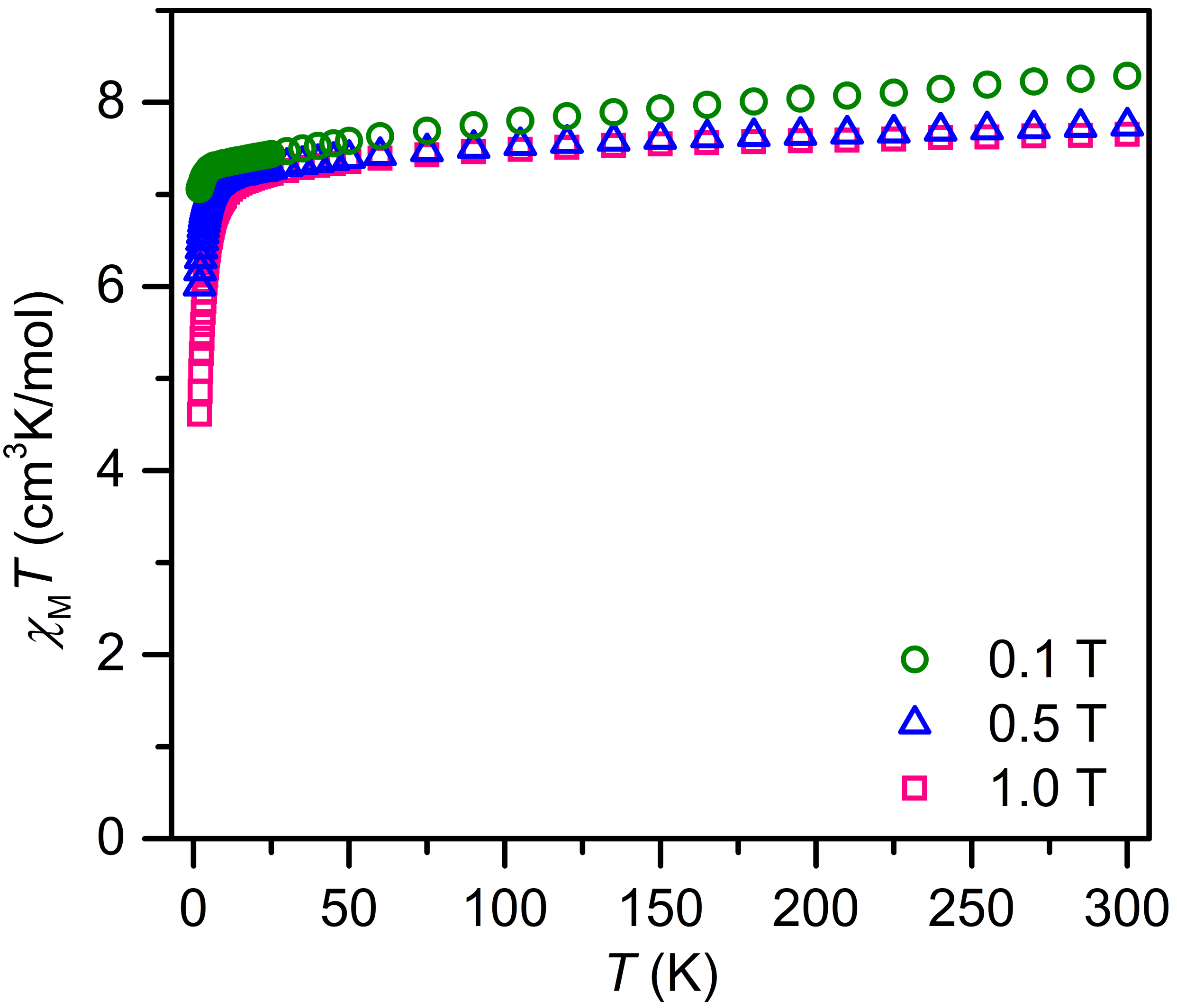

Figure S31. Variable-temperature dc magnetic susceptibility data for a restrained polycrystalline sample of $\{[Cp^*_2Gd(bpy)][BPh_4]\}_n$, **1**, collected under 0.1 T (green circles), 0.5 T (blue triangles), and 1.0 T (pink squares) applied dc fields.

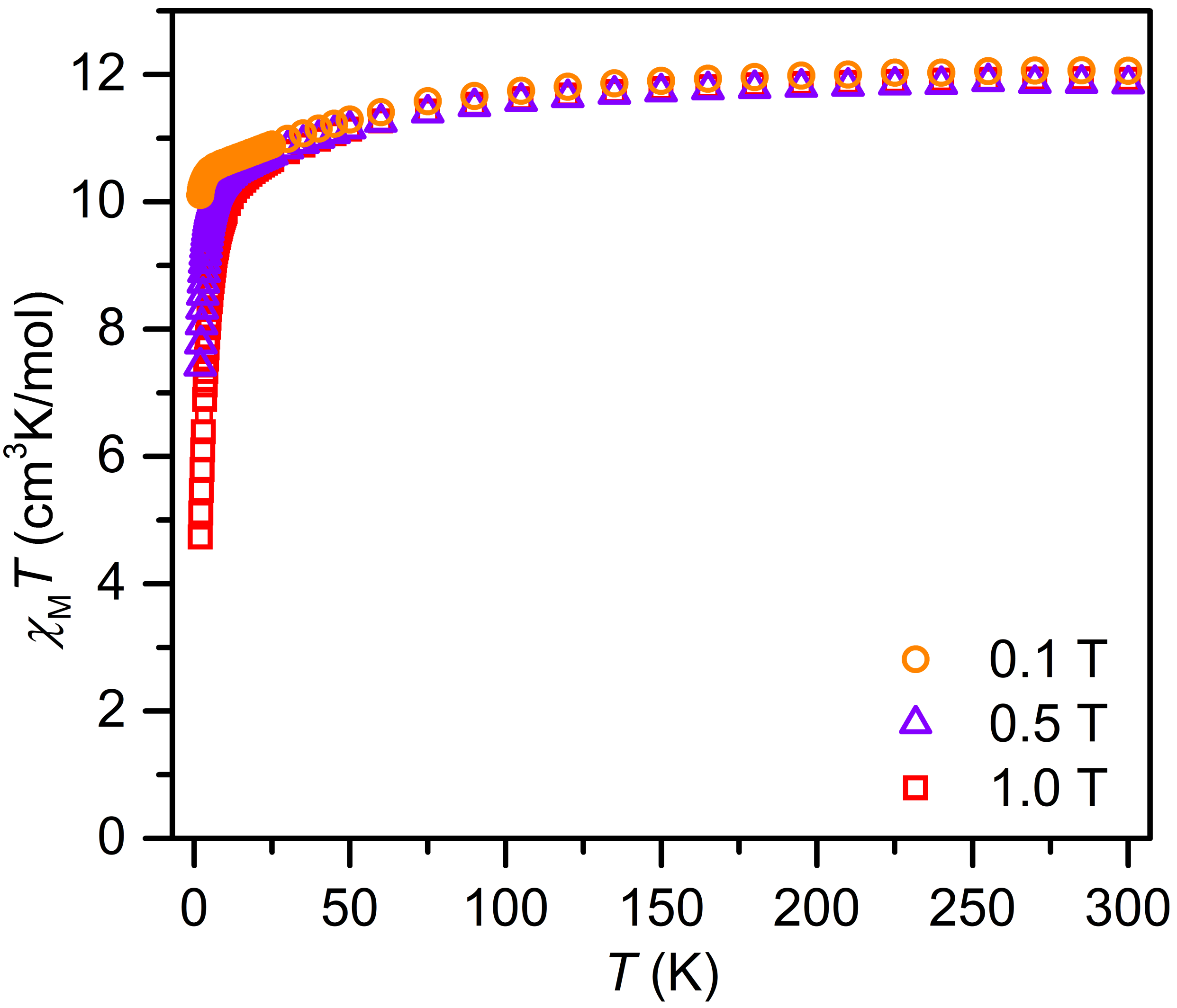

Figure S32. Variable-temperature dc magnetic susceptibility data for a restrained polycrystalline sample of $\{[Cp^*_2Tb(bpy)][BPh_4]\}_n$, **2**, collected under 0.1 T (orange circles), 0.5 T (purple triangles), and 1.0 T (red squares) applied dc fields.

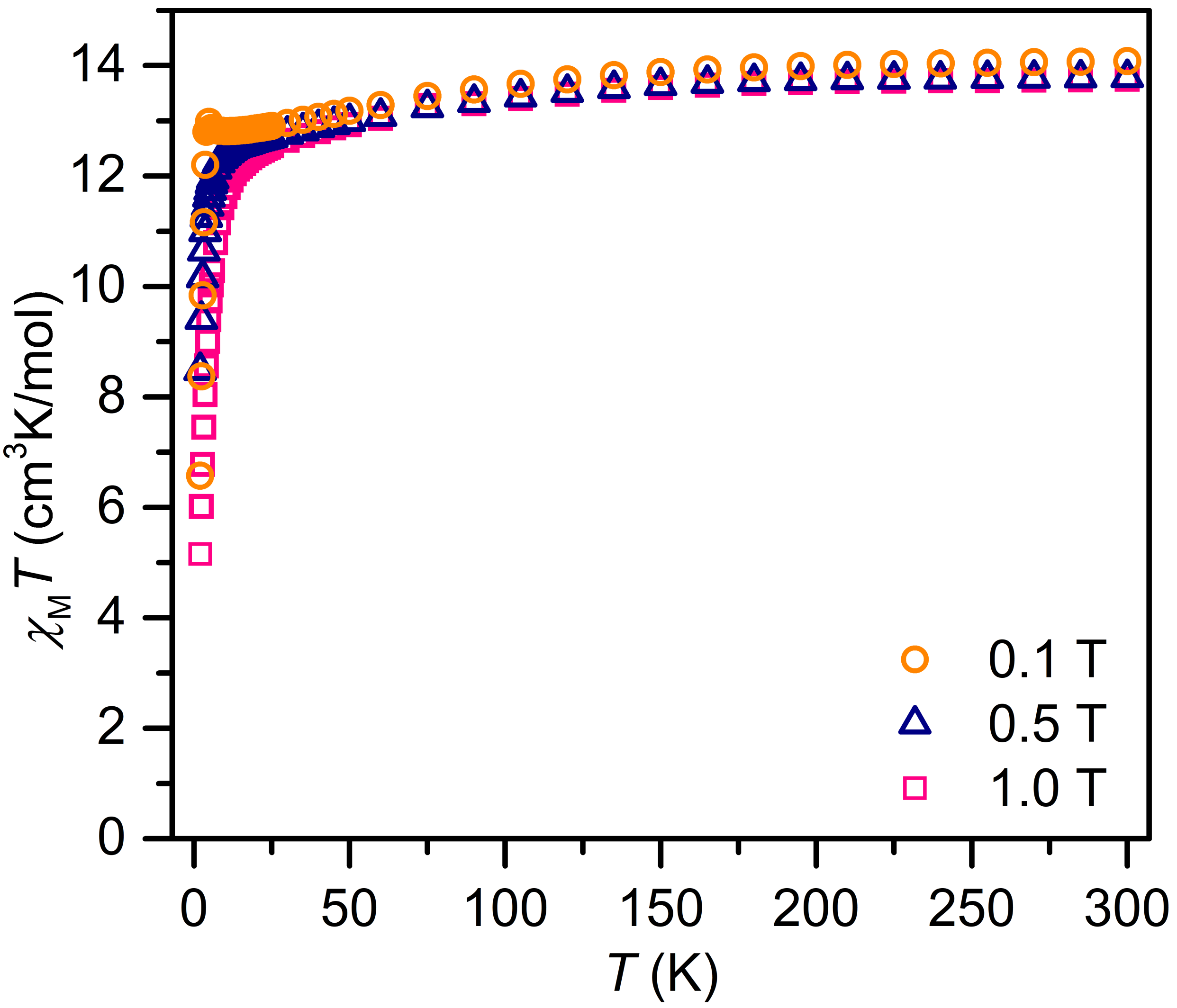

Figure S33. Variable-temperature dc magnetic susceptibility data for a restrained polycrystalline sample of $\{[Cp^*_2Dy(bpy)][BPh_4]\}_n$, **3**, collected under 0.1 T (orange circles), 0.5 T (dark blue triangles), and 1.0 T (pink squares) applied dc fields.

.

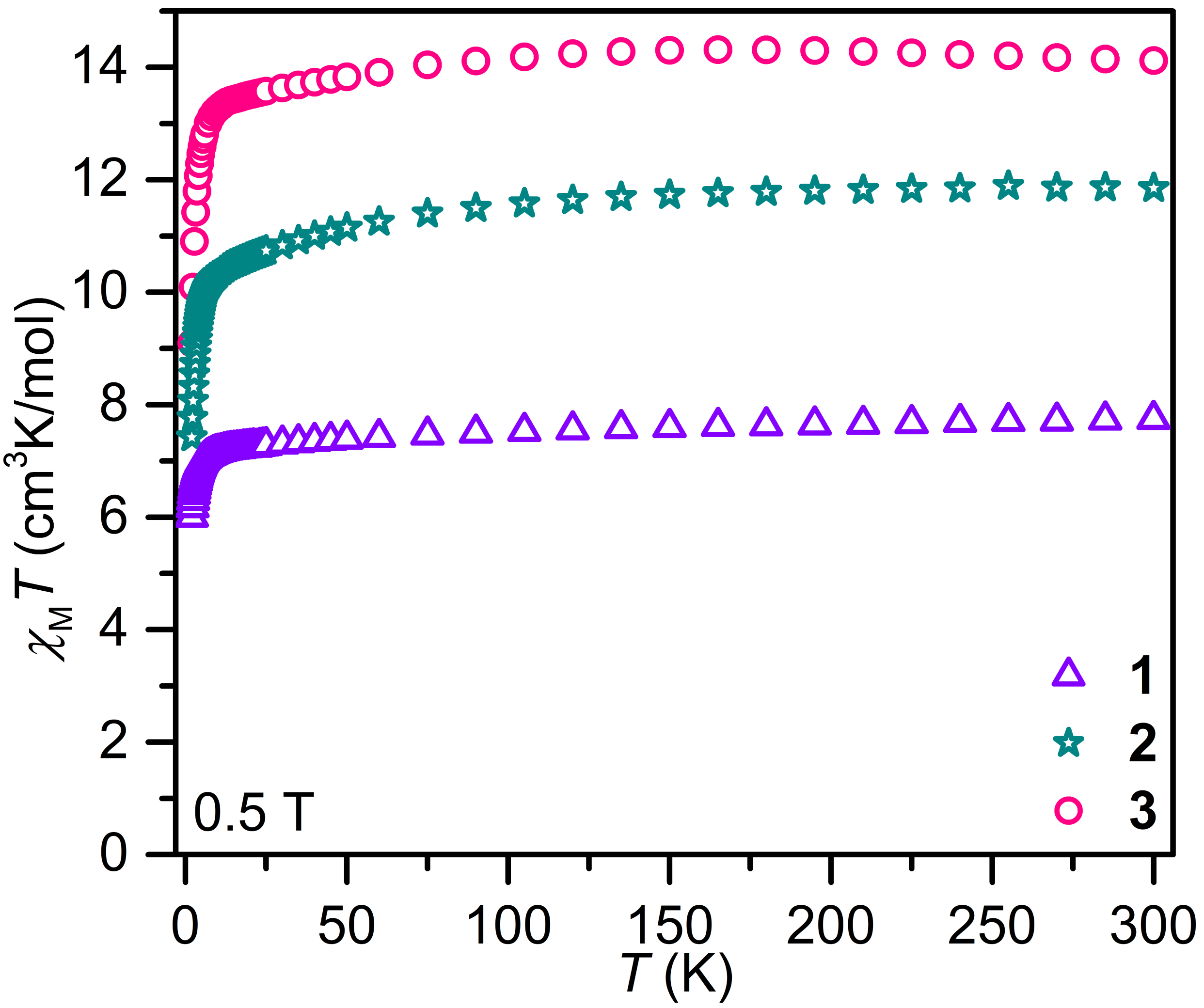

Figure S34. Variable-temperature dc magnetic susceptibility data for a restrained polycrystalline sample of $\{[Cp^*_2Ln(bpy)][BPh_4]\}_n$ (where Ln = Gd (**1**, purple triangles), Tb (**2**, turquoise stars), Dy (**3**, pink circles)), collected under a 0.5 T applied dc field.

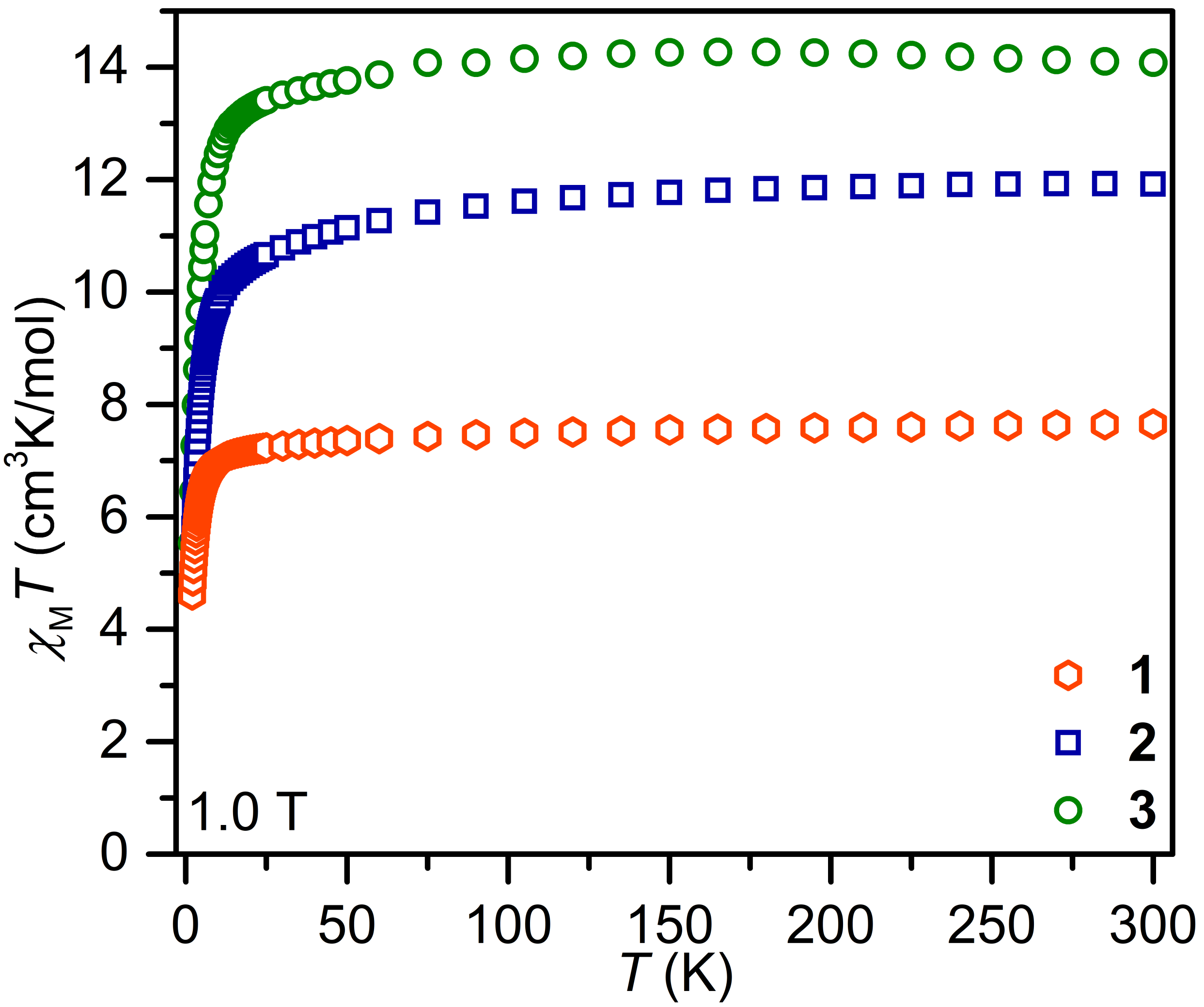

Figure S35. Variable-temperature dc magnetic susceptibility data for a restrained polycrystalline sample of $\{[Cp^*_2Ln(bpy)][BPh_4]\}_n$ (where Ln = Gd (**1**, orange hexagons), Tb (**2**, dark blue squares), Dy (**3**, green circles)), collected under a 1.0 T applied dc field.

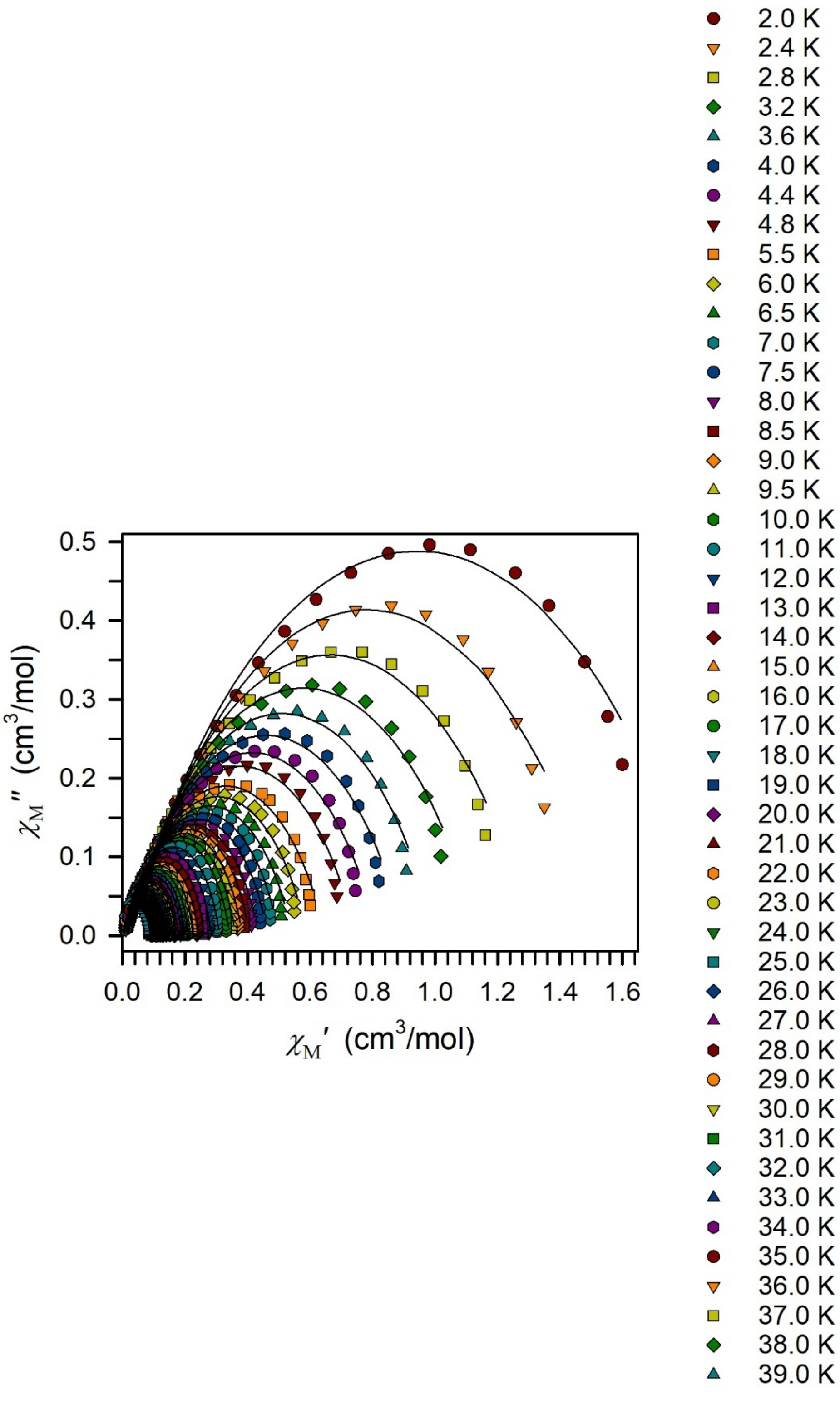

Figure S36. Cole-Cole (Argand) plots for ac susceptibility collected from 2 to 39 K under zero applied dc field for $\{[Cp^*_2Dy(bpy)][BPh_4]\}_n$, **3**. Symbols represent the experimental data points and the points representing the fits are connected by solid black lines.

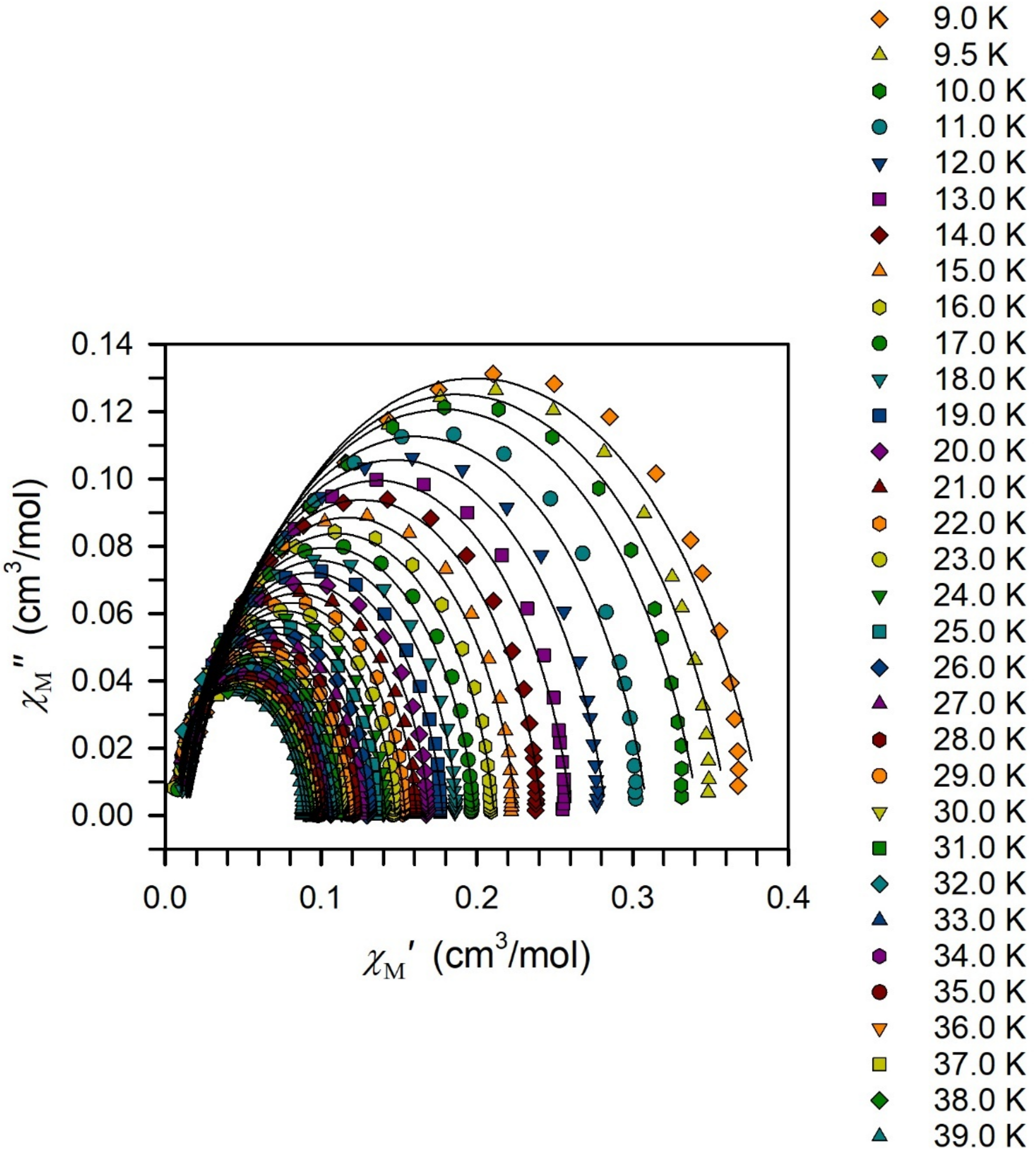

Figure S37. Cole-Cole (Argand) plots for ac susceptibility collected under zero applied dc field for $\{[Cp^*_2Dy(bpy)][BPh_4]\}_n$, **3**, shown for the temperature range 9 to 39 K. Symbols represent the experimental data points and the points representing the fits are connected by solid black lines.

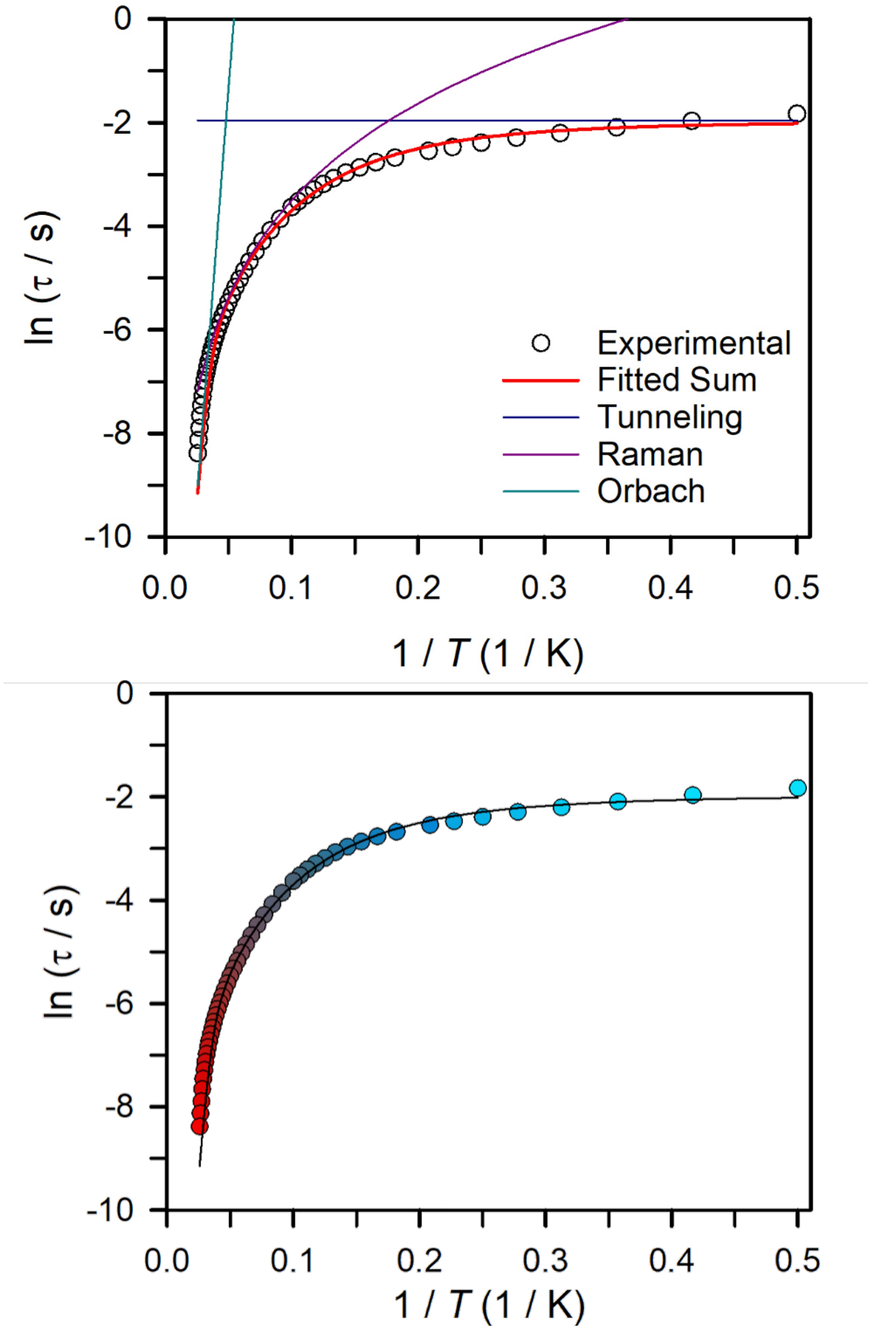

Figure S38. (Top) Individual contributions of the multiple magnetic relaxation pathways to the Arrhenius plot of {[Cp*$_2$Dy(bpy)][BPh$_4$]}$_n$, **3**, at 0 Oe. Individual parameters used to calculate the contributions are U_{eff} = 218.6 cm^{-1}, τ_0 = 3.88 x 10^{-8} s, n = 2.704, C = 6.56 x 10^{-2} s^{-1} K^{-n}, τ_{QTM} = 0.1411 s. (Bottom) Plot of natural log of the relaxation time versus the inverse temperature (temperature range 2 to 39 K) for **2**. Pale blue to red circles represent data extracted from ac magnetic susceptibility measurements. The black line represents a fit to Orbach relaxation process, a Raman relaxation process, and a quantum tunnelling pathway.

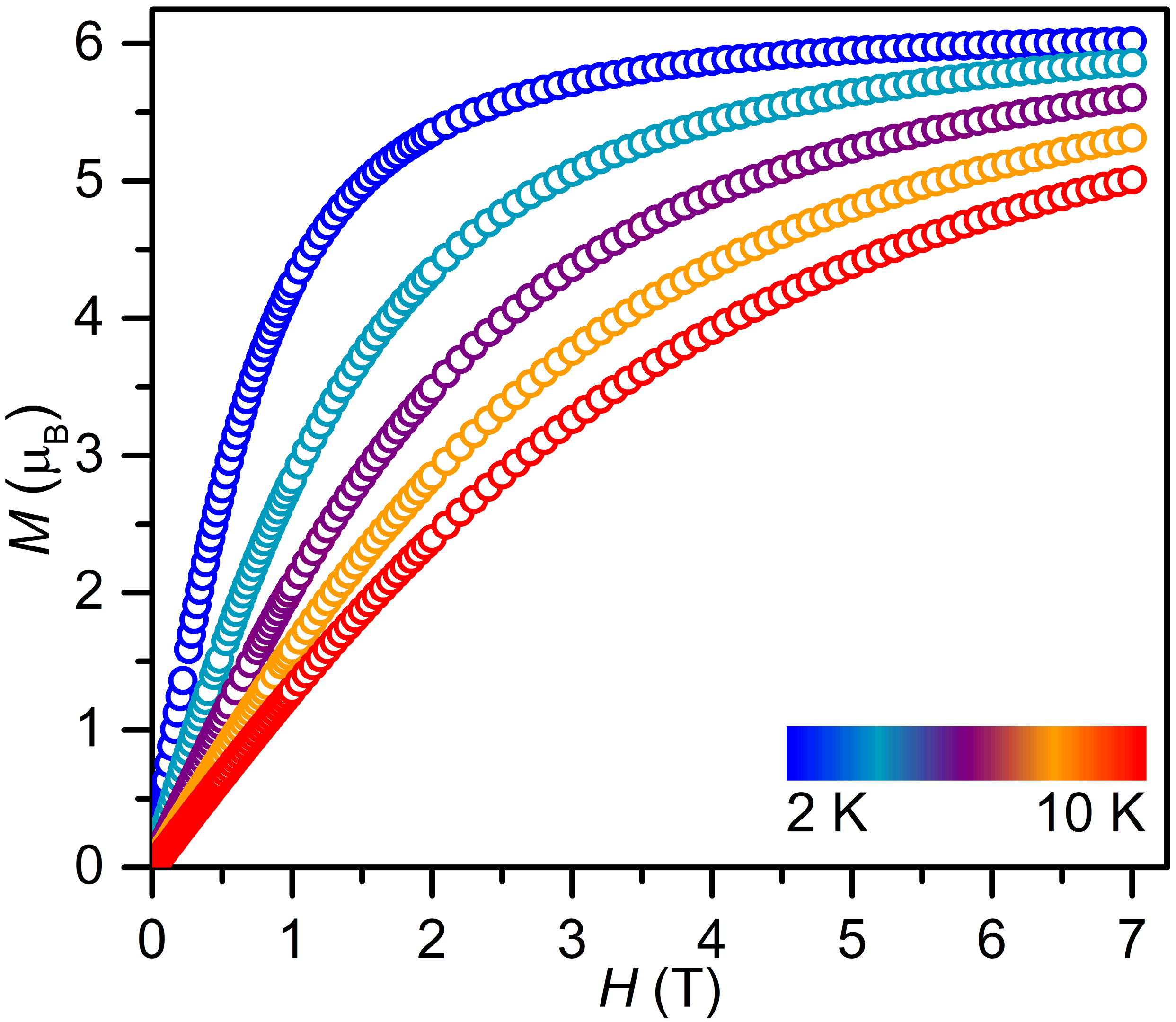

Figure S39. Variable-temperature field-dependent magnetisation curves recorded for $\{[Cp^*_2Gd(bpy)][BPh_4]\}_n$, **1**. Measurements were carried out between 0 and 7 T at 2, 4, 6, 8, and 10 K.

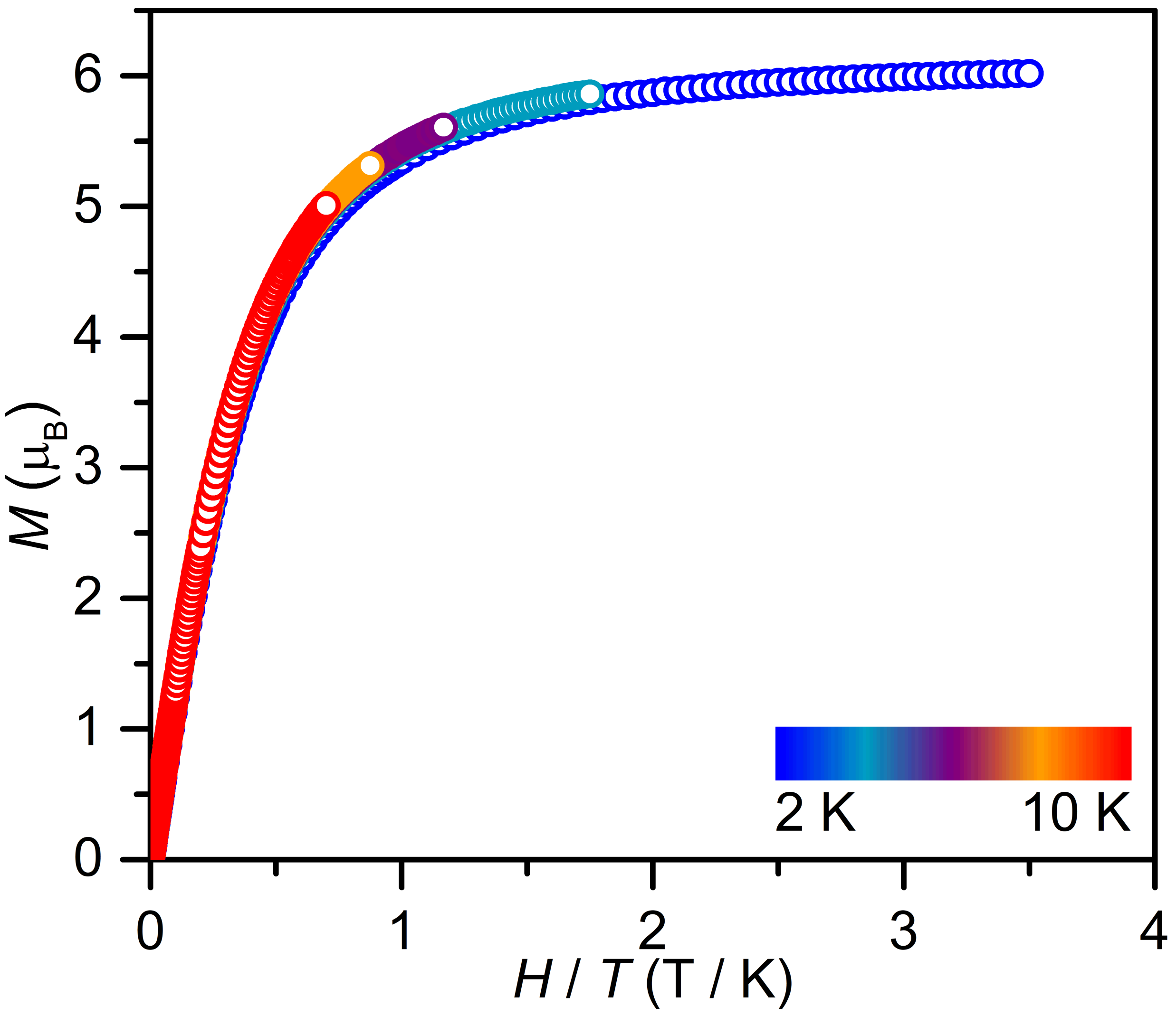

Figure S40. Reduced magnetisation data of $\{[Cp^*_2Gd(bpy)][BPh_4]\}_n$, **1**, collected from 0 to 7 T at 2, 4, 6, 8, and 10 K.

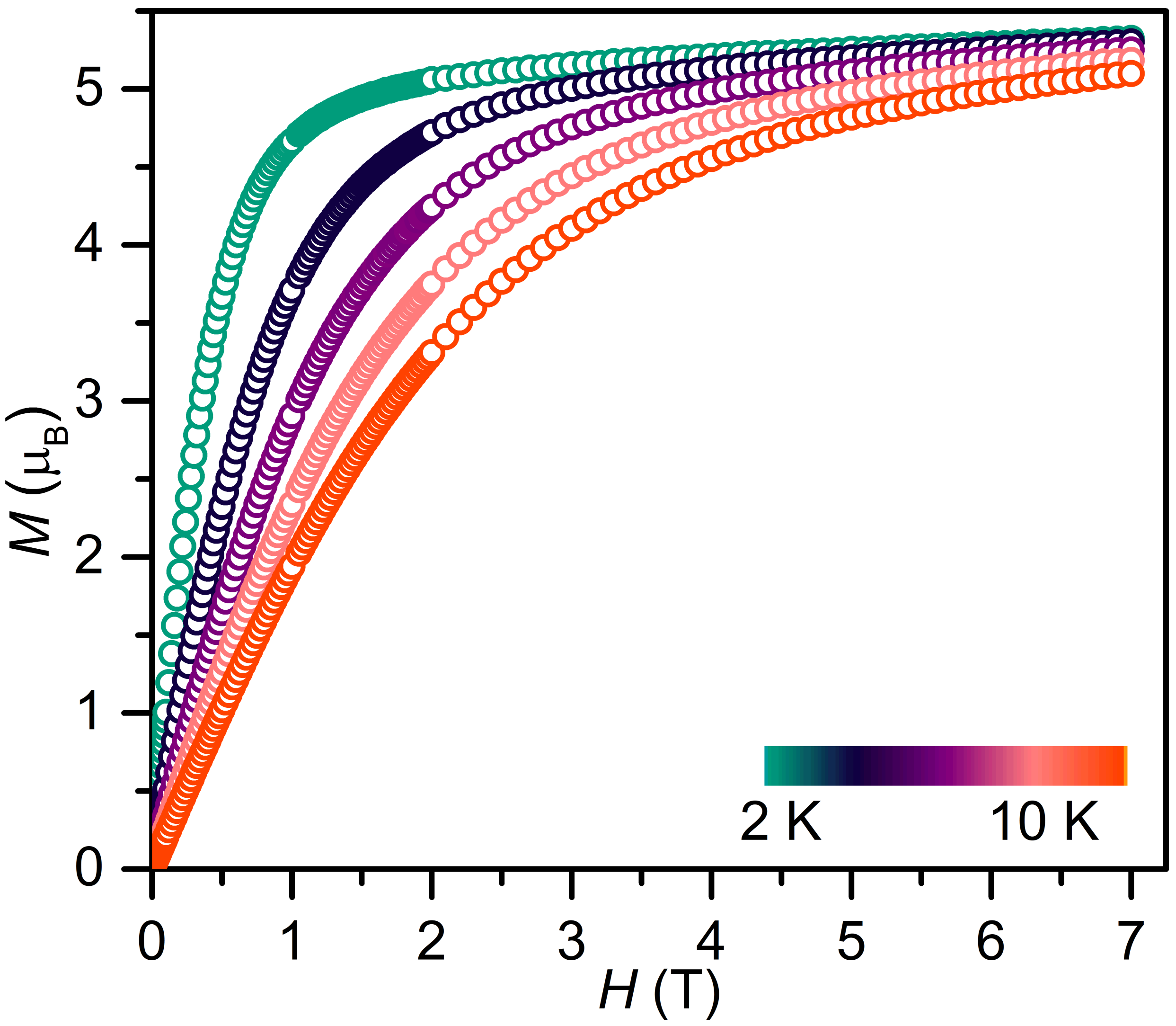

Figure S41. Variable-temperature field-dependent magnetisation curves recorded for {[$Cp^*{}_2$Tb(bpy)][BPh_4]}$_n$, **2**. Measurements were carried out between 0 and 7 T at 2, 4, 6, 8, and 10 K.

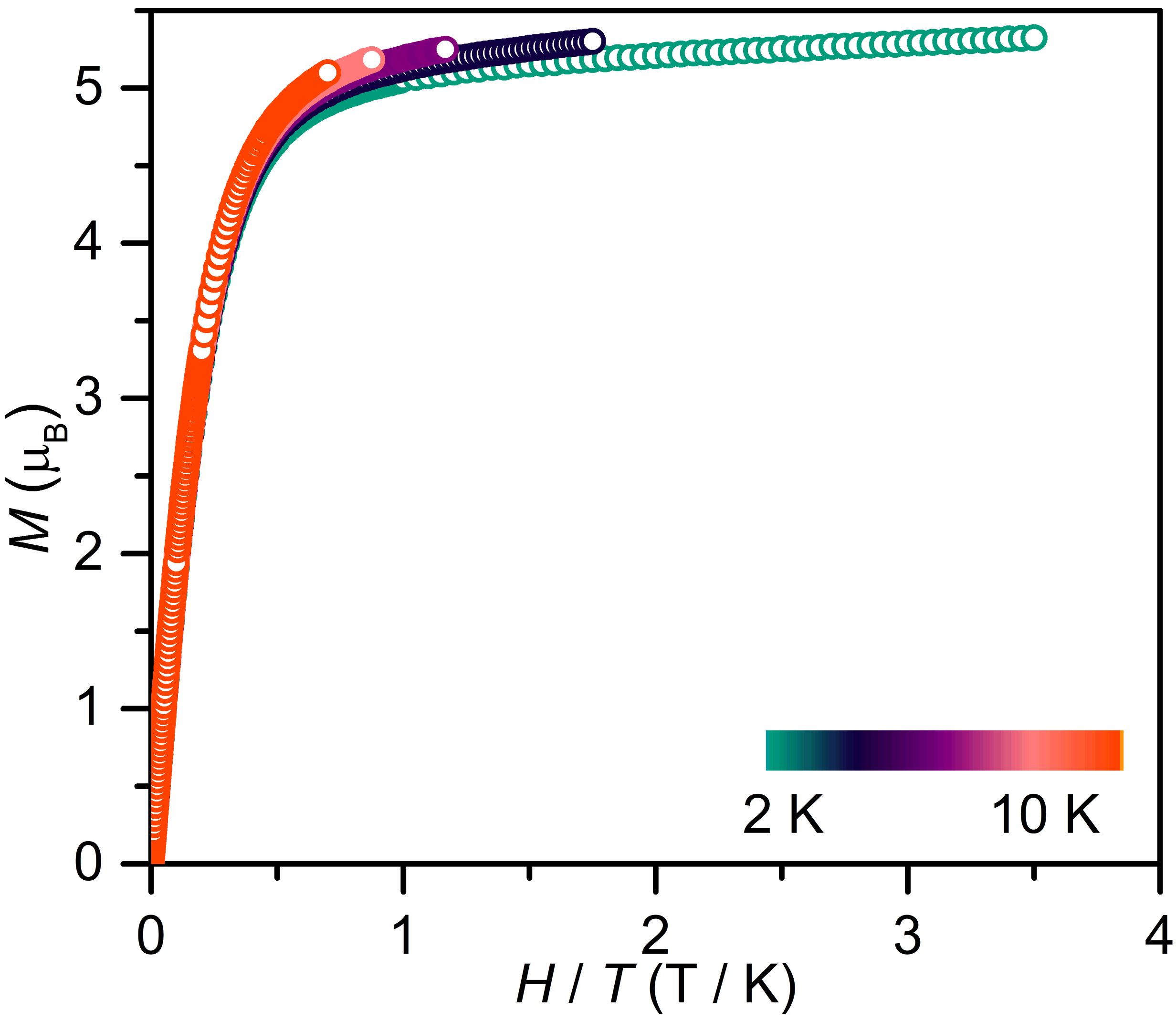

Figure S42. Reduced magnetisation data of $\{[Cp^*_2Tb(bpy)][BPh_4]\}_n$, **2**, collected from 0 to 7 T at 2, 4, 6, 8, and 10 K.

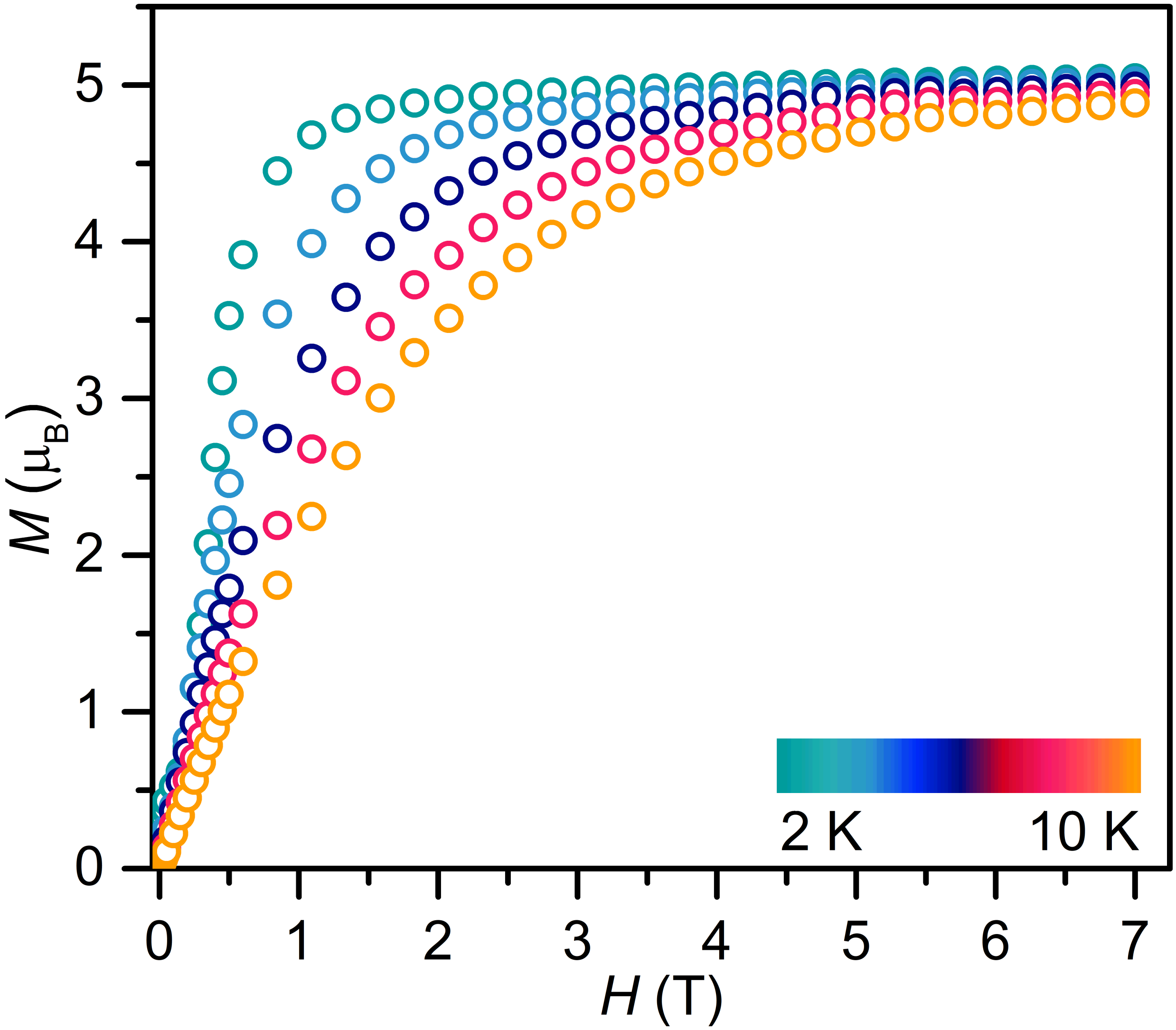

Figure S43. Variable-temperature field-dependent magnetisation curves recorded for {[Cp*$_2$Dy(bpy)][BPh$_4$]}$_n$, **3**. Measurements were carried out between 0 and 7 T at 2, 4, 6, 8, and 10 K.

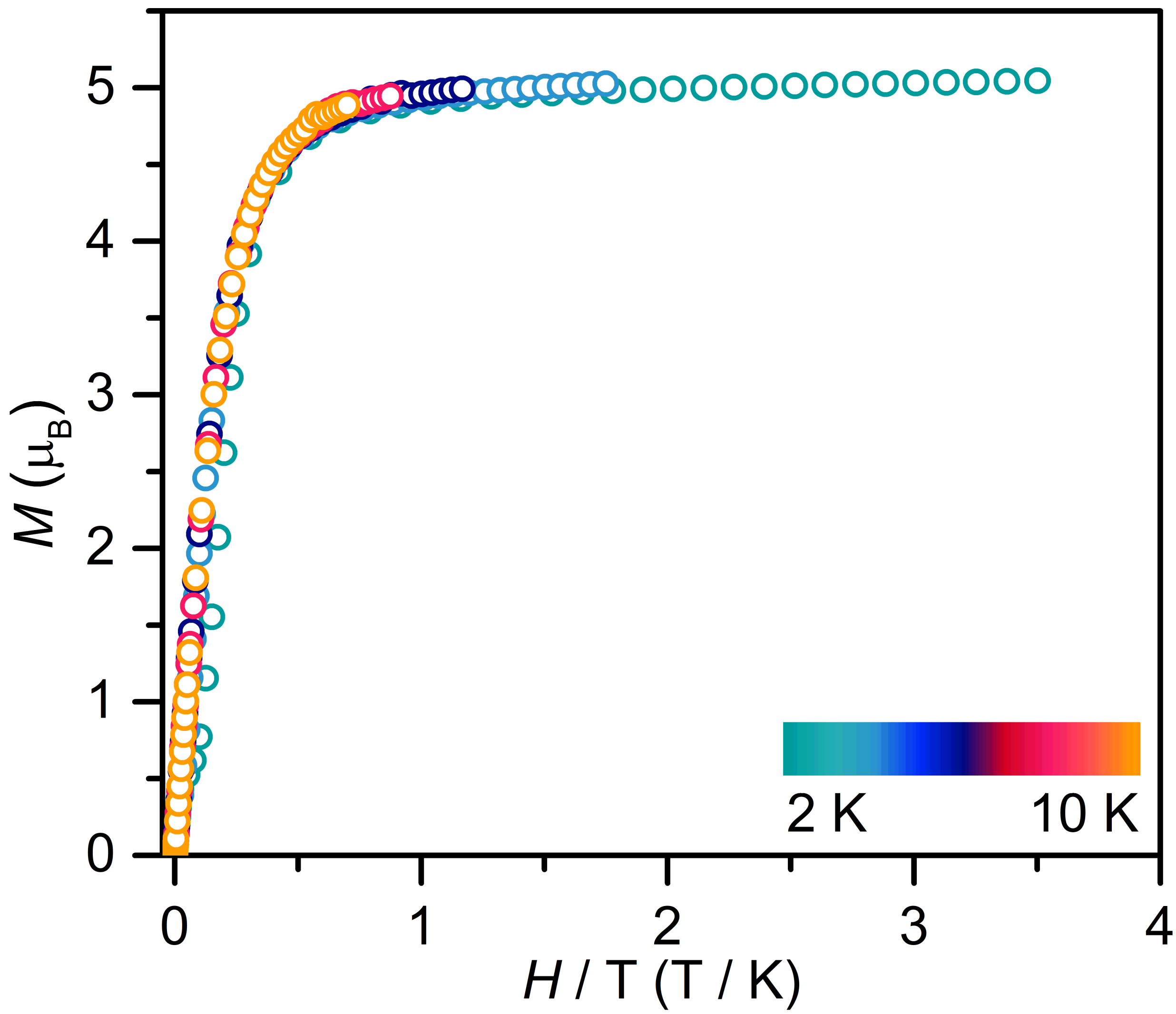

Figure S44. Reduced magnetisation data of {[Cp*$_2$Dy(bpy)][BPh$_4$]}$_n$, **3**, collected from 0 to 7 T at 2, 4, 6, 8, and 10 K.

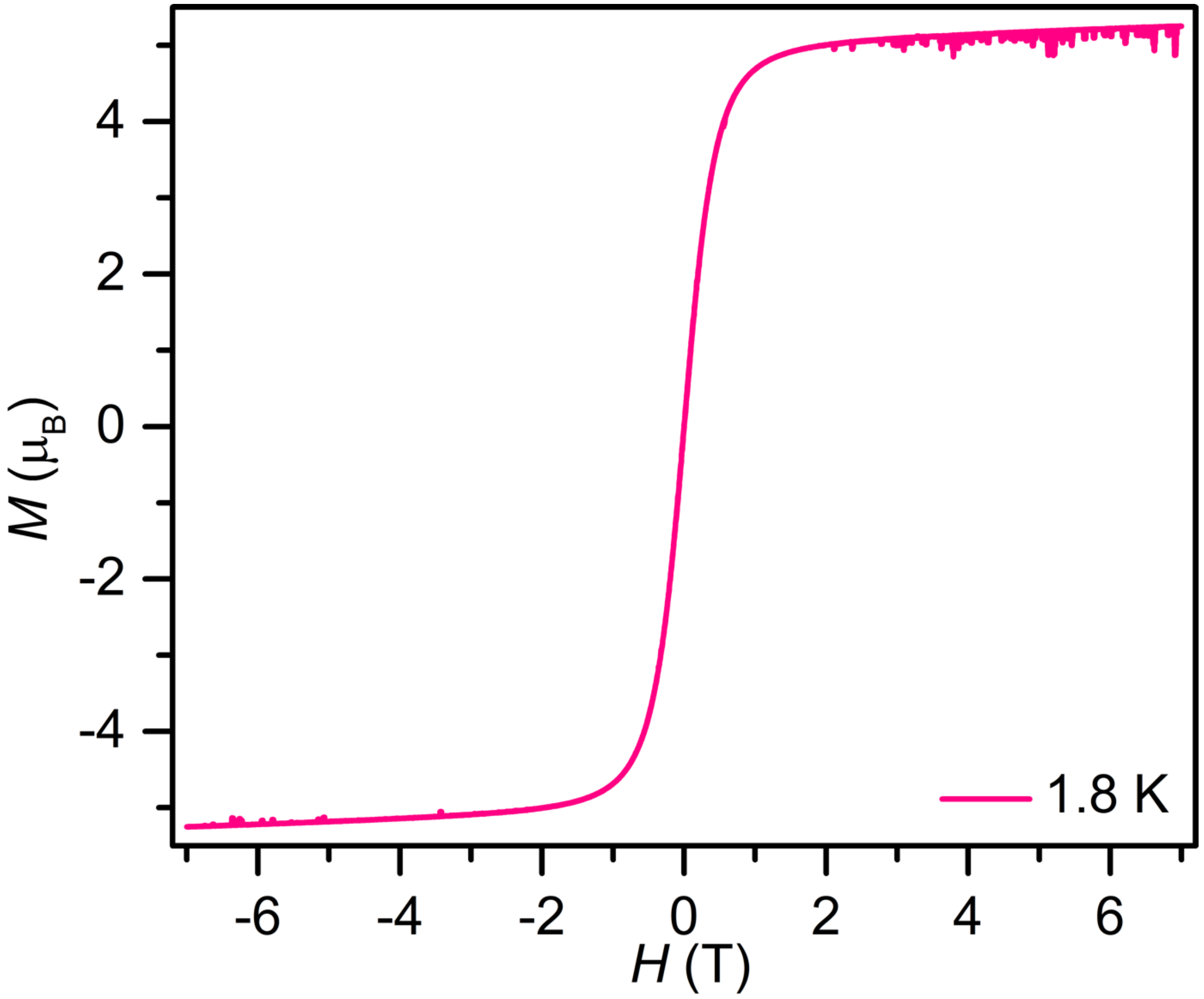

Figure S45. Plot of the magnetisation (*M*) *vs.* dc magnetic field (*H*) at an average sweep rate of 100 Oe/s for {[Cp*$_2$Tb(bpy)][BPh$_4$]}$_n$, **2**, at 1.8 K.

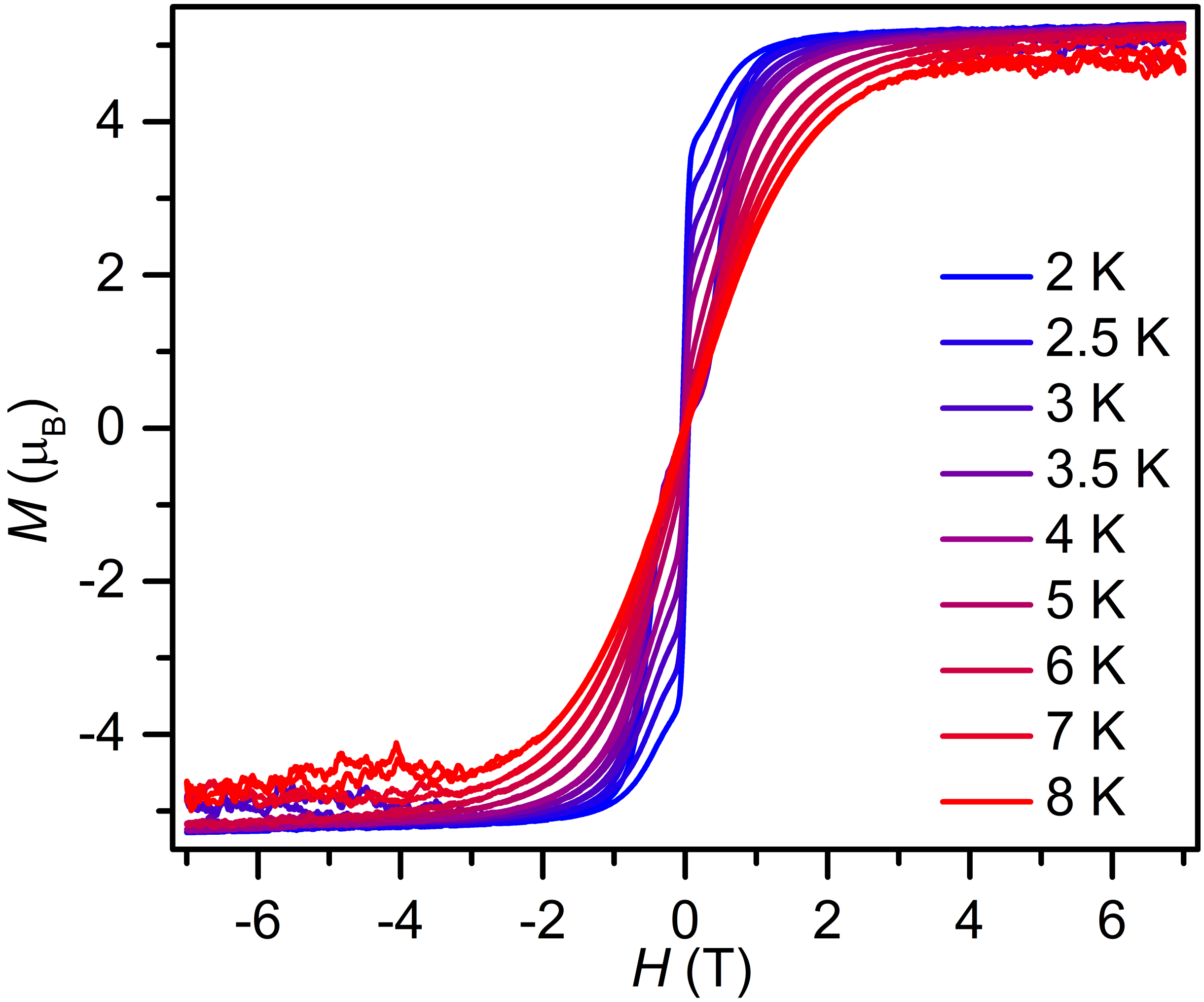

Figure S46. Plot of the magnetisation (*M*) *vs.* dc magnetic field (*H*) at an average sweep rate of 100 Oe/s for {[$Cp*_2Dy(bpy)$][BPh_4]}$_n$, **3**, from 2 K (blue line) to 8 K (red line).

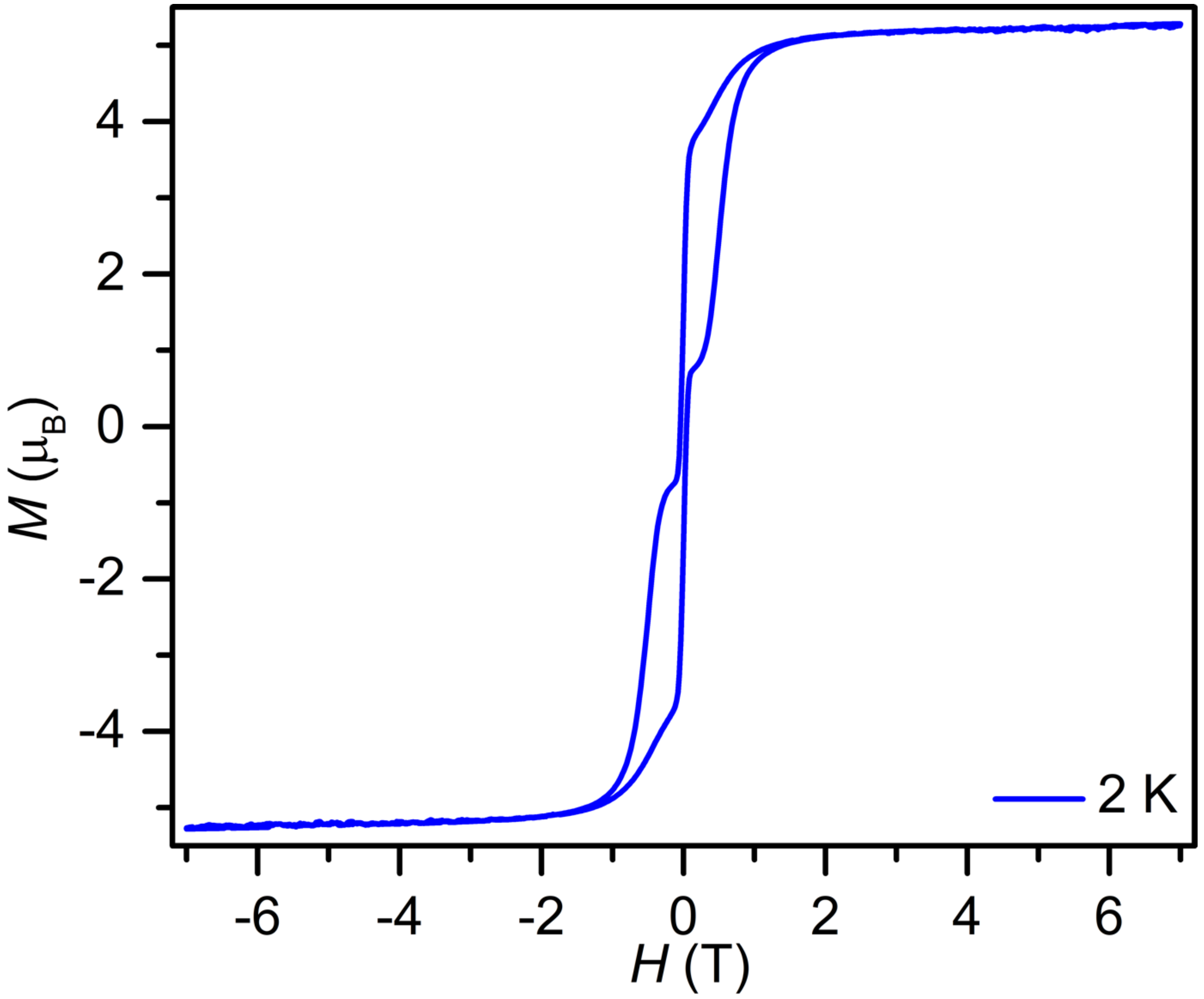

Figure S47. Plot of magnetisation (*M*) *vs* dc magnetic field (*H*) at an average sweep rate of 100 Oe/s for $\{[Cp^*_2Dy(bpy)][BPh_4]\}_n$, **3**, at 2 K.

4 DFT Calculations

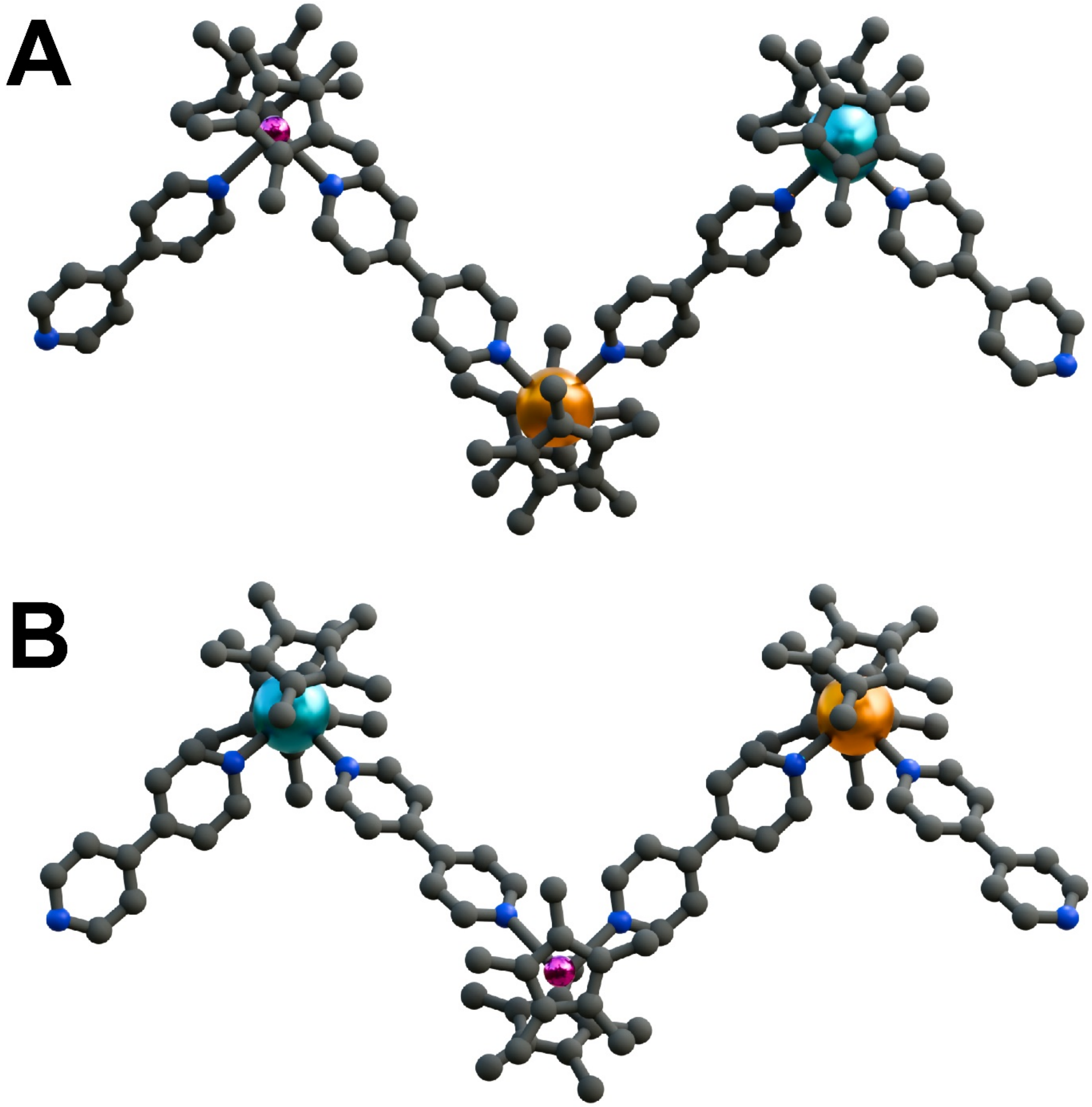

Figure S48. The two systems used to calculate the exchange coupling constant (*J*) between Gd^{III} centres along the chain structure of {[Cp*$_2$Gd(bpy)][BPh$_4$]}$_n$, **1**. The orange, pink, blue, and grey spheres represent Gd, Y, N, and C atoms, respectively. Teal and orange spherical shapes represent the spin densities. H atoms, solvent molecules in the crystal lattice, and the [BPh$_4$]$^-$ counter anions have been omitted for clarity. The depiction of spin density surfaces precludes the visibility of the Gd^{III} ions. Systems were used for (**A**) calculating *J* between adjacent Gd^{III} ions along the polymeric chain, (**B**) calculating *J* between Gd^{III} centres along the crystallographic *c* axis.

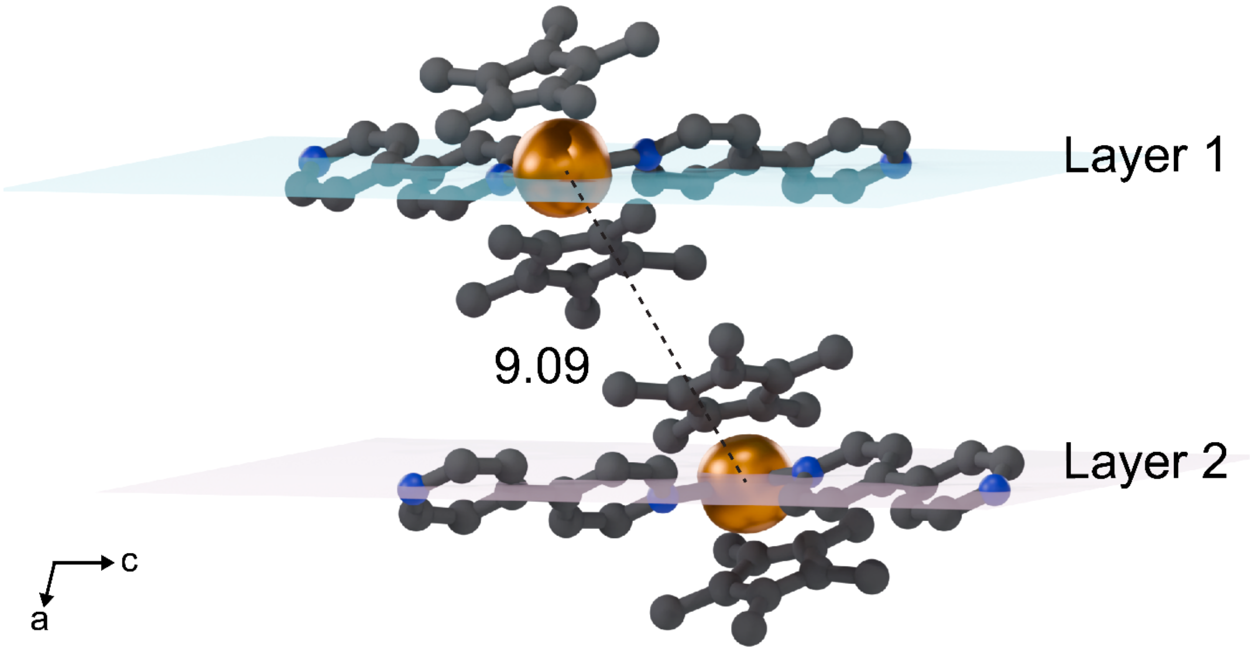

Figure S49. Calculation of exchange coupling constant (*J*) between Gd^{III} centres in adjacent layers in the structure $\{[Cp^*_2Gd(bpy)][BPh_4]\}_n$, **1**. The orange, blue, and grey spheres represent Gd, N, and C atoms, respectively. Orange spherical shapes represent the spin densities. H atoms, solvent molecules in the crystal lattice, and the $[BPh_4]^-$ counter anions have been omitted for clarity. The depiction of spin density surfaces precludes the visibility of the Gd^{III} ions. The distance is given in Å.

5 EPR Spectroscopy

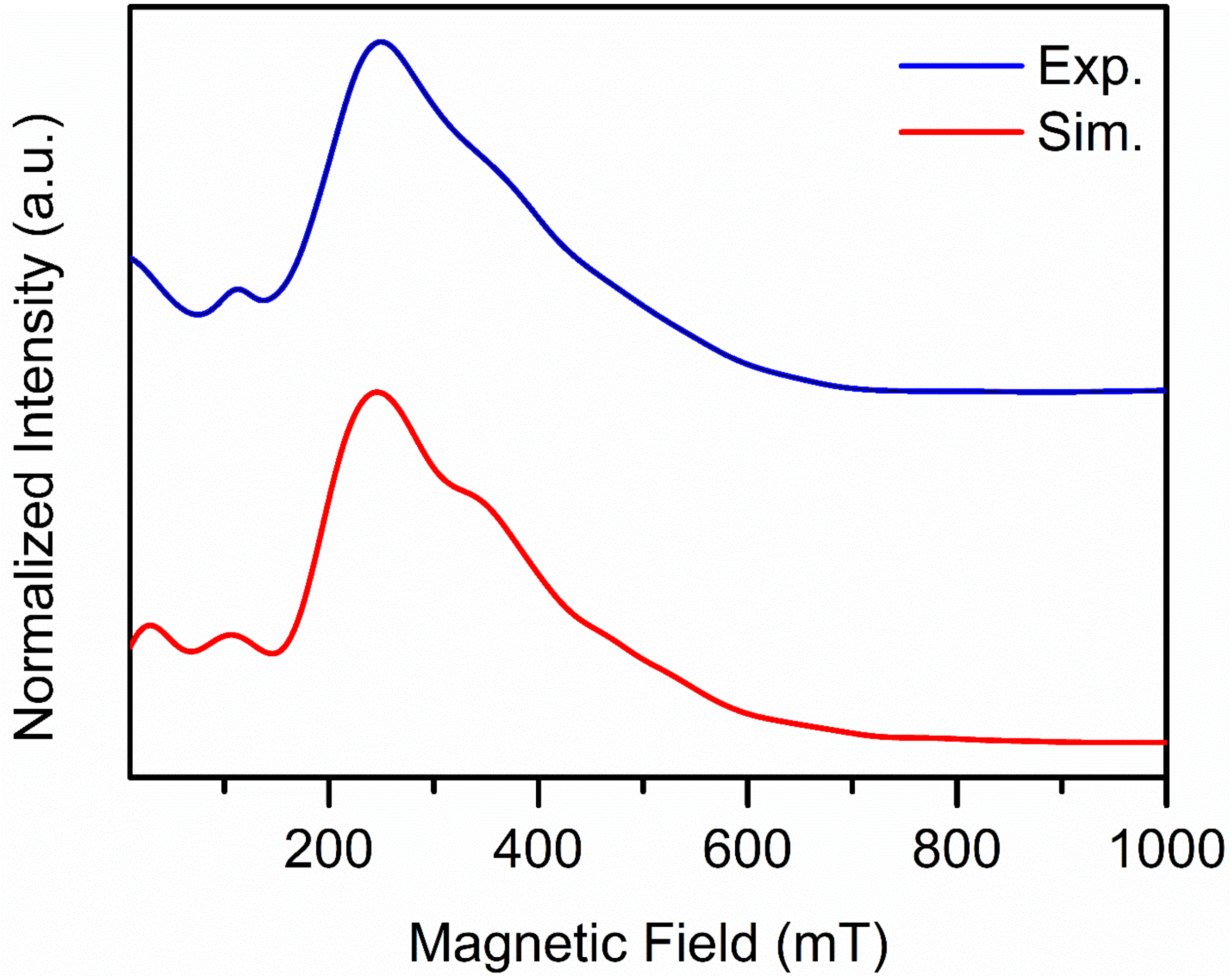

Figure S50. First integral of the X-band cw-EPR spectrum of $\{[Cp^{*}_{2}Gd(bpy)][BPh_{4}]\}_{n}$, **1**, collected at 110 K (blue line). The simulated spectrum (red line) was obtained considering the values of S = 7/2, g = 2.08718, D = 0.09186 cm^{-1}, E = 0.02269 cm^{-1} and lwpp = 48.

6 *Ab initio* Calculations

Table S5. Calculated Kramers doublet (KD) energies, associated magnetic moments, *g*-tensors, and wavefunction composition for $[Cp^*{}_2Dy(bpy)_2]^+$. Only contributions > 5% are printed. The wavefunction decompositions correspond to the lowest atomic multiplet *J* = 15/2 in wave functions with definite projection of the total moment to the quantisation axis.

KD	E (cm^{-1})	*M* (μ_B)	g_x	g_y	g_z	Wave function composition
**1**	0.0	9.780	0.0007	0.0027	19.5595	$\|\pm15/2\rangle$ (94%)
**2**	161.2	8.444	0.0309	0.0343	16.9181	$\|\pm13/2\rangle$ (95%)
**3**	319.7	6.577	1.5119	2.3848	13.4246	$\|\pm15/2\rangle$ (5%); $\|\pm11/2\rangle$ (79%); $\|\pm9/2\rangle$ (5%); $\|\pm3/2\rangle$ (6%)
**4**	373.3	0.665	1.3090	3.5849	12.5896	$\|\pm11/2\rangle$ (9%); $\|\pm9/2\rangle$ (12%); $\|\pm5/2\rangle$ (10%); $\|\pm3/2\rangle$ (21%); $\|\pm1/2\rangle$ (44%)
**5**	417.3	4.354	1.2572	1.5977	8.7242	$\|\pm9/2\rangle$ (48%); $\|\pm7/2\rangle$ (18%); $\|\pm5/2\rangle$ (20%)
**6**	434.2	2.916	2.1421	4.1048	8.9312	$\|\pm9/2\rangle$ (23%); $\|\pm7/2\rangle$ (37%); $\|\pm5/2\rangle$ (16%); $\|\pm3/2\rangle$ (23%);
**7**	499.5	1.001	0.7487	1.2804	16.6825	$\|\pm9/2\rangle$ (8%); $\|\pm7/2\rangle$ (36%); $\|\pm5/2\rangle$ (37%); $\|\pm3/2\rangle$ (14%);
**8**	708.5	0.163	0.0128	0.0220	19.7968	$\|\pm7/2\rangle$ (6%); $\|\pm5/2\rangle$ (17%); $\|\pm3/2\rangle$ (33%); $\|\pm1/2\rangle$ (43%);

Table S6. Crystal field parameters calculated for $[Cp^*_2Dy(bpy)_2]^+$ via the SINGLE_ANISO program. The Hamiltonian employed to calculate the crystal field parameters is given by:

$$\hat{H}_{CF} = \sum_{k=2,4,6} \sum_{k=-q}^{+q} \left[B_k^q \hat{O}_k^q(S)\right]$$

where $\hat{O}_k^q$ is the extended Stevens operator, B_k^q the crystal field parameter, *k* is the rank of the irreducible tensor operator (ITO) (2,4,6), *q* is the component of the ITO (*q* = −*k*, −*k*+1,…0,1,…*k*). Weights are explicitly printed for all B_q^k > 1% weight.

k	***q***	**Weight (%)**	B_q^k	***k***	***q***	**Weight (%)**	B_q^k
2	−2	<1	-1.06E-05	6	−6	<1	4.01E-09
	−1	<1	-4.23E-05		−5	<1	-4.02E-09
	0	33	-2.98E+00		−4	<1	-1.56E-09
	1	4	-8.54E-01		−3	<1	-2.55E-09
	2	23	2.53E+00		−2	<1	4.90E-09
4	−4	<1	6.83E-07		−1	<1	4.75E-10
	−3	<1	-2.40E-07		0	1	7.36E-06
	−2	<1	1.68E-08		1	1	4.64E-05
	−1	<1	2.82E-07		2	12	3.41E-04
	0	5	-2.46E-03		3	1	-5.73E-05
	1	<1	1.09E-03		4	<1	2.61E-05
	2	1	-1.92E-03		5	<1	-1.21E-04
	3	<1	1.19E-03		6	<1	2.62E-05
	4	11	2.25E-02				

Table S7. Calculated average transition dipole moments for the eight lowest lying Kramers doublets with opposing magnetisation (+*I* → *I*−1), and for excited states (*I* → *I*+1), (*I* → *I*+2), (*I* → *I*+3), (*I* → *I*+4) and (*I* → *I*+5) of $[Cp^*_2Dy(bpy)_2]^+$.

**Through Barrier (+*I* → −*I*)**			**Through Excited States (+*I* → *I*+1)**			**Through Excited States (+*I* → *I*+2)**		
**KD1**	**KD2**	**Magnitude**	**KD**	**KD+1**	**Magnitude**	**KD**	**KD+2**	**Magnitude**
+1	−1	5.68E-04	+1	+2	1.78E+00	+1	+3	4.02E-01
+2	−2	1.09E-02	+1	−2	1.71E-03	+1	−3	1.88E-02
+3	−3	6.55E-01	+2	+3	2.22E+00	+2	+4	5.05E-01
+4	−4	2.69E+00	+2	−3	6.11E-02	+2	−4	4.50E-01
+5	−5	4.76E-01	+3	+4	1.94E+00	+3	+5	1.94E+00
+6	−6	1.23E+00	+3	−4	1.27E+00	+3	−5	1.91E-01
+7	−7	1.25E+00	+4	+5	1.98E+00	+4	+6	1.55E+00
+8	−8	1.33E-01	+4	−5	1.25E+00	+4	−6	9.89E-01
			+5	+6	2.61E+00	+5	+7	8.54E-01
			+5	−6	1.26E+00	+5	−7	7.46E-01
			+6	+7	1.87E+00	+6	+8	2.78E-01
			+6	−7	1.14E+00	+6	−8	1.06E-01
			+7	+8	1.63E+00			
			+7	−8	3.44E-01			

**Through Excited States (+*I* → *I*+3)**			**Through Excited States (+*I* → *I*+4)**			**Through Excited States (+*I* → *I*+5)**		
**KD**	**KD+3**	**Magnitude**	**KD**	**KD+4**	**Magnitude**	**KD**	**KD+5**	**Magnitude**
+1	+4	1.02E-01	+1	+5	2.63E-01	+1	+6	2.15E-01
+1	−4	1.10E-01	+1	−5	1.44E-02	+1	−6	4.83E-02
+2	+5	4.63E-01	+2	+6	2.46E-01	+2	+7	1.54E-01
+2	−5	5.06E-02	+2	−6	1.23E-01	+2	−7	9.73E-02
+3	+6	7.37E-01	+3	+7	2.36E-01	+3	+8	6.34E-02
+3	−6	4.01E-01	+3	−7	2.66E-01	+3	−8	3.04E-02
+4	+7	8.36E-01	+4	+8	1.08E-01			
+4	−7	6.09E-01	+4	−8	9.15E-02			
+5	+8	1.85E-01						
+5	−8	1.42E-01						

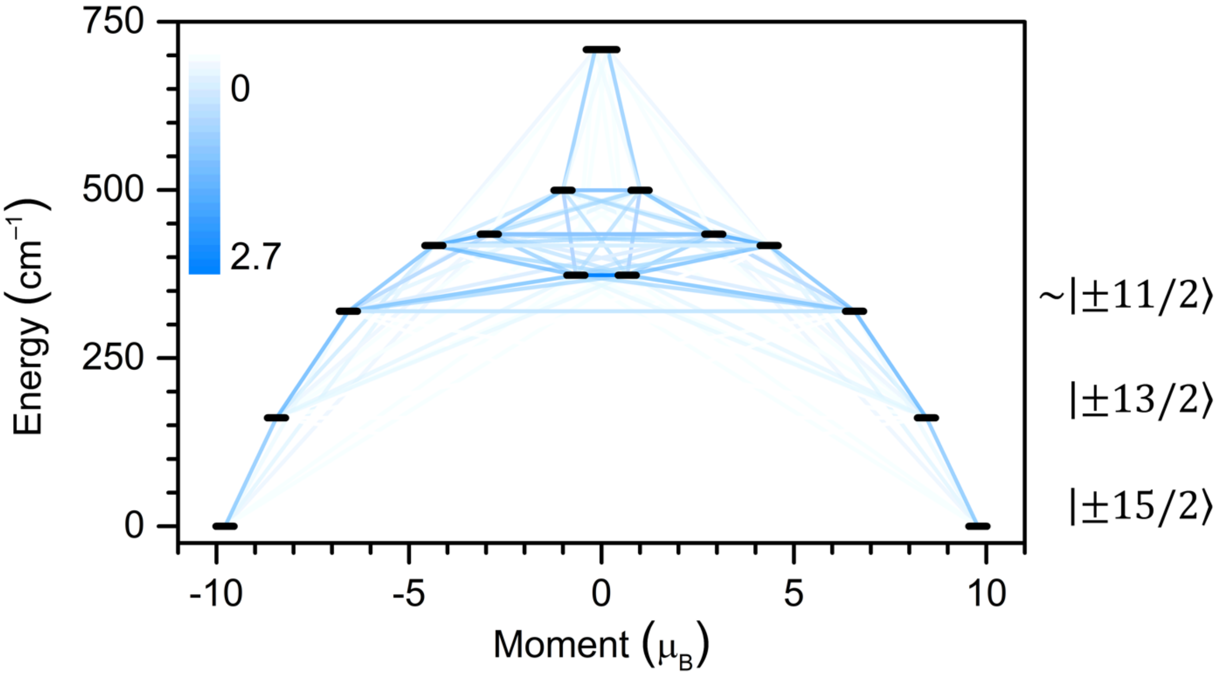

Figure S51. Calculated relaxation barrier for $[Cp^*_2Dy(bpy)_2]^+$. Solid lines represent possible relaxation processes as indicated by calculated transition magnetic dipole moments, where more intense blue colouration represents the most probable transitions whereas faded blue colouration indicates vanishing probabilities. The numbers on the right are given for the primary M_J state comprising the wave function for each state.

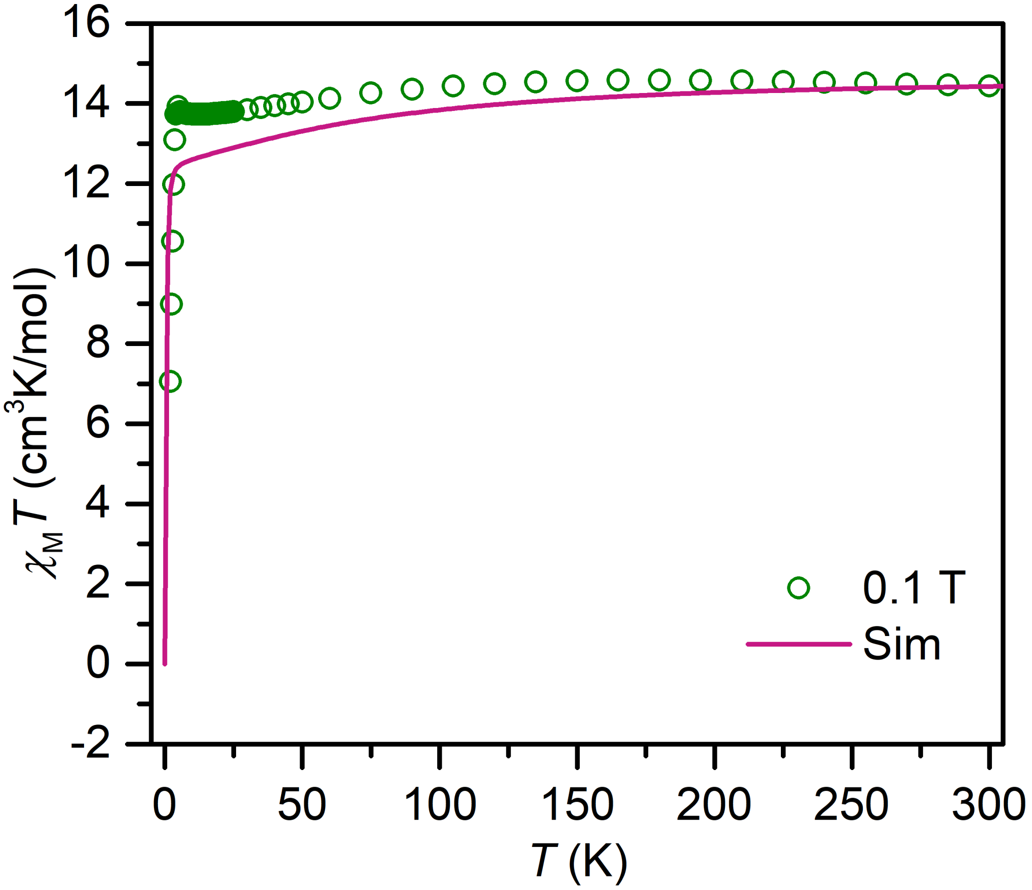

Figure S52. Variable-temperature dc magnetic susceptibility data of $\{[Cp^*_2Dy(bpy)][BPh_4]\}_n$, **3** (green circles), collected under a 0.1 T applied dc field. Solid purple line represents the calculated values for the $[Cp^*_2Dy(bpy)_2]^+$ fragment in **3**.

Table S8. Coordinates of the optimised Dy chain fragment $[Cp^*_2Dy(bpy)_2]^+$.

Dy	6.613189	6.496342	3.724620
N	3.284570	13.173716	9.817190
C	2.716868	11.956546	9.644752
H	1.828037	11.726296	10.238186
C	4.313310	13.363765	9.015232
H	4.825040	14.328050	9.032955
C	3.122487	10.987075	8.743540
C	4.766569	12.422055	8.107902
H	2.505541	10.091898	8.767572
C	4.168887	11.198475	7.944504
H	5.628500	12.760146	7.541747
C	4.662570	10.190574	6.968510
C	5.708970	10.401974	6.169474
C	4.064898	8.966994	6.805112
C	6.114589	9.432513	5.268261
H	6.297027	11.313682	6.175825
H	3.203943	8.661026	7.393285
C	4.518147	8.025284	5.897782
N	5.546887	8.215333	5.095824
H	6.989755	9.654567	4.671149
H	3.998703	7.075670	5.868500
C	4.093768	5.699842	3.119108
C	9.132611	5.699843	4.330132
C	4.462143	6.611652	2.131607
C	8.764236	6.611653	5.317632
C	5.490912	6.045781	1.338589
C	7.735477	6.045784	6.110651
C	5.784135	4.773082	1.875465
C	7.442244	4.773083	5.573776
C	4.937826	4.583032	2.983733
C	8.288553	4.583033	4.465508
C	2.895027	5.725473	4.048267
C	10.331352	5.725472	3.400973
C	3.699457	7.903572	1.958321
C	9.526922	7.903573	5.490917
C	6.049222	6.624471	0.064410
C	7.177157	6.624474	7.384829
C	6.717208	3.735281	1.295815
C	6.509171	3.735284	6.153427
C	4.696879	3.286853	3.742863
C	8.529500	3.286852	3.706380
H	2.074218	5.162900	3.496536
H	2.997211	5.134897	4.966921
H	2.435566	6.708828	4.192848
H	11.152163	5.162905	3.952710

H	10.229174	5.134888	2.482323
H	10.790813	6.708827	3.256389
H	4.204760	8.664981	1.358484
H	2.775822	7.657728	1.358956
H	3.298121	8.295758	2.898136
H	9.021619	8.664982	6.090754
H	10.450558	7.657731	6.090282
H	9.928258	8.295761	4.551102
H	5.889005	7.697834	-0.054665
H	7.051732	6.264082	-0.203478
H	5.423913	6.163620	-0.768580
H	7.337373	7.697838	7.503903
H	6.174644	6.264086	7.652706
H	7.802460	6.163625	8.217823
H	6.867854	2.849901	1.914054
H	6.192806	3.295387	0.387604
H	7.632611	4.125692	0.827205
H	6.358526	2.849902	5.535190
H	7.033575	3.295393	7.061638
H	5.593768	4.125694	6.622038
H	4.231222	3.379027	4.732103
H	3.846452	2.764752	3.168290
H	5.466864	2.513044	3.652202
H	8.995157	3.379025	2.717139
H	9.379926	2.764750	4.280951
H	7.759514	2.513044	3.797042
N	7.679492	8.215332	2.353414
C	7.111790	9.432512	2.180975
H	6.236625	9.654565	2.778088
C	8.708232	8.025281	1.551456
H	9.227676	7.075667	1.580738
C	7.517410	10.401971	1.279762
C	9.161482	8.966991	0.644125
H	6.929354	11.313680	1.273408
C	8.563809	10.190571	0.480725
H	10.022435	8.661021	0.055951
C	9.057493	11.198470	-0.495269
C	10.103892	10.987070	-1.294305
C	8.459820	12.422050	-0.658669
C	10.509512	11.956539	-2.195518
H	10.720863	10.091908	-1.318318
H	7.597891	12.760150	-0.092517
C	8.913070	13.363760	-1.565999
N	9.941810	13.173709	-2.367957
H	11.398376	11.726369	-2.788934
H	8.401318	14.328038	-1.583660

7 References

1 R. D. Shannon, Revised effective ionic radii and systematic studies of interatomic distances in halides and chalcogenides, *Acta Crystallogr. A*, 1976, **32**, 751–767.

2 S. Demir, J. M. Zadrozny and J. R. Long, Large Spin-Relaxation Barriers for the Low-Symmetry Organolanthanide Complexes [$Cp^*_2Ln(BPh_4)$] (Cp*=pentamethylcyclopentadienyl; Ln=Tb, Dy), *Chem. Eur. J.*, 2014, **20**, 9524–9529.

3 S. Demir, M. D. Boshart, J. F. Corbey, D. H. Woen, M. I. Gonzalez, J. W. Ziller, K. R. Meihaus, J. R. Long and W. J. Evans, Slow Magnetic Relaxation in a Dysprosium Ammonia Metallocene Complex, *Inorg. Chem.*, 2017, **56**, 15049–15056.

4 S. C. Corner, G. K. Gransbury, I. J. Vitorica-Yrezabal, G. F. S. Whitehead, N. F. Chilton and D. P. Mills, Halobenzene Adducts of a Dysprosocenium Single-Molecule Magnet, *Inorg. Chem.*, 2024, **63**, 9552–9561.

5 S. C. Corner, G. K. Gransbury, I. J. Vitorica-Yrezabal, G. F. S. Whitehead, N. F. Chilton and D. P. Mills, Synthesis and Magnetic Properties of Bis-Halobenzene Decamethyldysprosocenium Cations, *Inorg. Chem.*, 2024, **63**, 9562–9571.

6 D. Errulat, B. Gabidullin, A. Mansikkamäki and M. Murugesu, Two heads are better than one: improving magnetic relaxation in the dysprosium metallocene upon dimerization by use of an exceptionally weakly-coordinating anion, *Chem. Commun.*, 2020, **56**, 5937–5940.

7 C. G. T. Price, A. Mondal, J. P. Durrant, J. Tang and R. A. Layfield, Structural and Magnetization Dynamics of Borohydride-Bridged Rare-Earth Metallocenium Cations, *Inorg. Chem.*, 2023, **62**, 9924–9933.

8 Q. Yuan, Y.-S. Meng, Y.-Q. Zhang, C. Gao, S.-S. Liu, B.-W. Wang and S. Gao, Synthesis and structures of fluoride-bridged dysprosium clusters: influence of fluoride ions on magnetic relaxation behaviors, *Inorg. Chem. Front.*, 2022, **9**, 2336–2342.

9 Y. Meng, J. Xiong, M. Yang, Y. Qiao, Z. Zhong, H. Sun, J. Han, T. Liu, B. Wang and S. Gao, Experimental Determination of Magnetic Anisotropy in Exchange-Bias Dysprosium Metallocene Single-Molecule Magnets, *Angew. Chem. Int. Ed.*, 2020, **59**, 13037–13043.

10 T. Pugh, V. Vieru, L. F. Chibotaru and R. A. Layfield, Magneto-structural correlations in arsenic- and selenium-ligated dysprosium single-molecule magnets, *Chem. Sci.*, 2016, **7**, 2128–2137.

11 F. Benner, L. La Droitte, O. Cador, B. Le Guennic and S. Demir, Magnetic hysteresis and large coercivity in bisbenzimidazole radical-bridged dilanthanide complexes, *Chem. Sci.*, 2023, **14**, 5577–5592.

12 P. Evans, D. Reta, C. A. P. Goodwin, F. Ortu, N. F. Chilton and D. P. Mills, A double-dysprosocenium single-molecule magnet bound together with neutral ligands, *Chem. Commun.*, 2020, **56**, 5677–5680.
